# Response Rate and Safety of a Neoadjuvant Pertuzumab, Atezolizumab, Docetaxel, and Trastuzumab Regimen for Patients With *ERBB2*-Positive Stage II/III Breast Cancer: The Neo-PATH Phase 2 Nonrandomized Clinical Trial

**DOI:** 10.1001/jamaoncol.2022.2310

**Published:** 2022-07-07

**Authors:** Hee Kyung Ahn, Sung Hoon Sim, Koung Jin Suh, Min Hwan Kim, Jae Ho Jeong, Ji-Yeon Kim, Dae-Won Lee, Jin-Hee Ahn, Heejung Chae, Kyung-Hun Lee, Jee Hyun Kim, Keun Seok Lee, Joo Hyuk Sohn, Yoon-La Choi, Seock-Ah Im, Kyung Hae Jung, Yeon Hee Park

**Affiliations:** 1 Division of Medical Oncology, Department of Internal Medicine, Gachon University Gil Medical Center, Incheon, Korea; 2 Center for Breast Cancer, National Cancer Center, Goyang, Korea; 3 Department of Internal Medicine, Seoul National University Bundang Hospital, Seoul National University College of Medicine, Seongnam, Korea; 4 Division of Medical Oncology, Department of Internal Medicine, Yonsei University College of Medicine, Seoul, Korea; 5 Department of Oncology, Asan Medical Center, University of Ulsan College of Medicine, Seoul, Korea; 6 Division of Hematology-Oncology, Department of Medicine, Samsung Medical Center, Sungkyunkwan University School of Medicine, Seoul, Korea; 7 Department of Internal Medicine, Seoul National University Hospital, Cancer Research Institute, Seoul National University College of Medicine, Seoul, Korea; 8 Department of Pathology and Translational Genomics, Samsung Medical Center, Sungkyunkwan University School of Medicine, Seoul, Korea

## Abstract

**Importance:**

Addition of immune checkpoint inhibitors to anti-*ERBB2* treatment has shown synergistic efficacy in preclinical studies and is thus worth investigating as a neoadjuvant treatment to maximize efficacy and to minimize toxic effects.

**Objective:**

To determine if neoadjuvant atezolizumab, docetaxel, trastuzumab, and pertuzumab therapy for *ERBB2*-positive early breast cancer warrants continuation to the next phase.

**Design, Setting, and Participants:**

This nonrandomized, open label, multicenter, phase 2 trial was conducted by the Korean Cancer Study Group and enrolled patients across 6 institutions in Korea from May 2019 to May 2020. Eligible patients were diagnosed with *ERBB2*-positive breast cancer (primary tumor size >2 cm or pathologically confirmed lymph node–positive cancer, without distant metastases) with a clinical stage of II or III.

**Interventions:**

Patients received 6 cycles of neoadjuvant pertuzumab (840 mg at first cycle, 420 mg during subsequent cycles), atezolizumab (1200 mg), docetaxel (75 mg/m^2^), and trastuzumab (600 mg via subcutaneous injection) every 3 weeks, followed by surgery. Patients with pathologic complete response (pCR) received 12 cycles of adjuvant atezolizumab, trastuzumab, and pertuzumab every 3 weeks after surgery. Patients without pCR were treated with 14 cycles of atezolizumab, 1200 mg, plus trastuzumab emtansine, 3.6 mg/kg, every 3 weeks.

**Main Outcomes and Measures:**

The primary end point was pCR rate, which was defined as the absence of invasive cancer cells in the primary tumor and regional lymph nodes (ypT0/isN0). Secondary end points included clinical objective response rate, 3-year event-free survival rate according to pCR achievement, disease-free survival, overall survival, toxic effects, and quality-of-life outcomes.

**Results:**

A total of 67 women (median [range] age, 52 [33-74] years) were enrolled. Hormone receptor expression was positive in 32 (48%) patients. Curative surgery was performed in 65 patients because 2 patients showed disease progression during neoadjuvant treatment and their tumors became unresectable. The overall pCR rate was 61% (41 of 67 patients). The pCR rate was higher in hormone receptor–negative disease vs hormone receptor–positive disease (27 of 35 [77%] patients vs 14 of 32 [44%] patients) and in programmed cell death 1–positive expression vs programmed cell death 1–negative expression (13 of 13 [100%] patients vs 28 of 53 [53%] patients). Grade 3 and 4 neutropenia and febrile neutropenia occurred in 8 (12%) patients and 5 (8%) patients, respectively. Grade 3 and 4 immune-related adverse events occurred in only 4 patients (grade 3 skin rash, encephalitis, hepatitis, and fever). No treatment-related death occurred during the neoadjuvant phase.

**Conclusions and Relevance:**

In this nonrandomized clinical trial, treatment with the neoadjuvant atezolizumab, docetaxel, trastuzumab, and pertuzumab regimen in patients with stage II or III *ERBB2*-positive breast cancer appears to have had an acceptable pCR rate and modest toxic effects. Further investigation of this immunotherapy combination in *ERBB2*-positive early breast cancer is warranted.

**Trial Registration:**

ClinicalTrials.gov Identifier: NCT03881878

## Key Points

**Question:**

What are the outcomes of neoadjuvant atezolizumab with docetaxel, trastuzumab, and pertuzumab (PATH) for the treatment of *ERBB2*-positive early breast cancer?

**Findings:**

In this single-arm, phase 2, nonrandomized clinical trial of 67 patients with *ERBB2*-positive early breast cancer, the overall pathologic complete response rate of 6 cycles of neoadjuvant PATH regimen was 61%. During the neoadjuvant phase, the incidence rate of febrile neutropenia was 8% and grade 3 or 4 immune-related adverse events occurred in 4 patients.

**Meaning:**

Use of the neoadjuvant PATH regimen for *ERBB2*-positive EBC warrants further investigation.

## Teaser

This nonrandomized clinical trial aims to determine whether neoadjuvant atezolizumab, docetaxel, trastuzumab, and pertuzumab therapy for *ERBB2*-positive early breast cancer warrants continuation to the next phase.

## Introduction

Presence of tumor-infiltrating lymphocytes in *ERBB2*-positive breast cancer and trastuzumab-associated antibody-dependent cellular cytotoxicity has suggested immunogenic potential of anti-*ERBB2* therapy in *ERBB2*-positive breast cancer,^1,2,3^ with synergistic efficacy shown in preclinical studies.^4,5^ Preoperative dual blockade of *ERBB2* using trastuzumab and pertuzumab dramatically increased the pathologic complete response (pCR) rate in individuals with *ERBB2*-positive early breast cancer.^6,7,8^ Docetaxel, carboplatin, trastuzumab, and pertuzumab (TCHP), designated as one of the preferred regimens by the National Comprehensive Cancer Network guidelines and which is being widely used in clinical practice, demonstrated a pCR (ypT0/isN0) rate of 63.6% in the TRYPHAENA phase 2 trial^8^ and 56% in the KRISTINE phase 3 trial.^7^ However, this increased efficacy is in exchange for increased toxic effects, especially severe myelosuppression even with filgrastim support, gastrointestinal toxic effects, and peripheral neuropathy, which makes it difficult to administer this therapy to elderly patients and patients with comorbidities.

We hypothesized that if adding an immune checkpoint inhibitor to anti-*ERBB2* treatment enhances the treatment efficacy in patients with *ERBB2*-positive breast cancer, it could replace carboplatin in the TCHP regimen to improve safety profiles without compromising efficacy by mitigating severe toxic effects from dual cytotoxic chemotherapy. This nonrandomized clinical trial aimed to evaluate the feasibility of the pertuzumab, atezolizumab, docetaxel, and trastuzumab (PATH) combination as a neoadjuvant treatment in patients with *ERBB2*-positive early breast cancer and whether it warrants continuation to the next phase.

## Methods

### Study Design and Patient Population

Neo-PATH (KCSG BR18-23) was an investigator-initiated, multi-institutional, open-label, single-arm phase 2 study by the Korean Cancer Study Group across 6 institutions in Korea to evaluate the efficacy and safety of the PATH combination for treatment of *ERBB2*-positive early breast cancer with a clinical stage of II or III. Eligible patients were 19 years or older, female, had a histological diagnosis of *ERBB2*-positive breast cancer without distant metastases, and had a primary tumor size larger than 2 cm or larger or regional axillary lymph node metastases that were histologically or cytologically confirmed (clinical stage of IIA-IIIC according to the American Joint Committee on Cancer’s TNM staging system, 7th edition). *ERBB2* status was assessed locally, and a positive *ERBB2 *status was defined as a score of 3 or higher on immunohistochemistry or positive result on in situ hybridization in the case of tumors with an immunohistochemistry score of 2 or higher. Other inclusion criteria were an Eastern Cooperative Oncology Group performance status score of 0 or 1, a left ventricular ejection fraction rate of 55% or greater as assessed by echocardiography at baseline, and adequate organ functions.

The study protocol (Supplement 1) was approved by the institutional review boards of all study sites and the ethics committees of the Korean Cancer Study Group, and all patients provided written informed consent. The Transparent Reporting of Evaluations With Nonrandomized Designs (TREND) reporting guidelines were followed.

### Study Procedures

Eligible patients received 6 cycles of neoadjuvant pertuzumab (840 mg at first cycle, followed by 420 mg administered intravenously), atezolizumab (1200 mg administered intravenously), docetaxel (75 mg/m^2^ administered intravenously), and trastuzumab (600 mg injected subcutaneously) every 3 weeks, followed by curative surgery. Tripegfilgrastim (6 mg injected subcutaneously) was administered 24 hours after each cycle at the physician’s discretion. After surgery, patients who achieved pCR were treated with 12 cycles of atezolizumab (1200 mg administered intravenously), trastuzumab (600 mg injected subcutaneously), and pertuzumab (420 mg administered intravenously) every 3 weeks. Fourteen cycles of trastuzumab emtansine (3.6 mg/kg administered intravenously) with atezolizumab (1200 mg administered intravenously) every 3 weeks were administered to patients who did not achieve pCR. However, the final decision regarding the adjuvant-targeted regimen was made at the physician’s discretion.

A cycle could be delayed up to 3 weeks to allow for sufficient recovery time. If treatment could not be started after the 3-week delay, the patients were removed from the study. Regarding docetaxel, occurrence of grade 3 or higher toxic effects or recurrence of grade 2 toxic effects led to 1 level of dose reduction (80% of the prior dose). Any patient who had required 1 dose reduction and experienced a toxic effect that would cause a second and third dose reduction was removed from the study. Regarding atezolizumab, trastuzumab, and pertuzumab, dose modification was not allowed; however, interruption was allowed in the case of treatment-related grade 3 or higher toxic effects. Atezolizumab was permanently discontinued when a patient could not recover from an atezolizumab-related toxic effect for more than 6 weeks.

Clinical tumor assessment was performed by the investigators with breast magnetic resonance imaging, breast ultrasonography, and/or computed tomography of the chest, abdomen, and pelvis. Objective response rate was evaluated according to the Response Evaluation Criteria in Solid Tumors, version 1.1. Toxic effects were assessed using the National Cancer Institute’s Common Terminology Criteria for Adverse Events, version 5.0.

Tumor tissue before systemic treatment from every participant and at surgery from all the patients without pCR were obtained for biomarker analysis. Optional additional tumor biopsy for biomarker analysis was performed at 3 weeks after the first cycle of neoadjuvant treatment. The programmed cell death 1 (PD-L1) status of the tumor was evaluated through immunohistochemistry using the Ventana PD-L1 (SP142) Assay (Roche Diagnostics) by a single pathologist (Y.L.C.) at the central laboratory. Programmed cell death 1 positivity was defined as immunoreactivity in immune cells in 1% or more of the tumor area.

### Outcomes

The primary end point of this study was pCR rate, which was defined as the absence of invasive cancer cells in the primary tumor and regional lymph nodes (ypT0/isN0). Pathologic complete response was assessed at microscopic examination following curative surgery by each local pathology department. Secondary end points were clinical objective response rate, 3-year event-free survival rate according to pCR achievement, disease-free survival, overall survival, toxic effects, and quality-of-life outcomes. Exploratory biomarker analyses, including PD-L1 expression, tumor mutational burden, immune signature profiling, and genomic profiling, were planned.

### Statistical Analysis

For the aim of this study, the sample size of 60 patients was set at the alternative hypothesis pCR rate of 65% or higher to test the null hypothesis pCR rate of 50% or lower under the 1-sided significance level 10% to get the power 80%. The study planned to recruit 67 patients, assuming a 10% dropout rate. Because all 67 patients were treated, the study treatment is rejected if pCR shows in 38 or fewer patients, with a power of 83%. Otherwise, the null hypothesis is rejected in favor of continuance to the next phase. The power and critical values of the sample sizes were computed using PASS 2022, version 22.0.2 (NCSS Statistical Software).

## Results

**Figure 1.**
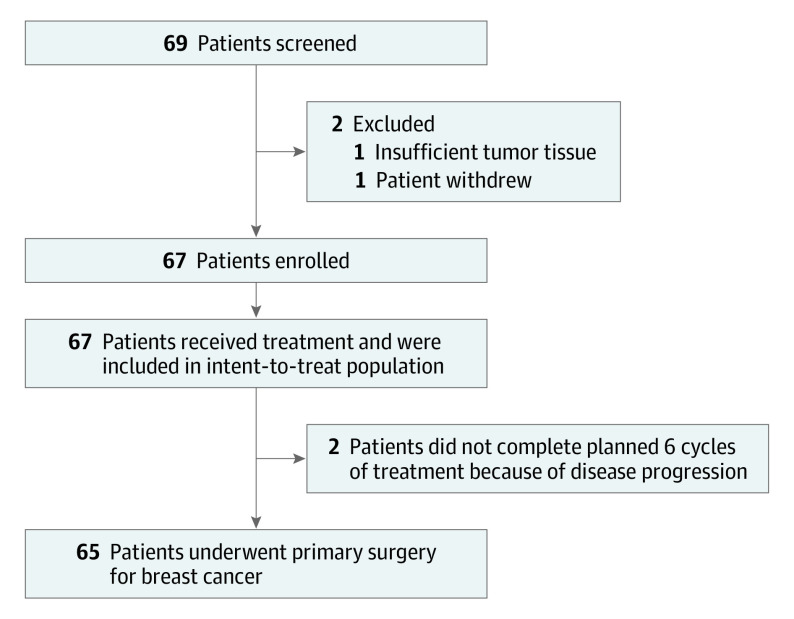
CONSORT Diagram

A total of 67 patients were enrolled from 6 institutions in Korea from May 2019 to May 2020. At data cutoff in February 2021, all patients had completed the neoadjuvant treatment. Two patients showed disease progression during the neoadjuvant phase, and the remaining 65 patients underwent curative surgery (breast-conserving surgery in 42 patients and total mastectomy in 23 patients) (Figure 1). R0 resection was achieved in 64 patients. Baseline characteristics of the enrolled patients are summarized in Table 1 and eAppendix in Supplement 2. The median (range) age of the patients was 52 (33-74) years. Hormone receptor expression analysis yielded positive results in 32 (48%) patients and negative results in 35 (52%) patients. Most of the enrolled patients had clinical stage II breast cancer (n = 49 [73%]). The PD-L1 expression status before systemic treatment was evaluated in 66 patients and was positive only in 13 (20%) patients.

**Table 1.** Patient Characteristics at Baseline (N = 67)

Characteristic	No. (%)
Median age (range), y	52 (33-74)
<50	24 (36)
50-59	29 (43)
≥60	14 (21)
Histologic findings	
Invasive ductal carcinoma	64 (96)
Other	3 (4)
HR expression	
HR positive	32 (48)
ER positive/PR positive	18 (27)
ER positive/PR negative	14 (21)
HR negative	35 (52)
*ERBB2 *status	
IHC score ≥3	59 (88)
IHC score ≥2 and ISH positive	8 (12)
ECOG performance status score	
0	49 (73)
1	17 (25)
Clinical tumor size	
cT1	3 (5)
cT2	45 (67)
cT3	19 (28)
Clinical node stage	
cN0	19 (28)
cN1	36 (54)
cN2	3 (5)
cN3	9 (13)
Clinical cancer stage	
IIA^a^	16 (24)
IIB	33 (49)
IIIA	9 (13)
IIIB	0
IIIC	9 (13)
Programmed cell death 1 expression^b^	
Negative	53 (80)
≤1%	5 (8)
>1% to <10%	4 (6)
≤10%	4 (6)

Abbreviations: ECOG, Eastern Cooperative Oncology Group; ER, estrogen receptor; HR, hormone receptor; IHC, immunohistochemistry; ISH, in situ hybridization; PR, progesterone receptor.

^a^ Among 16 patients with clinical stage IIA cancer, the number of patients with T2N0M0 was 13, and 11 of these patients had tumors 3 cm or smaller and N0.

^b^ Among 66 patients.

**Figure 2.**
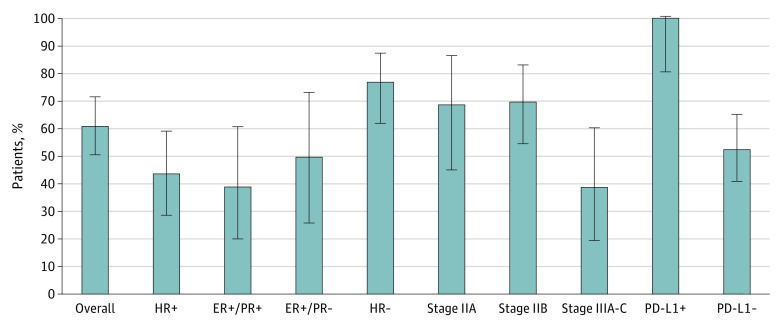
Pathologic Complete Response (pCR) Rate Overall and in Each Subgroup Among the 67 patients included, 41 (61%) achieved pCR. The pCR rate was higher in patients with hormone receptor–negative (HR−) subtype vs hormone receptor–positive (HR+) subtype (27 of 35 [77%] patients vs 14 of 32 [44%] patients), estrogen receptor–positive (ER+)/progesterone receptor–negative (PR−) cancer vs ER+/progesterone receptor–positive (PR+) cancer (7 of 14 [50%] patients vs 7 of 18 [39%] patients), stages IIA and IIB vs stage III cancer (11 of 16 [69%] patients and 23 of 33 [70%] patients vs 7 of 18 [39%] patients), and positive programmed cell death 1 (PD-L1+) expression vs negative programmed cell death 1 (PD-L1−) expression (13 of 13 [100%] patients vs 28 of 53 [53%] patients). Error bars indicate 95% CIs.

**Figure 3.**
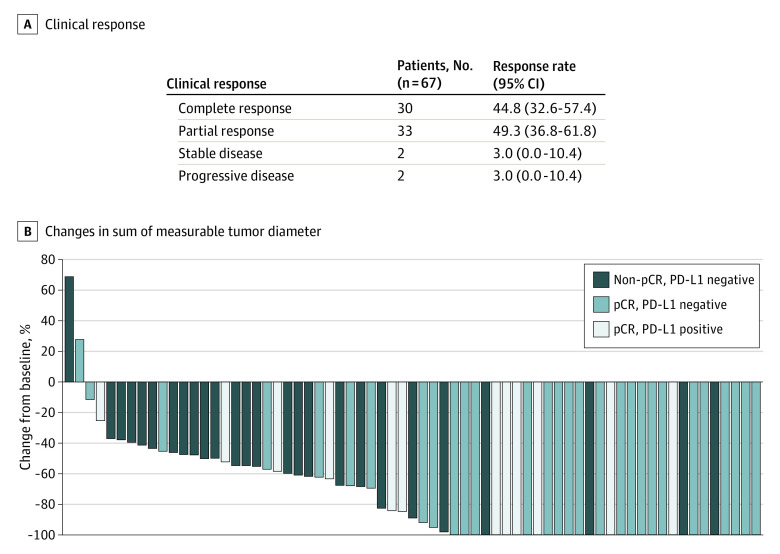
Objective Response Clinical response (A) and the greatest changes from baseline in sum of the longest diameters of measurable tumors among the 67 included patients (B). pCR indicates pathologic complete response; PD-L1, programmed cell death 1.

Pathologic complete response was achieved in 41 patients, and the overall pCR rate (ypT0/isN0) was 61% (90% CI, 50%-71%); 8 (12%), 13 (19%), and 3 (4%) patients had residual cancer burden class I, II, and III responses, respectively (eTable in Supplement 2). The pCR rate was higher in patients with hormone receptor–negative subtype vs hormone receptor–positive subtype (27 of 35 [77%] patients vs 14 of 32 [44%] patients), stages IIA and IIB vs stage III (11 of 16 [69%] patients and 23 of 33 [70%] patients vs 7 of 18 [39%] patients, respectively), and positive PD-L1 expression vs negative PD-L1 expression (13 of 13 [100%] patients vs 28 of 53 [53%] patients) (Figure 2). The clinical objective response rate was 94.0% (Figure 3A). The greatest changes in the sum of measurable tumor diameters are shown in Figure 3B.

Toxic effects experienced during the neoadjuvant PATH regimen are summarized in Table 2. The most common all-grade hematologic adverse event was neutropenia (n = 9 [13%]), of which most events were grade 3 or higher (n = 8 [12%]). Five (8%) patients experienced febrile neutropenia. The nonhematologic toxic effects included myalgia (n = 50 [75%]), alopecia (n = 45 [67%]), neuropathy (n = 39 [58%]), diarrhea (n = 34 [51%]), fatigue (n = 27 [40%]), nausea (n = 22 [33%]), and mucositis (n = 21 [31%]). However, grade 3 or higher nonhematologic toxic effects developed in only 5 (8%) patients. The most common immune-related adverse event was skin rash (n = 43 [64%]), followed by fever (n = 20 [30%]), thyroid dysfunction (n = 7 [10%]), pneumonitis (n = 6 [9%]), hepatitis (n = 2 [3%]), and encephalitis (n = 1 [2%]). Grade 3 or higher immune-related adverse events developed in only 4 (6%) patients, including 1 case each of grade 3 rash, grade 3 fever, grade 3 hepatitis, and grade 3 encephalitis. The mean delivered dose of docetaxel was 71.6 mg/m^2^/cycle (95.5% of the planned dose). In 16 (24%) patients, the docetaxel dose was modified because of toxic effects. Treatment-related discontinuation or interruption of atezolizumab during neoadjuvant treatment occurred in 7 (10%) patients. No interruptions occurred in pertuzumab or trastuzumab treatment. Serious adverse events occurred in 14 (21%) patients. The most common serious adverse event was febrile neutropenia (n = 4 [6%]), followed by fever (n = 3 [5%]) and other immune-related adverse events (n = 2 [3%]). No treatment-related death occurred during the neoadjuvant phase in this study.

**Table 2.** Overall Safety in the Neoadjuvant Phase (N = 67)

Adverse events	Patients, No. (%)	
	All adverse event grades	Grade 3/4 adverse events
Total	66 (99)	21 (31)
Hematologic		
Neutropenia	9 (13)	8 (12)
Febrile neutropenia	5 (8)	5 (8)
Anemia	4 (6)	0
Thrombocytopenia	4 (6)	0
Nonhematologic		
Myalgia	50 (75)	0
Alopecia	45 (67)	0
Neuropathy	39 (58)	1 (2)
Diarrhea	34 (51)	0
Fatigue	27 (40)	0
Nausea	22 (33)	1 (2)
Mucositis	21 (31)	0
Edema	11 (16)	0
Constipation	9 (13)	0
Hand-foot syndrome	7 (10)	0
AST elevation	6 (9)	0
ALT elevation	8 (12)	2 (3)
Vomiting	5 (8)	0
Infusion-related reaction	3 (5)	0
Pneumonia	1 (2)	1 (2)
Immune related		
Rash	43 (64)	1 (2)
Fever	20 (30)	1 (2)
Thyroid dysfunction	7 (10)	0
Pneumonitis	6 (9)	0
Hepatitis	2 (3)	1 (2)
Encephalitis	1 (2)	1 (2)
Serious adverse events	14 (21)	NA
Leading to hospital admission^a^	13 (19)	NA
Febrile neutropenia	4 (6)	NA
Fever	3 (5)	NA
Immune related	2 (3)	NA
Adverse event leading to treatment delay^b^	7 (11)	NA
Adverse event leading to docetaxel dose reduction^c^	16 (24)	NA
Adverse event leading to atezolizumab withdrawal or interruption^d^	7 (11)	NA

Abbreviations: ALT, alanine aminotransferase; AST, aspartate aminotransferase; NA, not applicable.

^a^ Other serious adverse events leading to hospital admission were grade 3 ALT elevation (n = 1), grade 3 pneumonia (n = 1), grade 3 back pain (n = 1), grade 2 nausea (n = 1), and grade 2 gastric ulcer (n = 1).

^b^ Includes liver enzyme elevation or hepatitis (n = 4), grade 3 febrile neutropenia (n = 1), grade 2 neutropenia (n = 1), and surgery for appendix mucocele (n = 1).

^c^ Includes grade 3 or higher neutropenia (n = 6), grade 2 liver enzyme elevation (n = 2), grade 3 anemia (n = 1), grade 3 pneumonia (n = 1), grade 3 sensory neuropathy (n = 1), grade 2 rash (n = 1), grade 2 edema (n = 1), grade 2 diarrhea (n = 1), grade 1 epigastric pain (n = 1), grade 2 fever, and grade 2 nausea (n = 1).

^d^ Toxic effects leading to the discontinuation of atezolizumab were grade 3 hepatitis (n = 2), grade 3 encephalitis (n = 1), grade 3 skin rash (n = 1), and grade 2 diarrhea (n = 1). Atezolizumab treatment was interrupted in 2 patients with liver enzyme elevation.

## Discussion

The neoadjuvant atezolizumab combination PATH demonstrated a pCR rate worth further investigation, with fewer hematologic toxic effects with prevalent long-acting filgrastim support. Only 16% of the participants experienced dose reduction of docetaxel, while 40% of patients receiving neoadjuvant TCHP experienced dose modification owing to adverse events in a large, real-world cohort of Korean patients.^9^

Clinical evidence supporting a role of immunotherapy combinations in *ERBB2*-positive breast cancer are limited. The KATE2 trial found no benefit of atezolizumab addition in the intent-to-treat population.^10^ The IMpassion050 trial found no increase in pCR in the intent-to-treat and PD-L1–positive populations.^11^ The observed discrepancy among efficacy of immunotherapy may be attributed to the difference in antitumor immunity associated with the breast cancer subtype, disease burden, or the partner regimen. In the present study, the pCR rate was higher in the hormone receptor–negative subgroup, in patients with lower tumor burden, and in patients with positive PD-L1 expression at baseline. The better benefit of immunotherapy in a PD-L1–positive population was also suggested in previous studies for metastatic *ERBB2*-positive breast cancer.^10,12^ The pCR rate of the neoadjuvant taxane-trastuzumab-pertuzumab triplet regimen was 49% in the NeoSphere study^6^ and 55% in the DAPHNE study,^13^ which suggests careful patient selection is needed when giving intensified or deintensified treatment. This study suggests a possible role of PD-L1 expression in patient selection for novel deintensified immunotherapy combination in *ERBB2*-positive early breast cancer in the future.

### Limitations

This was a small-sized, single-arm study; therefore, any confirmatory conclusion cannot be drawn. Although pCR is a validated surrogate marker for long-term event-free survival in breast cancer neoadjuvant trials, it is not yet confirmed in the case of immunotherapy. Recent long-term outcomes of the GeparNUEVO^14^ and KEYNOTE-522^15^ trials have shown that magnitude of long-term survival benefit may be larger than benefit in pCR rate with neoadjuvant immunotherapy. For the present study, the adjuvant phase is currently ongoing and long-term event-free survival will be determined in the future.

## Conclusions

Results of the Neo-PATH nonrandomized clinical trial suggest that the pCR rate of the neoadjuvant pertuzumab, atezolizumab, docetaxel, and trastuzumab combination warrants continuation to the next phase. These preliminary results should be further investigated in a large-scale randomized clinical trial.

## Notes

Supplement 1. Trial Protocol

Supplement 2.
eAppendix
**eTable.** Residual tumor burden in patients with non-pathological complete response

Supplement 3. Data Sharing Statement
