# Supplementary material for: Response Rate and Safety of a Neoadjuvant Pertuzumab, Atezolizumab, Docetaxel, and Trastuzumab Regimen for Patients With ERBB2-Positive Stage II/III Breast Cancer: The Neo-PATH Phase 2 Nonrandomized Clinical Trial
Source: JAMA Oncol. 2022 Jul 7;8(9):1271–7. doi: 10.1001/jamaoncol.2022.2310 (PMC10881214; doi:10.1001/jamaoncol.2022.2310)
Supplement: Supplement 1. — Trial Protocol [file jamaoncol-e222310-s001.pdf]

Protocol Title :

Phase Ib-II combination Neoadjuvant Chemotherapy with Docetaxel plus Atezolizumab plus Herceptin SC and Pertuzumab (TAHP) for Patients with HER2-positive Early Breast Cancer and Subsequent Atezolizumab plus Herceptin SC and Pertuzumab (AHP) Adjuvant tHerapy after Surgery (Neo-PATH)

**PROTOCOL NUMBER**	Neo PATH
**VERSION**	2.3
**DATE**	2020.05.14
**PRINCIPLE INVESTIGATOR**	YEON HEE PARK, MD, PhD<br>Samsung Medical Center

REVISION HISTORY

Revision number	Effective Date	Description of revision and reason
Ver 1.0	2018.12.13	First version
Ver 1.1	2019.02.21	Change in background according to IRB's recommendation, Change in safety monitoring tests according to MFDS's recommendation, Addition of appendix 4 for atezolizumab related adverse event management guideline according to MFDS's recommendation, Clarification and change in timing of obtaining tumor tissue, Addition of the name of laboratory where tumor tissue are analyzed, Addition of confidentiality, Change in reporting process of serious adverse events, Addition of references, etc.
Ver 1.2	2019.02.28	Clarification of primary endpoint, hypothesis, and sample size calculation, etc.
Ver 1.3	2019.03.06	Change in timing of obtaining tissue and blood for exploratory analysis, Change in the process of serious adverse events, Addition of atezolizumab related adverse event of special interest, Change in atezolizumab related adverse event management guideline according to investigational brochure update, etc.
Ver 1.4	2019.03.22	Change in timing of optional re-biopsy tumor tissue, Correction of typos, etc.
Ver 1.5	2019.05.15	Clarification of IP dose (loading dose of pertuzumab), Change in the amount and timing of blood sample, Change in the process of serious adverse events.
Ver 1.6	2019.05.28	Addition of reason for reloading of pertuzumab, Clarification of treatment cycles, Clarification of survival follow up start time, Change of serious adverse event reporting method, Addition of congestive heart failure as trastuzumab related adverse event of special interest, Update of atezolizumab related adverse events of special interest, Correction of typos, etc.
Ver 1.7	2019.06.17	Correction of typos in IP dose modification section.
Ver 1.9	2019.08.08	Change of eligibility and exclusion criteria according to Roche's recommendation, Addition of contraindicated concomitant

		medications (live attenuated vaccine and systemic immune modulators, systemic immunosuppressants), Addition of laboratory tests including coagulation test and endocrine related test, Addition of immune related nephritis and myositis in appendix 4, etc.
Ver 2.0	2019.09.06	Change of eligibility (age) and exclusion criteria according to adjuvant trastuzumab emtansine treatment in patients with non-pCR, **Change of adjuvant treatment in patients with non-pCR, from doxorubicin and cyclophosphmiade to trastuzumab emtansine according to Katherine phase 3 trial results**, Addition of tripegfilgrastim for primary prophylaxis of neutropenic fever during neoadjuvant phase, Clarification of statistical analysis plan, Addition of trastuzumab emtansine related adverse event guideline, etc.
`Ver 2.1	2020.02.04	Change of background, Clarification of eligibility and exclusion criteria, Extension of enrolment period and study duration, Clarification of study procedure, etc.
Ver 2.2	2020.02.20	Change of number of cycles of adjuvant treatment of patients with non-pCR, from 12 cycles to 14 cycles, Clarification of dose dose modification and discontinuation guideline, etc.
Ver 2.3	2020.05.14	Clarification of duration of collecting concomitant medications, Change of tumor assessment window after primary surgery, Adding immune related myocarditis as atezolizumab related adverse event of special interest, Change of guideline for atezolizumab related infusion related reaction according to change of atezolizumab IB ver.15, etc.

Table of Contents

1. Protocol Title 14
2. Introduction and Background 14
2.1 Rationale of neoadjuvant chemotherapy 14
2.2 Treatment of early HER2-positive breast cancer 14
2.3 Neoadjuvant Chemotherapy in HER2-Positive Breast Cancer 16
2.4 Efficacy and safety data of atezolizumab in HER2 positive breast cancer patients 18
2.4 Study rationale 22
3. Study Objectives and Endpoints 25
3.1 Primary objective: 25
3.2 Secondary objectives: 25
4. Study treatment 26
4.1 Investigational medicinal products 26
4.1.1 Docetaxel 26
4.1.2 Atezolizumab 26
4.1.3 Herceptin sc 27
4.1.4 Pertuzumab 27
4.1.5 Trastuzumab Emtansine 27
5. Concomitant Therapy 26
5.1 Prohibited Therapy 26
5.2 Permitted Therapy 26
6. Study population 27
7. Patients 27
7.1 Inclusion Criteria 27
7.2 Exclusion Criteria 28
7.3 Determination of sample size 30
7.3.1 Sample size 30
7.3.2 Determine of sample size 30
7.4 Stratification factors 31
8. Duration of study 31
9. Study design 31

10. Schedules of assessments and procedures 34
10.1 Experimental group 34
10.2 List of evaluation 41
10.2.1 Screening 41
10.2.2 Drug administration (Visit window ±5 days) and EOT visit Weight 43
10.2.3 Follow up visit 45
11. Dose modification 45
11.1 Docetaxel 45
11.2 Atezolizumab 45
11.3 Herceptin sc and pertuzumab 46
11.4 Trastuzumab Emtansine 47
12. Statistical analysis 47
12.1 Primary and secondary endpoints 47
12.1.1 Primary endpoint 47
12.1.2 Secondary endpoint 48
12.2 Definition of Analysis Populations 49
12.3 Efficacy and safety analysis 49
12.3.1 Primary End point: pathologic complete response 50
12.3.2 Event free survival and overall survival 50
12.3.3 Response evaluation and toxicity evaluation 50
12.3.4 Quality of Life 51
13. Biomarker analysis 51
13.1 Exploratory biomarker analysis using tissue blocks 51
13.2 Exploratory biomarker analysis using blood 51
13.3 Exploratory biomarker analysis using surgical tissues 52
14. Definition and Reporting of Adverse Events 52
14.1 Definition of adverse events 52
14.1.1 Adverse events 52
14.3 Serious Adverse Events 53
14.3.1 Serious Adverse Event Reporting 54
14.3.2 Suspected Unexpected Serious Adverse Reaction Report 55
14.3.3 Reporting of study specific AEs and SAEs 55
14.4 Drug-adverse Event Relationship 57

15. Withdrawal from the study........58
15.1 Study Discontinuation........58
15.2 Patient withdrawal from study........58
15.3 Subject replacement........58
16. Emergent process........58
17. Ethics and general study administration........58
17.1 Local regulations/ Declaration of helsinki........58
17.2 Informed consent........59
17.3 Confidentiality of trial documents and patient records........59
17.4 Conditions for modifying the protocol........59
17.5 Monitoring........60
17.6 Criteria for Premature Withdrawal........60
17.7 Investigator's Files / Retention of Documents........61
18. References........63
19. Appendicies........65
19.1 Appendix 1. Performance Status Criteria........65
19.2 Appendix 2. RECIST 1.1 Criteria for Response........65
19.3 Appendix 3. CTCAE(Common Terminology Criteria for Adverse Events v5.0)........67
19.4 Appendix 4 Risk associated with atezolizumab and guidelines for management of adverse events associated with atezolizumab........68
19.5 Appendix 5. Pertuzumab and Trastuzumab related toxicity management........88
19.6 Table 15. Guidelines for Management of Adverse Events Associated with Trastuzumab Emtansine........89
19.7 Guidelines for Management of Adverse Events that are Potential Overlapping Toxicities Associated with Trastuzumab Emtansine in Combination with Atezolizumab........92

<table>
<tr><th colspan="3">TABULATED SYNOPSIS</th></tr>
<tr><td>Title</td><td colspan="2">Phase Ib-II combination Neoadjuvant Chemotherapy with Docetaxel plus Atezolizumab plus Herceptin SC and Pertuzumab (TAHP) for Patients with HER2-positive Early Breast Cancer (EBC) and Subsequent Atezolizumab plus Herceptin SC and Pertuzumab (AHP) Adjuvant therapy after Surgery</td></tr>
<tr><td>Study duration</td><td colspan="2">2019.05 – 2023.04 (48 months)</td></tr>
<tr><td>Study chair/ Institution</td><td colspan="2">Yeon Hee Park, M.D., Ph.D
Samsung Medical Center, Sungkyunkwan University School of Medicine</td></tr>
<tr><td>Study Sub-investigators</td><td colspan="2">Korean Cancer Study Group affiliating Institutes</td></tr>
<tr><td>Study method</td><td colspan="2">A Phase Ib-II</td></tr>
<tr><td rowspan="9">Study Objectives and Corresponding Endpoints</td><td>Objectives</td><td>Corresponding Endpoints</td></tr>
<tr><td colspan="2">Primary Objective:</td></tr>
<tr><td>To evaluate the pCR rate of TAHP neoadjuvant chemotherapy with the patients with HER2+ EBC</td><td>Pathologic complete response rate (pCR)</td></tr>
<tr><td colspan="2">Secondary Objective:</td></tr>
<tr><td>➢ To evaluate the EFS of atezolizumab add-on adjuvant treatments with Herceptin SC plus pertuzumab in patients with Breast Cancer of patients with pCR versus non-pCR
➢ To assess the efficacy of atezolizumab add-on adjuvant treatments according to PD-L1 status by IHC(IC0 and IC1/2/3 subgroup analysis)</td><td>➢ 3-year Event free survival (EFS) of the patients with pCR vs. non-pCR to TAHP
➢ Overall survival (OS)
➢ Disease Free Survival(DFS)
➢ pCR and EFS based on PD-L1 status
➢ QOL</td></tr>
<tr><td colspan="2">Safety Objectives</td></tr>
<tr><td>To determine the safety and tolerability of atezolizumab add-on adjuvant treatments with Herceptin SC plus pertuzumab for remaining 1 year in patients with Breast Cancer</td><td>Safety and Toxicity
Incidence and severity of adverse events, AESIs and SAEs with severity determined according to NCI CTCAE v5.</td></tr>
<tr><td colspan="2">Exploratory Biomarker Objectives:</td></tr>
<tr><td>1. To evaluate efficacy of Neo-adjuvant TAHP chemotherapy based on HER2 amplification (level: high-low)</td><td>➢ Multi-omic profiling using F1CDx and WTS platform in house
➢ Immune signatures such as PD-L1</td></tr>
</table>

	2. To assess the role of PI3K on resistance of HER2 positive breast cancer patients<br>3. To evaluate correlation between TMB and clinical outcome or any genetic environmental change to response to.	expression, tumoral mutation burden (TMB), Intrinsic subtyping, mutation signature, pathway analysis including PIK3CA and HER<br>➢ Single cell sequencing of residual cancer after neoadjuvant PATH<br>➢ Cf DNA profiling
**Study Rationale**	Immunogenic potential related with HER2-directed therapy has been reported and it would be one of candidate for combination partner with immune-check point therapeutics.(John et al. 2011) Maybe, overcoming resistance to HER2-directed therapy could be an option for other immune-oncologic agents. Hence, characterization of tumor-associated and systemic immune responses to immunotherapy with anti HER2+ and chemotherapies may help us evaluate the applicability of immune therapies and provide rational designs of combination therapies with chemotherapies in these HER2+ BC patients in need of effective treatment, especially for patients with EBC with neoadjuvant aim.<br>Given the data with our experience and the other reports (NEOSPHERE: Gianni L, et al. Lancet Oncol 2012; 13:25–32), general treatment outcome from tratuzumab plus pertuzumab is about 50% and we assume this as H0 assumption. The most favorable regimen for this setting is TCHP (docetaxel plus carboplatin plus trastuzumab plus pertuzumab), which showed remarkable pCR rate of approximately 60% [RWD is warranted: Total pCR rate: 65% (pCR rate: ER- , beyond 80%; ER+, about 50%), unpublished local source of SMC data which is shown pCR rate with TCHP treatment below)]. However, toxicities of the regimen are pretty serious; myelosuppression including neutropenic fever (NF), neurotoxicity, nephrotoxicity, emesis and etc. Thus, the patients who have co-morbidities and old ages are difficult to treat TCHP regimen considering comprehensive benefit/risk, and there are still drug-related serious events associated with serious toxicities such as grade 3 or higher grade of diarrhea, hematochezia, and sepsis. If atezolizumab enhances combination chemotherapy with taxane plus HER2-doublet, this agent could replace carboplatin as well as mitigating serious toxicities of carboplatin (anemia, neutropenic fever, thrombocytopenia, grade 2 or more neurotoxicity, nephrotoxicity, and emesis) as well as improving QoL. This enriched population may have main microenvironmental characteristics to define role of	

	immunogenicity through multi-omic profiling. In addition, relationship between cross-talk pathways will be revealed including PI3K pathway, which is considered as one of main resistance mechanism to HER2-directed therapy.<br>Biomarker development for adapting immune-oncologic agents is underway including immunohistochemistry including PD1 and PD-L1 expression, mutational loads, and other genes expression profiling. For patients with primary refractory HER2+ EBC, there is un-met medical need to select right patients for right drugs add on atezolizumab for their adjuvant treatments together with trastuzumab and pertuzumab.<br>Recently, introduction of gene expression profiling (GEP) has brought much potential to expand immune-oncologic agents with or without combination of the other conventional treatments including HER2-directed agents to overcoming resistance to HER2 pathway especially for patients with HER2+ BCs as well as TNBCs.<br>We've already established genomic platform for BC patients. Using this genomic platform and experience, I'd like to propose IO and mutational profiling for HER2+ EBC patients. We are to better define those HER2-positive BCs who may benefit from add on atezolizumab to trastuzumab through multi-omic profiling using Foundation One.
Subject selection	**1. Eligibility criteria:**<br>1) Patients with locally advanced, inflammatory or early stage of histologically confirmed diagnosis of breast cancer<br>2) Primary breast tumor site of >2 or nodal involvement was confirmed at the same side of breast by fine needle aspiration (determined by AJCC 7th edition, Clinical stage IIA-IIIC)<br>3) Eastern Cooperative Oncology Group Performance of 0-1<br>4) Patient has HER2-positive breast cancer as 3+ by IHC or gene amplified by in situ hybridization (ISH) as defined by a ratio ≥ 2.0<br>5) Available to provide tumor sample<br>6) LVEF ≥55% at baseline by ECHO or MUGA<br>7) Patient is ≥ 19 years old<br>8) Patient has no possibility of pregnancy or Urine or blood b-HCG result is negative.<br>9) Patient has adequate bone marrow function (ANC ≥ 1,500/ul, Platelet ≥100,000/ul, Hemoglobin ≥ 9.0 g/dl)<br>10) Patient who are not receiving therapeutic anticoagulant: INR or a PTT ≤ 1.5* ULN. Patient who are receiving therapeutic anticoagulant need to on a stable anticoagulant regimen.

11) Patient has adequate liver function (Serum bilirubin ≤1.5* upper limit of normal and AST/ALT 1.5*upper limit of normal)
12) Patient agrees to use effective contraception or patient has no possibility of pregnancy
13) To obtain written informed consent
14) To agree tissue and blood collection for biomarker evaluation

2. Exclusion criteria

1) Patients who have metastatic disease (Stage IV)
2) Tumor size less than 2cm and N0
3) History of secondary malignancy (except in situ carcinoma of the cervix, adequately treated non-melanoma skin cancer, thyroid papillary carcinoma or malignancy history which is over 5 years after treatment completion without relapse)
4) Any previous treatment against including chemo, hormonal therapy
5) Administration of a live, attenuated vaccine within 4 weeks before Day 1 or anticipation that such a live attenuated vaccine will be required during the study and 5 months after last dose of atezolizumab
6) Severe infections within 4 weeks prior to Day 1, including, but not limited to, hospitalization for complications of infection, bacteremia, or severe pneumonia. Signs or symptoms of significant infection within 2 weeks prior to Day 1
7) Received oral or IV antibiotics within 2 weeks prior to Cycle 1 Day 1
8) Active tuberculosis or pneumonia
9) History of idiopathic pulmonary fibrosis
10) Known clinically significant liver disease, including alcoholic, hepatitis B or C infection
    A. Patients with past or resolved hepatitis B infection (defined as having a negative HBsAg test or a positive hepatitis B core antigen [anti-HBc] test confirming HBV DNA negative results) are eligible.
    B. Patients positive for hepatitis C virus (HCV) antibody are eligible only if polymerase chain reaction assay (PCR) is negative for HCV RNA
    C. Known clinically treatment required liver disease, including cirrhosis, inherited liver disease and idiopathic hepatitis
11) Inadequate kindny function such as serum creatinine ≥ 1.5 * Upper Limit of Normal or Serum Creatinine is within 1.0-1.5 * Upper Limit of Normal and eGFR < 60 mL/min/1.73m^2
12) Uncontrolled hypertension (systolic blood pressure >180mmHg and/or diastolic blood pressure >100mmHg), Unstable Angina Pectoris, Congestive Heart Failure,

	significant arrhythmias (i.e atrial fibrillation, paroxysmal supraventricular tachycardia), Myocardiac infarction or LVEF < 55% within 6 months prior to randomization<br>13) Total bili >1.5 ULN (except for Gilbert's syndrome), AST/ALT > 1.5 ULN, ALP > 2.5 ULN<br>14) Immunosuppression or known HIV infection<br>15) Dyspnea at rest which is required for continuous oxygen therapy or other lung disease<br>16) Uncontrolled serious infectious disease<br>17) Active heart disease such as myocardiac infarction, angina pectoris, uncontrolled arrhythmias within 6 months and uncontrolled severe medical, psychiatric disease<br>18) Patients with prior allogeneic stem cell or solid organ transplantation<br>19) Pregnant or lactating or intending to become pregnant during or within 7 months after the last dose of study treatment<br>20) History of hypersensitivity for IMP or excipient<br>21) History of autoimmune disease including, but not limited to, systemic lupus erythematosus, rheumatoid arthritis, inflammatory bowel disease, vascular thrombosis associated with antiphospholipid syndrome, Wegener's granulomatosis, Sjögren's syndrome, Bell's palsy, Guillain-Barré syndrome, multiple sclerosis, vasculitis, or glomerulonephritis<br>22) Patients who are not available to provide tumor tissue<br>22) Patient who can't comply with the protocol and who is not willing to comply with the protocol<br><br>Exclusions Related to Trastuzumab Emtansine in the Adjuvant Setting:<br>• Patients who achieved pCR defined as the absence of residual invasive cancer on hematoxylin and eosin evaluation of the complete resected breast specimen and all sampled regional lymph nodes following completion of neoadjuvant systemic therapy (i.e., ypT0/is ypN0 in the current AJCC staging system, 8th edition)<br>• Evidence of clinically evident gross residual or recurrent disease following neoadjuvant therapy and surgery<br>• Unable to complete surgery with curative intent after conclusion of neoadjuvant systemic therapy<br>• Patient discontinued treatment with trastuzumab because of toxicity during the neoadjuvant phase of the study<br>• Clinically significant history of liver disease, including cirrhosis, current alcohol abuse, autoimmune hepatic disorders, or sclerosis cholangitis<br>• Patients with Grade $\geq$2 peripheral neuropathy<br>• Prior treatment with trastuzumab emtansine<br>• Serum AST, ALT, and alkaline phosphatase not within $\leq 1.5 \times$ ULN<br>• Serum total bilirubin not within normal range ($\leq 1.0 \times$ ULN)<br>Except for patients with Gilbert's syndrome, for whom direct bilirubin should be within the normal range

	• Serum creatinine not within <1.5 x ULN
Treatment plan	**Drug administration**<br>**(A, Neoadjuvant setting):** Docetaxel (75mg/m2, intravenous(IV)) Day(D)1, Atezolizumab (1200mg, IV) D1, Trastuzumab (600mg subcutaneous(SC))D1 and Pertuzumab (840mg loading dose at Cycle 1 followed by 420mg(IV))D1 X 6 cycles q3weeks, intravenous(IV) administration<br>**(B, Adjuvant setting) :**<br>**patients with pCR:** Atezolizumab (1200mg, IV), Trastuzumab (600mg, SC) and Pertuzumab (840mg loading dose at Cycle 1 followed by 420mg, IV – reloading is required in case the infusion interval exceeds 6 weeks) D1 X 12 cycles q3weeks<br>**Patients with non-pCR:** Atezolizumab (1200mg, IV) and Trastuzumab emtansine (3.6mg/kg, IV) D1 X 14 cycles q3weeks<br>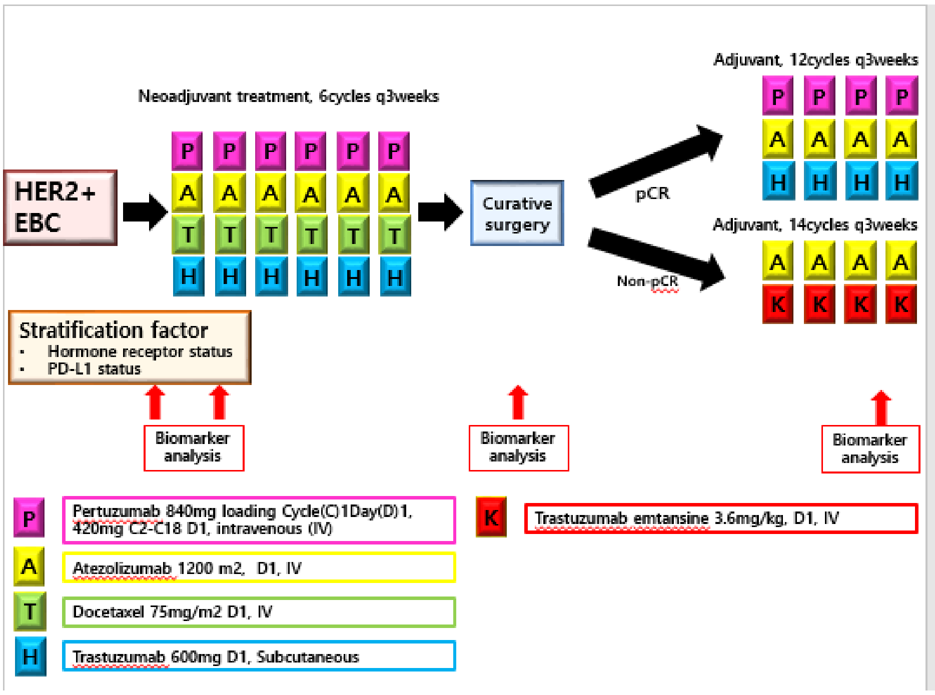
Statistical Consideration	Study primary endpoint is to investigate the pathologic complete response rate (pCR rate) of docetaxel + Herceptin sc + pertuzumab in combination with atezolizumab in HER2-positive breast cancer patients as neoadjuvant chemotherapy compared with docetaxel plus carboplatin plus trastuzumab plus pertuzumab (65% complete remission).<br>Based on Simon's optimal design (H0=50% vs. H1=65%), significant level 10%, power 80%<br>Step 1: If 23 patients were recruited and fewer than 12 patients had pathologic complete

	remission among the 23 patients, the study drug was rejected and the study was terminated.<br>Step 2: If more than 13 patients were diagnosed with pathologic complete remission, 37 patients were recruited (final 60 patients). If the number of patients who achieved pathologic complete remission was less than 34, the study drug was rejected.<br>A total of 67 patients will be recruited considering the dropout rate of 10%. The total study period including the patient recruitment period of 12 months and the follow - up period of 36 months will be 48 months.
Biomarker analysis	**Exploratory biomarker analysis using tissue blocks**<br>Biomarker studies using tissue blocks will be performed 1) at the time of diagnosis, 2) at 3weeks after 1st treatment (optional), and 3) at the time of surgery (non-pCR) 4) at progression after surgery (optional).<br>Genome analysis using the F1CDx panel, whole-exome sequencing and RNASeq will be performed. In addition, HER2 status, PD-1 and PD-L1 status will be assessed via immunochemical staining.<br><br>**Exploratory biomarker analysis using blood**<br>Biomarker studies using blood will be done 1) at the time of diagnosis, 2) at 3 weeks after first chemotherapy, 3) at the time of surgery and 4) at progression after surgery.<br>At the time of diagnosis, whole exome sequencing and circulating tumor DNA analysis will be performed using blood. In addition, circulating tumor DNA analysis will be performed after 3 weeks of first chemotherapy, at the time of surgery and after disease progression.<br><br>**Exploratory biomarker analysis using surgical tissues**<br>We performed single cell sequencing of patients with residual tumors who did not have complete pathologic remission at the time of surgery.

1. Protocol Title

Phase Ib-II combination Neoadjuvant Chemotherapy with Docetaxel plus Atezolizumab plus Herceptin SC and Pertuzumab (TAHP) for Patients with HER2-positive Early Breast Cancer and Subsequent Atezolizumab plus Herceptin SC and Pertuzumab (AHP) Adjuvant tHerapy after Surgery (Neo-PATH)

2. Introduction and Background

2.1 Rationale of neoadjuvant chemotherapy

The goals of neoadjuvant chemotherapy (NAC) are 1) to reduce the size of localized advanced breast cancer that cannot be operated on, and to make it possible to curative surgery and 2) when the operation is first performed, the size of breast cancer is reduced in patients who need breast conserving surgery and breast reconstruction. Moreover, in the clinical stage II / III of breast cancer, neoadjuvant chemotherapy inhibits micrometastasis from disease and effectively reduces tumor recurrence. Therefore, NAC improved the patients' survival and quality of life. Pathologic complete response (pCR) is the most important indicator of the efficacy of preoperative chemotherapy, therefore it has been used as the surrogating clinical marker of long-term survival. .[1]

In particular, according to the breast cancer subtype, the relationship between pCR and survival time is clearer in the case of breast cancer that has a rapid growing subtypes, such as HER2-positive breast cancer or triple negative breast cancer. Therefore, in these subtypes, higher pCR rate indicated lower BC recurrence and higher survival rate.[2]

2.2 Treatment of early HER2-positive breast cancer

The treatment and prognosis of patients with HER2-positive breast cancer have been transformed by the advent of HER2-targeted agents. The humanized monoclonal antibody trastuzumab (Herceptin), which binds to the extracellular domain of HER2, is approved for use as a single agent or in combination with chemotherapy or hormonal therapy in the metastatic setting, and as (neo)adjuvant treatment for patients with early- stage HER2-positive breast cancer. Globally, trastuzumab-based therapy is the recommended treatment for patients with HER2-positive early-stage breast cancer who do not have contraindications for its use (Herceptin Prescribing Information.[8,9,10]

An improvement to standard trastuzumab therapy for HER2-positive EBC comes with the addition of another HER2-targeted therapy, pertuzumab (Perjeta), approved in many countries for the treatment of HER2-positive metastatic breast cancer and for neoadjuvant use in patients with high-risk, HER2-positive EBC. This combination has been adopted in global guidelines for use in these patients in the neoadjuvant setting (Perjeta Prescribing Information.[8,9,10] Pertuzumab has also been recently approved in the United States, Europe, and other countries throughout the world for post-surgery (adjuvant) treatment of HER2-positive EBC at high risk of recurrence (Perjeta Prescribing Information.[9,10]
In HER2-positive EBC, neoadjuvant pertuzumab+trastuzumab+docetaxel provides a statistically significant and clinically meaningful improvement in pathological complete response (pCR) rates over trastuzumab+docetaxel alone (45.8% vs. 29.0%; p=0.0141).[12] Patients in this study went on to receive additional chemotherapy following surgery regardless of pCR status. In a descriptive analysis, 5-year DFS rates were reported to be higher in those patients who received neoadjuvant pertuzumab+trastuzumab+docetaxel than those patients who received trastuzumab+docetaxel alone (hazard ratio [HR]=0.60; 0.28-1.27). In a study in which neoadjuvant pertuzumab and trastuzumab were evaluated with either anthracycline- based chemotherapy or carboplatin-based chemotherapy, pCR rates (ypT0/is, ypN0 in the current American Joint Committee on Cancer [AJCC] staging system) for all regimens ranged from 57.3%-66.2%, with the highest pCR rate being achieved in the docetaxel/carboplatin/trastuzumab/pertuzumab regimen.[13]
Trastuzumab emtansine is an approved treatment for patients with HER2-positive advanced breast cancer who have received therapy with trastuzumab and a taxane and who have received prior therapy for metastatic disease or developed disease recurrence during or within 6 months of completing adjuvant therapy (Verma et al 2012; NCCN 2019). Study BO27938 (Katherine) was designed to investigate the effect of trastuzumab emtansine in patients with HER2-positive early breast cancer. The study was a randomized, multicentre, open-label Phase III study to evaluate the efficacy and safety of trastuzumab emtansine compared with trastuzumab as adjuvant therapy for patients with HER2-positive early breast cancer who have residual tumor present in the breast or axillary lymph nodes following neoadjuvant therapy containing a taxane and trastuzumab. Results showed that invasive DFS was significantly higher with trastuzumab emtansine than with trastuzumab (HR = 0.50; 95% CI, 0.39–0.64;

p < 0.001) (von Minckwitz et al 2019). The estimated percentage of patients free of invasive disease at 3 years from randomization was 88.3% with trastuzumab emtansine and 77.0% with trastuzumab. A higher proportion of patients receiving trastuzumab emtansine compared with patients receiving trastuzumab experienced Grade ≥ 3 adverse events (25.7% vs. 15.4%), serious adverse events (12.7% vs. 8.1%), and adverse events leading to discontinuation of study drug (18.0% vs. 2.1%). Recently, based on the results of the Katherine study, the U.S. Food and Drug Administration (FDA) approved trastuzumab emtansine in the adjuvant setting for patients with HER2-positive early breast cancer who have residual invasive disease after neoadjuvant taxane and trastuzumab-based treatment.

2.3 Neoadjuvant Chemotherapy in HER2-Positive Breast Cancer

1) TRYPHAENA[3]

The TRYPHAENA study was a global, multicenter randomized phase II trial of 223 HER2-positive, locally advanced breast cancer patients. The objectives of this trial were safety profiles and efficacy (pathologic complete remission (pCR) rate). As a result of this trial, the proportion of patients who achieved pathologic complete remission (pCR) was 66.2% in TCHP group (Docetaxel + Carboplatin + Herceptin + Pertuzumab), 61.6% in (F(5-FU) E(Epirubicin) C(Cyclophosphamide)+H+P)->PTH group and 57.3% in FEC-> TPH group, respectively.

In this study, a 3-year disease free survival (DFS) study was recently published..[4] In the study, patients with pCR showed a higher DFS compared with non-pCR group (HR 0.27, 95% confidence interval (CI): 0.11, 0.64), and additional pertuzumab improved pCR rate and patient's survival.

2) NeoSphere[5]

The NeoSphere study was a global, multicenter randomized Phase II trial of 417 HER2-positive locally advanced breast cancer patients. The aim of this trial was the efficacy of pertuzumab as preoperative chemotherapy ((pathologic complete remission (pCR)). In results, pathological complete response (pCR) rate of PTH therapy (Pertuzumab + docetaxel + Herceptin) was 49%, 31% in the TH group, in 18% in PH group. Therefore, patients who had received PTH therapy highly achieved pathologic complete remission (pCR) compared with other treatment groups.

Recently published article of 5 year progression free survival(PFS), disease free survival, and safety showed that pathologic complete response is the precise surrogate marker of PFS. This study demonstrated that neoadjuvant chemotherapy containing pertuzumab increased pathologic CR rate and decreased 46% of the risk of BC related death. [6]

3) BERENICE[7]

BERENICE is a nonrandomized, phase II, open-label, multicenter, multinational study in patients with normal cardiac function. In the neoadjuvant period, cohort A patients received four cycles of dose-dense doxorubicin and cyclophosphamide, then 12 doses of standard paclitaxel plus four standard trastuzumab and pertuzumab cycles. Cohort B patients received four standard fluorouracil/epirubicin/cyclophosphamide cycles, then four docetaxel cycles with four standard trastuzumab and pertuzumab cycles. The primary end point was cardiac safety during neoadjuvant Treatment. The main efficacy end point was pathologic complete response (pCR, ypT0/is ypN0).

Study populations were 199 and 198 patients in cohorts A and B, respectively. Pathologic CR rates were 61.8% and 60.7% in cohorts A and B, respectively. The highest pCR rates were in the HER2-enriched PAM50 subtype (75.0% and 73.7%, respectively). Hormone receptor (HR) negative BC had higher pCR rate compared with HR positive BC.

4) Adverse events of neoadjuvant TCHP regimen

Neoadjuvant TCHP chemotherapy in HER2-positive early breast cancer is one of the recommended therapies in the current NCCN Clinical Practice Guideline because of high complete remission rate and survival rate. However, this combination chemotherapy frequently caused severe adverse events.

In the TRYPHAENA study, 72.4% of the patients experienced diarrhea, about 40% of the patients complained of nausea and/or vomiting, 46.1% of grade 3 or higher neutropenia, and neutropenic fever occurred in 17.1% of the patients. [3]

According to NeoSphere and TRYPHAENA trials, TCHP regimen was more effective compared with docetaxel, trastuzumab plus pertuzumab combination chemotherapy that achieved 49% complete remission rate, but also had more severe side effects..[5]

2.4 Efficacy and safety data of atezolizumab in HER2 positive breast cancer patients

Atezolizumab, an engineered anti-programmed death-ligand 1 (PD-L1) antibody, is a humanized immunoglobulin G1 monoclonal antibody consisting of two heavy chains (448 amino acids) and two light chains (214 amino acids) and is produced in Chinese hamster ovary cells. PD-L1 is an extracellular protein, which down regulates immune responses primarily in peripheral tissues through binding to its two receptors: programmed death1 (PD-1) and B7.1. Many human tumors overexpress PD-L1, which acts to suppress anti-tumor immunity. PD-1 is an inhibitory receptor expressed on T cells following T-cell activation, which is sustained in states of chronic stimulation such as in chronic infection or cancer. Ligation of PD-L1 with PD-1 inhibits T-cell proliferation, cytokine production, and cytolytic activity, leading to the functional inactivation or exhaustion of T cells. B7.1 is a molecule expressed on antigen-presenting cells and activated T cells. PD-L1 binding to B7.1 on T cells and antigen-presenting cells can mediate down regulation of immune responses, including inhibition of T-cell activation and cytokine production. Overexpression of PD-L1 on TCs has been reported to impede anti-tumor immunity, resulting in immune evasion. Therefore, interruption of the PD-L1/PD-1 and the PD-L1/B7.1 pathways represents an attractive strategy to reinvigorate tumor-specific T-cell immunity.

Atezolizumab targets human PD-L1 and inhibits its interaction with its receptors, PD-1, and B7.1 (CD80, B7-1). Both of these interactions are reported to provide inhibitory signals to T cells. Atezolizumab is being investigated as a potential therapy against solid tumors and hematologic malignancies in humans.

Targeting the PD-L1 pathway with atezolizumab has demonstrated activity in patients with advanced malignancies who have failed SOC therapies. Objective responses have been observed across a broad range of malignancies, including NSCLC, urothelial carcinoma, renal cell carcinoma, melanoma, colorectal cancer, head and neck cancer, gastric cancer, breast cancer, and sarcoma. In particular, available data from the TNBC cohorts in Studies PCD4989g and GP28328 have demonstrated activity and durable responses in patients receiving atezolizumab as monotherapy or in combination with nab-paclitaxel (see the Atezolizumab Investigator's Brochure for detailed efficacy results).

While cancer immunotherapy has demonstrated impressive results in patients with advanced malignancies, emerging data suggest that responses to cancer immunotherapy may be better

when agents are administered in early-stage cancers, where higher levels of tumor-infiltrating lymphocytes (TILs) may be present (Lee et al. 2017; Loi et al. 2017). In particular, this may be more relevant in tumors where TILs are a prognostic factor, such as in early HER2-positive breast cancer.

Cancer immunotherapy is a relatively new focus for breast cancer studies. It is hypothesized that an important mechanism of action of therapeutic antibodies such as trastuzumab and pertuzumab (both humanized monoclonal IgG antibodies) is to induce cellular immunity via interactions with the Fc fragment of the molecule and/or destroy malignant cells, resulting in release of tumor antigens for uptake by antigen-presenting cells, which in turn upregulate immune effector cells. With these mechanisms of action, the combination of trastuzumab and pertuzumab with an anti–PD-L1 inhibitor such as atezolizumab might further enhance anti-tumor immune responses, thereby improving clinical outcomes for patients with minimal increases in clinically significant toxicities.

Preclinical models provide support for this hypothesis. It has been demonstrated that the addition of PD-1:PD-L1 blockade improves the therapeutic activity of a HER2-positive monoclonal antibody in murine experimental model of HER2-positive breast cancer (Stagg et al. 2011; data on file). These findings suggest that anti–PD-L1 immunostimulatory approaches may further capitalize on the immune-mediated effects of therapeutic antibodies. With the potential for therapeutic synergy, combining atezolizumab (anti–PD-L1 antibody) with trastuzumab and pertuzumab may further enhance the anti-tumor efficacy of these established anti-HER2 treatments.

The subtype of breast cancers over-expressing HER2 is the most aggressive. While current therapies have significantly improved patient outcomes, up to 1 in 4 women with HER2-positive EBC will experience recurrence or death within 10–11 years of diagnosis, despite treatment with adjuvant trastuzumab plus standard chemotherapy (Perez et al. 2014; Slamon et al. 2016; Cameron et al. 2017). Patients who received neoadjuvant trastuzumab and pertuzumab with standard chemotherapy, a patient group with tumor size > 2 cm and who are considered at higher risk for recurrence, have demonstrated pCR rates ranging from 45%–68% (Gianni et al. 2012; Schneeweiss et al. 2013; Untch et al. 2016; van Ramshorst et al. 2017; Swain et al. 2018). Even with such rates, 5-year DFS in these patients are around 84% (Gianni et al. 2012) and are expected to be lower in those who are node positive. More recently, data from the APHINITY

study (von Minckwitz et al. 2017) have demonstrated high invasive DFS rates at 3 years (94.1%) and 4 years (92.3%) with adjuvant trastuzumab and pertuzumab plus chemotherapy. However, in patients with node-positive disease, these values were numerically lower at both 3 years (92%) and 4 years (89.9%). As such, there is an opportunity to improve pCR rates and long-term outcomes for patients with high-risk, HER2-positive breast cancer.

Atezolizumab in combination with taxanes has been investigated or is currently being investigated as part of several studies including Studies GP28328, GO29436 (IMpower150), GO29537 (IMpower130), GO30140, WO29522 (IMpassion130), WO39392 (IMpassion031) and WO39391 (IMpassion030) and BO40747 (Impassion050). Atezolizumab in combination with chemotherapeutic regimens including anthracyclines and cyclophosphamide is currently being investigated as part of Studies BO29563, GO29831 and WO39392 (IMpassion031), and WO39391 (IMpassion030). Thus far, reported adverse events observed were similar to those experienced with the individual components of the study treatment and have generally been manageable.

2.5 Atezolizumab combination with HER2 targeted therapy

Ongoing studies exploring atezolizumab combinations in HER2-positive patients include the following:

- Study GO29831 is a Phase Ib open-label, multi-cohort study. This study includes cohorts that evaluate the safety and pharmacokinetics of atezolizumab in combination with trastuzumab and pertuzumab or atezolizumab in combination with trastuzumab emtansine in patients with HER2-positive breast cancer. Enrollment in the study has been completed with a total of 73 patients with HER2-positive breast cancer (early or metastatic).

  With a data cut-off of 17 December 2018, there have been no new safety signals *overall* emerging in the HER2-positive cohorts of Study GO29831 and adverse events are in line with the known safety profile of the individual components of the regimens being evaluated.

- Study WO30085 (Kate2) is a Phase II randomized study of trastuzumab emtansine with or without atezolizumab in HER2-positive, locally advanced unresectable or metastatic breast cancer with prior exposure to a taxane and trastuzumab. This study enrolled 202 patients.

On 22 November 2017, the independent Data Monitoring Committee (iDMC) met and conducted a pre-planned benefit–risk analysis. Following this analysis, the iDMC recommended to the Sponsor that the study be unblinded with respect to treatment assignment to allow investigators and patients to have an open discussion of the potential benefits and risks of continuing the experimental therapy. Unblinding was based upon the improbability of meeting the progression-free survival endpoint, numerically higher rates of serious adverse events that included one fatal event (hemophagocytic syndrome), and numerically higher adverse events leading to discontinuation of study drugs in the atezolizumab arm. The Sponsor issued a "Dear Investigator Letter" on 11 December 2017 communicating the unblinding of the study.

The clinical cutoff date for the primary efficacy analysis was 11 December 2017, at which time 107 PFS events had occurred in the ITT population. For the primary endpoint of PFS based on investigator assessment, the study did not demonstrate a meaningful PFS benefit from the addition of atezolizumab to trastuzumab emtansine in the ITT population. The stratified hazard ratio was 0.82 (95% CI: 0.55, 1.23; p-value = 0.3332). The median PFS for trastuzumab emtansine + placebo was 6.8 months (95% CI: 4.0, 11.1) and for trastuzumab emtansine + atezolizumab 8.2 months (95% CI: 5.8, 10.7). The OS data were immature with a low event rate (11.6% in the trastuzumab emtansine + placebo arm vs. 9.8% in the trastuzumab emtansine + atezolizumab arm).

The safety profile of the combination was consistent with the known profiles of each study drug, and adverse events for the combination were manageable. Although the incidence of Grade ≥3 adverse events was similar between arms with 43.9% in the experimental arm and 41.2% in the control arm, thrombocytopenia, AST elevation, anemia, and pyrexia were numerically increased in the combination arm. A higher incidence of serious adverse events, of adverse events leading to atezolizumab/placebo discontinuation, and of adverse events leading to dose reduction of trastuzumab emtansine was reported in the combination arm. A total of 13 patients (19.1%) in the trastuzumab emtansine + placebo arm experienced at least one serious adverse event compared with 43 patients (32.6%) in the trastuzumab emtansine + atezolizumab arm. The most common serious adverse event reported in the trastuzumab emtansine + atezolizumab arm was pyrexia (10 patients [7.6%]), which was mostly driven by hospitalization for Grade 1 or 2 pyrexia (5.3%), 2 patients (1.5%) experienced Grade 3 pyrexia and 1 patient (0.8%) experienced Grade 4 pyrexia; all 10 patients were reported to have recovered. The most common serious adverse event in the trastuzumab emtansine + placebo arm was seizure (2 patients

[2.9%]), and abdominal pain (2 patients [2.9%]). One patient in the experimental arm experienced a fatal hemophagocytic syndrome. For the trastuzumab emtansine selected adverse events, the incidence of thrombocytopenia, hepatotoxicity, peripheral neuropathy, and infusion-related reaction/hypersensitivity Type 1 were numerically increased in the trastuzumab emtansine＋atezolizumab arm. For the atezolizumab adverse events of special interest, the incidence of immune-related hypothyroidism, immune-related colitis, immune-related rash, and immune-related ocular inflammatory toxicity were numerically increased in the trastuzumab emtansine＋atezolizumab arm compared with the trastuzumab emtansine＋placebo arm.

2.6 Study rationale

Immunogenic potential related with HER2-directed therapy has been reported and it would be one of candidate for combination partner with immune-check point therapeutics (John et al. 2011) Maybe, overcoming resistance to HER2-directed therapy could be an option for other immune-oncologic agents. Hence, characterization of tumor-associated and systemic immune responses to immunotherapy with anti HER2+ and chemotherapies may help us evaluate the applicability of immune therapies and provide rational designs of combination therapies with chemotherapies in these HER2+ BC patients in need of effective treatment, especially for patients with EBC with neoadjuvant aim.

Given the data with our experience and the other reports (NEOSPHERE: Gianni L, et al. Lancet Oncol 2012; 13:25–32), general treatment outcome from tratuzumab plus pertuzumab is about 50% and we assume this as H0 assumption. The most favorable regimen for this setting is TCHP (docetaxel plus carboplatin plus trastuzumab plus pertuzumab), which showed remarkable pCR rate of approximately 60% [RWD is warranted: Total pCR rate: 65% (pCR rate: ER- , beyond 80%; ER+, about 50%), unpublished local source of data which is shown pCR rate with TCHP treatments below)].

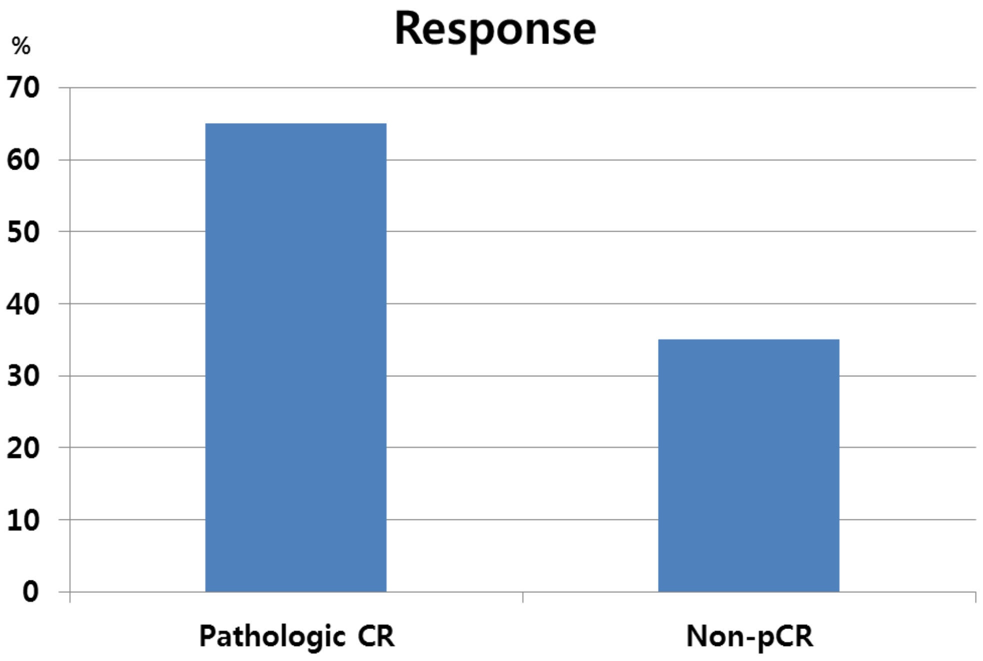

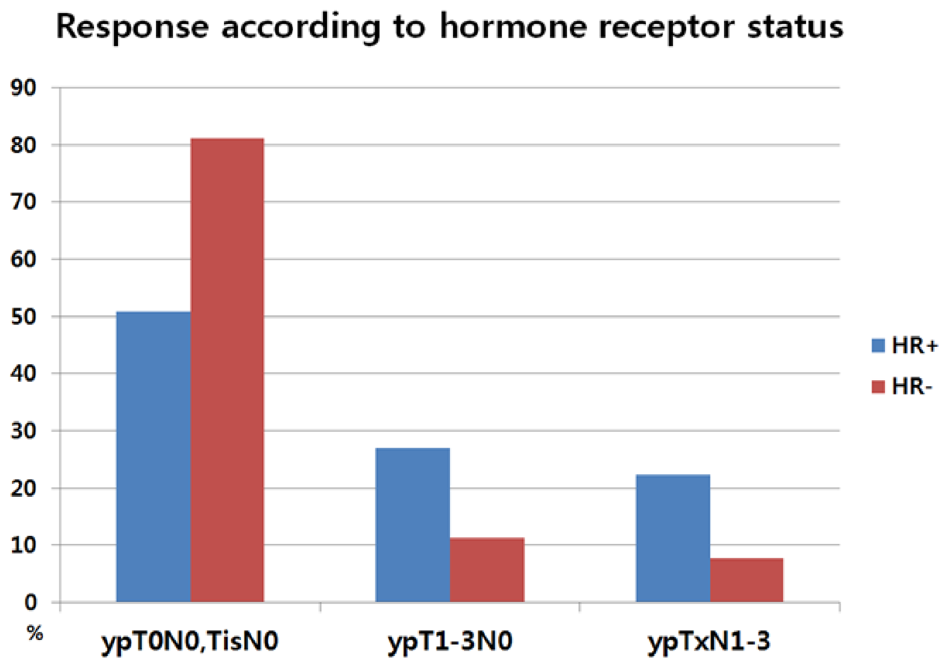

However, toxicities of the regimen are pretty serious; myelosuppression including neutropenic fever (NF), neurotoxicity, nephrotoxicity, emesis and etc. Thus, the patients who have co-morbidities and old ages are difficult to treat TCHP regimen considering comprehensive benefit/risk, and there are still drug-related serious events associated with serious toxicities such as grade 3 or higher grade of diarrhea, hematochezia, and sepsis . If atezolizumab enhances

combination chemotherapy with taxane plus HER2-doublet, this agent could replace carboplatin as well as mitigating serious toxicities of carboplatin (anemia, neutropenic fever, thrombocytopenia, grade 2 or more neurotoxicity, nephrotoxicity, and emesis) as well as improving QoL.

Results from Study BO27938 (Katherine) demonstrated a significant reduction in the risk of recurrence of invasive breast cancer or death in patients with HER2-positive early breast cancer with residual invasive disease after completion of neoadjuvant therapy who were treated with adjuvant trastuzumab emtansine compared with those treated with adjuvant trastuzumab (HR = 0.50; 95% CI, 0.39–0.64; p < 0.001) (von Minckwitz et al. 2019). These data resulted in a recent update of the NCCN guidelines that recommend trastuzumab emtansine for patients with residual disease for 14 cycles. The guidelines further recommend to switch treatment to trastuzumab ± pertuzumab in patients who have to discontinue trastuzumab emtansine for toxicity to complete 1 year of HER2-targeted therapy (NCCN 2019). The adjuvant treatment options offered in the study (ML40985, NeoPATH) for patients not achieving pCR are trastuzumab emtansine in combination with atezolizumab. This study (ML40985, NeoPATH) will explore trastuzumab emtansine in combination with atezolizumab in patients with non-pCR with exposure in a limited number of patients.

This enriched population may have main microenvironmental characteristics to define role of immunogenicity through multi-omic profiling. In addition, relationship between cross-talk pathways will be revealed including PI3K pathway, which is considered as one of main resistance mechanism to HER2-directed therapy.

Biomarker development for adapting immune-oncologic agents is underway including immunohistochemistry for PD1 and PD-L1 expression, mutational loads, and other genes expression profiling. For patients with primary refractory HER2+ EBC, there is un-met medical need to select right patients for right drugs add on atezolizumab for their adjuvant treatments together with trastuzumab and pertuzumab.

Recently, introduction of gene expression profiling (GEP) has brought much potential to expand immune-oncologic agents with or without combination of the other conventional treatments including HER2-directed agents to overcoming resistance to HER2 pathway especially for patients with HER2+ BCs as well as TNBCs.

We've already established genomic platform for BC patients. Using this genomic platform and experience, I'd like to propose IO and mutational profiling for HER2+ EBC patients. We are to better define those HER2-positive BCs who may benefit from add on atezolizumab to trastuzumab

through multi-omic profiling using F1CDx.

3. Study Objectives and Endpoints

3.1 Primary objective:

To evaluate the pCR (pathologic complete response) rate of TAHP neoadjuvant chemotherapy with the patients with HER2+ EBC

3.2 Secondary objectives:

To evaluate the EFS of atezolizumab add-on adjuvant treatments with Herceptin SC plus pertuzumab in patients with Breast Cancer of patients with pCR versus non-pCR

To assess the efficacy of atezolizumab add-on adjuvant treatments according to PD-L1 status by IHC (IC0 and IC1/2/3 subgroup analysis)

**Objectives**	**Corresponding Endpoints**
Primary Objective:	
To evaluate the pCR rate of TAHP neoadjuvant chemotherapy with the patients with HER2+ EBC	Pathologic complete response rate (pCR)
Secondary Objective:	
➢ To evaluate the EFS of atezolizumab add-on adjuvant treatments with herceptin SC plus pertuzumab in patients with Breast Cancer of patients with pCR versus non-pCR<br>➢ To assess the efficacy of atezolizumab add-on adjuvant treatments according to PD-L1 status by IHC(IC0 and IC1/2/3 subgroup analysis)	➢ 3-year Event free survival (EFS) of the patients with pCR vs. non-pCR to TAHP<br>➢ Overall survival (OS)<br>➢ Disease Free Survival (DFS)<br>➢ pCR and EFS based on PD-L1 status Quality of Life evaluated by FACT-T questionnaire
Safety Objectives	
To determine the safety and tolerability of atezolizumab add-on adjuvant treatments	Incidence and severity of adverse events, AESIs and SAEs with severity determined according to

with Herceptin SC plus pertuzumab for remaining 1 year in patients with Breast Cancer	NCI CTCAE v5
Exploratory Biomarker Objectives using F1CDx	
1. To evaluate efficacy of Neo-adjuvant TAHP chemotherapy based on HER2 amplification (level: high-low)<br>2. To assess the role of PI3K on resistance of HER2 positive breast cancer patients<br>3. To evaluate correlation between TMB and clinical outcome or any genetic environmental change to response to therapy.	➢ Multi-omic profiling using F1CDx and WTS platform in house<br>➢ Immune signatures such as PD-L1 expression, tumoral mutation burden (TMB), Intrinsic subtyping, mutation signature, pathway analysis including PIK3CA and HER<br>➢ Single cell sequencing of residual cancer after neoadjuvant PATH<br>➢ Cf DNA profiling

4. Study treatment

4.1 Investigational medicinal products

4.1.1 Docetaxel

Brand name	Taxotere1-Vial Inj
Formulation	vial
Dosage	75 mg/m^2
Administration	intravenous injection
Cycle	Every 3 weeks (21days)
Storage	Sealed container, 2-25℃, Shade storage
Supportor	Sanofi-Genzyme

4.1.2 Atezolizumab

Brand name	Tecentriq Inj
Formulation	vial
Dose	1200mg

Administration	intravenous injection
Cycle	Every 3 weeks (21days)
Storage	Sealed container, 2-8℃ storage
Supportor	Roche

4.1.3 Herceptin sc

Brand name	Herceptin subcutaneous Inj
Formulation	vial
Dose	600mg
Administration	subcutaneous injection in thigh for 2-5minutes
Cycle	Every 3 weeks (21days)
Storage	Sealed container, 2-8℃ storage
Supportor	Roche

4.1.4 Pertuzumab

Brand name	Perjeta Inj
Formulation	vial
Dose	840mg loading dose followed by 420mg
Administration	intravenous injection
Cycle	Every 3 weeks (21days)
Storage	Sealed container, 2-8℃ storage
Supportor	Roche

4.1.5 Trastuzumab Emtansine

Brand name	Kadcyla Inj
Formulation	vial
Dose	3.6mg/kg
Administration	intravenous injection
Cycle	Every 3 weeks (21days)
Storage	Sealed container, 2-8℃ storage
Supportor	Roche

4.1.6 Tripegfilgrastim

Brand name	Tripegfilgrastim Inj
Formulation	Syringe
Dose	6mg/0.6ml
Administration	subcutaneous injection
Cycle	Every 3 weeks (21days)
Storage	Sealed container, 2-8℃ storage
Supportor	DongA ST

5. Concomitant Therapy

5.1 Prohibited Therapy

The following treatments are prohibited during all phase of treatment

1) Cytotoxic chemotherapy,

2) Radiotherapy (excluding adjuvant radiation therapy for breast cancer after completion of chemotherapy)

3) Chemotherapy other than that administered in this clinical study, including immunotherapy and biological chemotherapy

4) All targeted therapies (Targeted therapy)

5) Live, attenuated vaccines

6) Systemic immunostimulatory agents (including, but not limited to, interferons and IL 2) are prohibited within 4 weeks or 5 half-lives of the drug (whichever is longer) prior to initiation of study treatment and during study treatment because these agents could potentially increase the risk for autoimmune conditions when given in combination with atezolizumab.

7) Systemic immunosuppressive medications (including, but not limited to, cyclophosphamide, azathioprine, methotrexate, and thalidomide) are prohibited during study treatment because these agents could potentially alter the efficacy and safety of atezolizumab.

5.2 Permitted Therapy

In general, all medications taken by the patient for concomitant diseases should continue during the study treatment period and EOT visit and should be recorded on the eCRF. The following list

of allowed medications is provided as a guidance, treatment prescribed to the patients should be adapted according to local standard practice.

1) H1 and H2 antagonist (e.g. diphenhydramine, cimetidine)
2) Analgesics (e.g. paracetamol, meperidine, acetaminophen, opioids)
3) Corticosteriods to treat or prevent allergic or infusion reactions
4) Antiemetics (approved prophylactic serotonin-antagonists, benzodiazepines, ondansetron etc)
5) Medication to treat diarrhea (e.g. loperamide)
6) Colony stimulating factors (e.g. G-CSF)
7) Estrogen receptor antagonist (e.g. tamoxifen) or aromatase inhibitors (e.g. anastrazole, exemestane) after completion of post-operative chemotherapy as per local practice as indicated
8) Acceptable methods of contraception must be used when the female patient or male partner is not surgically sterilized or does not meet the study definition of post-menopausal (≥ 12 months of amenorrhea).
9) Postoperative patient management may include radiotherapy as clinically indicated, and management of patients who do not achieve a pCR should follow current SOC guidelines. Radiotherapy may be given concurrent with HER2 therapy and atezolizumab.

6. Study population

HER2 positive early breast cancer diagnosed as stage IIA-IIIC according to AJCC 7th staging system

7. Patients

7.1 Inclusion Criteria

1) Patients with locally advanced, inflammatory or early stage of histologically confirmed diagnosis of breast cancer
2) Primary breast tumor site of >2 or nodal involvement was confirmed at the same side of breast by fine needle aspiration (determined by AJCC 7th edition, Clinical stage IIA-IIIC)
3) Eastern Cooperative Oncology Group Performance of 0-1

4) Patient has HER2-positive breast cancer as 3+ by IHC or gene amplified by in situ hybridization (ISH) as defined by a ratio ≥ 2.0
5) Available to provide tumor sample
6) LVEF ≥55% at baseline by ECHO or MUGA
7) Patient is ≥ 19 years old
8) Patient has no possibility of pregnancy or Urine or blood b-HCG result is negative.
9) Patient has adequate bone marrow function (ANC ≥ 1,500/ul, Platelet ≥100,000/ul, Hemoglobin ≥ 9.0 g/dl)
10) Patient who are not receiving therapeutic anticoagulant: INR or a PTT≤ 1.5* ULN. Patient who are receiving therapeutic anticoagulant need to on a stable anticoagulant regimen.
11) Patient has adequate liver function (Serum bilirubin≤ 1.5* upper limit of normal and AST/ALT 1.5*upper limit of normal)
12) Patient agrees to use effective contraception or patient has no possibility of pregnancy
13) To obtain written informed consent
14) To agree tissue and blood collection for biomarker evaluation

7.2 Exclusion Criteria

1) Patients who have metastatic disease (M1)
2) Tumor size less than 2cm and N0
3) History of secondary malignancy (except in situ carcinoma of the cervix, adequately treated non-melanoma skin cancer, thyroid papillary carcinoma or malignancy history which is over 5 years after treatment completion without relapse)
4) Any previous treatment against including chemo, hormonal therapy
5) Administration of a live, attenuated vaccine within 4 weeks before Day 1 or anticipation that such a live attenuated vaccine will be required during the study and 5 months after last dose of atezolizumab.
6) Severe infections within 4 weeks prior to Day 1, including, but not limited to, hospitalization for complications of infection, bacteremia, or severe pneumonia. Signs or symptoms of significant infection within 2 weeks prior to Day 1
7) Received oral or IV antibiotics within 2 weeks prior to Cycle 1 Day 1
8) Active tuberculosis or pneumonia
9) History of idiopathic pulmonary fibrosis

10) Known clinically significant liver disease, including alcoholic, hepatitis B or C infection

A. Patients with past or resolved hepatitis B infection (defined as having a negative HBsAg test or a positive hepatitis B core antigen [anti-HBc] test with confirming HBV DNA negative results) are eligible.

B. Patients positive for hepatitis C virus (HCV) antibody are eligible only if polymerase chain reaction assay (PCR) is negative for HCV RNA

C. Known clinically treatment required liver disease, including cirrhosis, inherited liver disease and idiopathic hepatitis

11) Inadequate kindny function such as serum creatinine ≥ 1.5 * Upper Limit of Normal or Serum Creatinine is within 1.0-1.5 * Upper Limit of Normal and eGFR < 60 mL/min/1.73m^2

12) Uncontrolled hypertension (systolic blood pressure >180mmHg and/or diastolic blood pressure >100mmHg), Unstable Angina Pectoris, Congestive Heart Failure, significant arrhythmias (i.e atrial fibrillation, paroxysmal supraventricular tachycardia), Myocardial infarction or LVEF < 55% within 6 months prior to randomization

13) Total bili >1.5 ULN (except for Gilbert's syndrome), AST/ALT > 1.5 ULN, ALP > 2.5 ULN

14) Immunosuppression or known HIV infection

15) Dyspnea at rest which is required for continuous oxygen therapy or other lung disease

16) Uncontrolled serious infectious disease

17) Active heart disease such as myocardial infarction, angina pectoris, uncontrolled arrhythmias within 6 months and uncontrolled severe medical, psychiatric disease

18) Patients with prior allogeneic stem cell or solid organ transplantation

19) Pregnant or lactating or intending to become pregnant during or within 7 months after the last dose of study treatment

20) History of hypersensitivity for IMP or excipient

21) History of autoimmune disease including, but not limited to, systemic lupus erythematosus, rheumatoid arthritis, inflammatory bowel disease, vascular thrombosis associated with antiphospholipid syndrome, Wegener's granulomatosis, Sjögren's syndrome, Bell's palsy, Guillain-Barré syndrome, multiple sclerosis, vasculitis, or glomerulonephritis

22) Patients who are not available to provide tumor tissue

23) Patient who can't comply with the protocol and who is not willing to comply with the protocol

7.2.1 Exclusions Related to Trastuzumab Emtansine in the Adjuvant Setting:

- Patients who achieved pCR defined as the absence of residual invasive cancer on hematoxylin and eosin evaluation of the complete resected breast specimen and all sampled regional lymph nodes following completion of neoadjuvant systemic therapy (i.e., ypT0/is ypN0 in the current AJCC staging system, 8th edition)
- Evidence of clinically evident gross residual or recurrent disease following neoadjuvant therapy and surgery
- Unable to complete surgery with curative intent after conclusion of neoadjuvant systemic therapy
- Patient discontinued treatment with trastuzumab because of toxicity during the neoadjuvant phase of the study
- Clinically significant history of liver disease, including cirrhosis, current alcohol abuse, autoimmune hepatic disorders, or sclerosis cholangitis
- Patients with Grade $\geq$ 2 peripheral neuropathy
- Prior treatment with trastuzumab emtansine
- Serum AST, ALT, and alkaline phosphatase not within $\leq 1.5 \times$ ULN
- Serum total bilirubin not within normal range ($\leq 1.0 \times$ ULN)
  Except for patients with Gilbert's syndrome, for whom direct bilirubin should be within the normal range
- Serum creatinine not within <1.5 x ULN

7.3 Determination of sample size

7.3.1 Sample size

67 patients

7.3.2 Determine of sample size

Primary endpoint of this study is to investigate the pathologic complete response rate (pCR rate) of docetaxel + trastuzumab + pertuzumab in combination with atezolizumab in HER2-positive breast cancer patients as neoadjuvant chemotherapy compared with docetaxel plus carboplatin plus trastuzumab plus pertuzumab (65% complete remission).

In this case, when we try to test the hypothesis at the significance value (P-value) of 10% and the power of 80% by applying Simon's two stage optimal design with the null hypothesis (H0) pCR rate = <50% and the alternative hypothesis (h1) pCR rate> = 65%

Step 1: If 23 patients were recruited and fewer than 12 patients had pathologic complete remission among the 23 patients, the study drug was rejected and the study was terminated.

Step 2: If more than 13 patients were diagnosed with pathologic complete remission, 37 patients were recruited (final 60 patients). If the number of patients who achieved pathologic complete remission was less than 34, the study drug was rejected. If more patients (more

than 60) are recruited in Step 2 in consideration of the 10% dropout rate, the criteria for reject test drugs according to the final number of people eligible under the 10% significant level are as follows. (ref: “Proper inference from Simon’s two-stage designs” Tatsuki Koyama and Heidi Chen, Statistics in Medicine 2008; 27(16): 3145-3154)

Total enrolled patients	Non-PCR patients
61	36
62	36
63	37
64	38
65	38
66	39
67	39

The total study period including the patient recruitment period of 12 months and the follow - up period of 36 months will be 48 months.

7.4 Stratification factors

(1) Hormone receptor (Estrogen receptor and/or progesterone receptor) status

(2) PD-L1 status

8. Duration of study

Duration of study is expected to be 48 months (patient enrollment: 12 months and follow up period : 36 months). Duration may be extended depending on the recruitment status. [2019.05 - 2023.4 (48 months)]

9. Study design

This study was phase IB-II clinical trial that designed to evaluate the efficacy and safety of docetaxel + atezolizumab + Herceptin sc plus pertuzumab plus adjuvant therapy of atezolizumab + trastuzumab + pertuzumab after surgery in female patients with HER2-positive early breast cancer. The scheme is present as below.

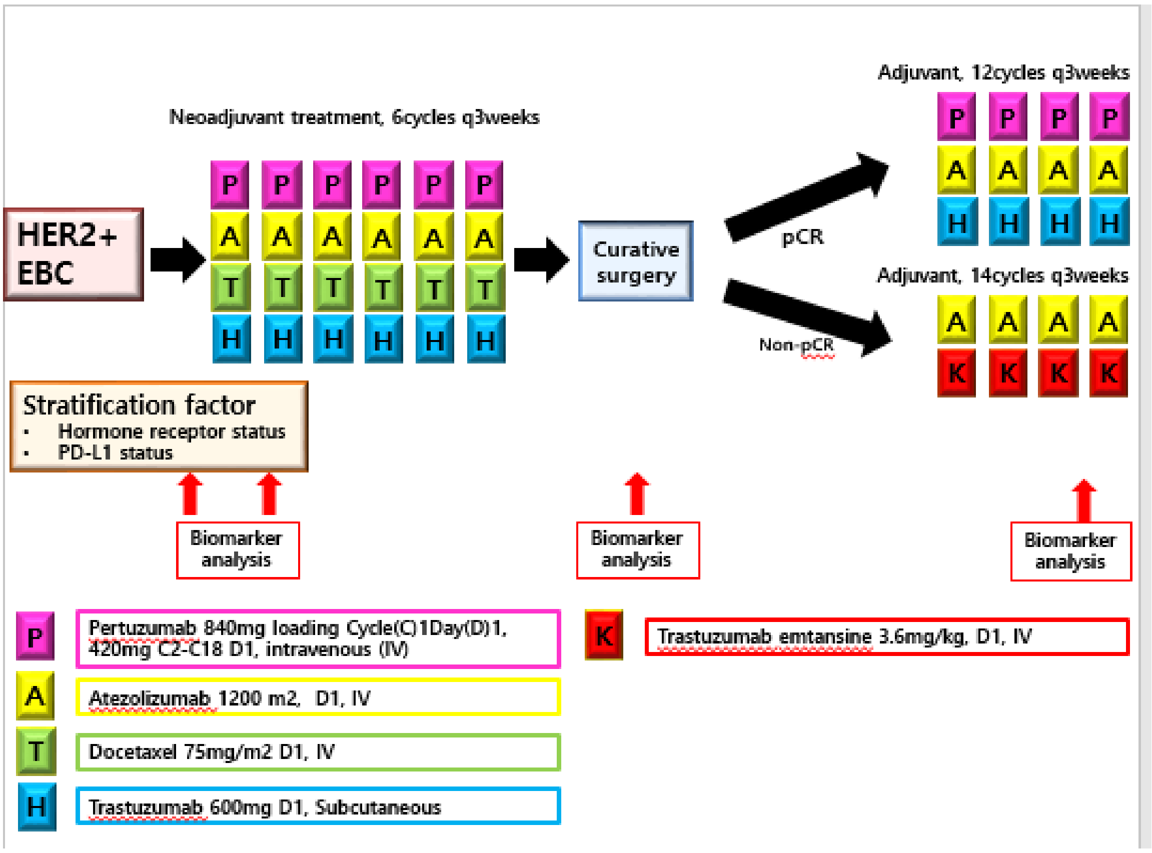

Drug administration

(A, Neoadjuvant setting): Docetaxel (75mg/m2, intravenous(IV)) Day(D)1, Atezolizumab (1200mg, IV) D1, Herceptin sc (600mg subcutaneous(SC))D1 and Pertuzumab (840mg loading dose at Cycle 1 followed by 420mg(IV))D1 X 6 cycles q3weeks, intravenous(IV) administration

(B, Adjuvant setting) :

patients with pCR:

Atezolizumab (1200mg, IV), Trastuzumab (600mg, SC) and Pertuzumab (840mg loading dose at Cycle 1 followed by 420mg, IV- reloading is required in case the infusion interval 6 weeks or more) D1 X 12 cycles q3weeks

patients with non-pCR:

Atezolizumab (1200mg, IV) and Trastuzumab Emtansine(3.6mg/kg, IV) D1 X 14 cycles q3weeks

* As per investigator’s discretion, Tripegfilgrastim 6mg/EA can be administered 24 hours after cytotoxic chemotherapy in order to reduce the period for moderate neutropenia at Neoadjuvant setting.

Investigational product would be administrated to patients till disease progression, uncontrolled toxicities, drop out and/or other unacceptable reasons to discontinuation of drug administration.

10. Schedules of assessments and procedures

10.1 Experimental group

Visit	Screening[1)]		Neoadjuvant chemotherapy (TAHP)		OP	Adjuvant treatment	End of treatment [12)]	Follow up	
			1,2,3 cycle	4,5,6 cycle		AHP (pCR), AK (non-pCR) 1-12 cycle / 1-14 cycle			
Visiting date			Day1	Day1		Day1	28 days after last treatment	3 months after last treatment [6)]	every 6month [11)]
Visit window	-28 days	-7 days	±5 days	±5 days		±5 days	+7 days	+ 14 days	±30 days
Informed consent	●								
Inclusion/exclusion criteria	●								
Baseline characteristics	●								
Height	●								
Weight	●		●	●		●	●		
Previous and current medical history (history of breast cancer treatment)	●								
Physical examination	●		●	●		●	●		

Visit	Screening[1]		Neoadjuvant chemotherapy (TAHP)		OP	Adjuvant treatment	End of treatment [12]	Follow up	
			1,2,3 cycle	4,5,6 cycle		AHP (pCR), AK (non-pCR) 1-12 cycle / 1-14 cycle			
Visiting date			Day1	Day1		Day1	28 days after last treatment	3 months after last treatment [6]	every 6month [11]
Visit window	-28 days	-7 days	±5 days	±5 days		±5 days	+7 days	+ 14 days	±30 days
Concomitant medication	●		●	●		●	●		
Vital sign[2]/ ECOG	●		●	●		●	●		
EKG, ECHO or MUGA scan	●			● (after 6th cycle)		● (q 6 cycles)	●		
CBC		●	●	●		●	●		
Chemistry profile including [3]		●	●	●		●	●	●	
thyroid function test (TFT) [4]		●	●	●		●	●		

Visit	Screening[1]		Neoadjuvant chemotherapy (TAHP)		OP	Adjuvant treatment	End of treatment[12]	Follow up	
			1,2,3 cycle	4,5,6 cycle		AHP (pCR),<br>AK (non-pCR)<br>1-12 cycle / 1-14 cycle			
Visiting date			Day1	Day1		Day1	28 days after last treatment	3 months after last treatment[6]	every 6month[11]
Visit window	-28 days	-7 days	±5 days	±5 days		±5 days	+7 days	+ 14 days	±30 days
Coagulation panel		●							
Blood Endocrine Test[5]		●		●<br>(Every 12 weeks based on C1D1)				●	
Urine test		●	●	●		●	●	●	
Pregnancy test[7]		●		●<br>(after 6th cycle)		●<br>(q 6 cycles)	●		
IP administration			●	●		●			

Visit	Screening[1]		Neoadjuvant chemotherapy (TAHP)		OP	Adjuvant treatment	End of treatment[12]	Follow up	
			1,2,3 cycle	4,5,6 cycle		AHP (pCR), AK (non-pCR) 1-12 cycle / 1-14 cycle			
Visiting date			Day1	Day1		Day1	28 days after last treatment	3 months after last treatment[6]	every 6month[11]
Visit window	-28 days	-7 days	±5 days	±5 days		±5 days	+7 days	+ 14 days	±30 days
Tumor measure via physical examination	●		●	●					●
Pathological Tumor stage evaluation					●				
Tumor measurement and response evaluation[8]	●		**Response evaluation and quality of life questionnaires were performed after 3rd cycle, 6th cycle of**			**Response evaluation were performed every 6 months**(±60 days) **after surgery**			
QoL evaluation using FACT-T questionnaire	●					**Quality of life questionnaires were performed**	●		●

**Visit**	**Screening[1)]**		**Neoadjuvant chemotherapy (TAHP)**		**OP**	**Adjuvant treatment**	**End of treatment [12)]**	**Follow up**	
			**1,2,3 cycle**	**4,5,6 cycle**		**AHP (pCR), AK (non-pCR) 1-12 cycle / 1-14 cycle**			
Visiting date			Day1	Day1		Day1	28 days after last treatment	3 months after last treatment [6)]	every 6month [11)]
Visit window	-28 days	-7 days	±5 days	±5 days		±5 days	+7 days	+ 14 days	±30 days
			**neoadjuvant chemotherapy**			**every 6 months after surgery**			
Tissue and blood sampling for biomarker analysis[10)]	●		● (before 2nd cycle)		●	● (after disease progression)			
Investigation of AEs			●	●		●	●	●	
Survival f/u[13)]									●

1) A screening examination should be performed between -7 and -1 days before the first study dose (Day 1), except for those procedures that may be conducted up to four weeks prior to Day 1 as specified in the schedule of assessments. Patients who fulfill all the inclusion and none of the exclusion criteria will be accepted into the study.

2) Vital sign included blood pressure, pulse rate and body temperature.
3) Results of standard-of-care tests or examinations performed prior to obtaining informed consent and within 6 weeks prior to C1D1 (except where the subject condition is worsening) may be used for baseline assessment rather than repeating such tests.
4) Chemistry profile included as follow: albumin, bilirubin, and AST/ALT/ALP, BUN/Cr, electrolyte.
5) Thyroid function test (TSH, T3, Free T4) was performed
6) Blood endocrine test will be checked at screening, every 12 weeks based on Cycle 1 Day 1 and 3 months after last treatment.
7) Blood endocrine test, Urine Analysis, Blood chemistry, Thyroid function test(TSH) will be followed up 3 month after treatment completion.
8) Serum pregnancy test for women of childbearing potential at screening/baseline (within 7 days before Cycle 1, Day 1); urine pregnancy tests will be performed at every 6 cycle and at treatment discontinuation. If a urine pregnancy test is positive, it must be confirmed by a serum pregnancy test. A woman is of childbearing potential if she is postmenarcheal, has not reached a postmenopausal state (≥ 12 continuous months of amenorrhea with no identified cause other than menopause), and has not undergone surgical /chemical sterilization (removal of ovaries and/or uterus or received monthly GnRH agonist).
9) Baseline breast ultrasound, breast MRI, Chest and abdomen- pelvic CT, bone scan, check X-ray(if required) should be performed within 6 weeks before Cycle 1 day 1. Imagining results performed prior to obtaining informed consent and within 6 weeks prior to C1D1 (except where the subject condition is worsening) may be used for baseline assessment rather than repeating such tests. PET/CT may replace to perform chest, abdomen-pelvic CT and bone scan.
10) Tumor evaluation is performed after the third cycle and the sixth cycle (before surgery) according to the delayed cycle or discontinuation of the therapy. After surgery, tumor evaluation is performed every 6 months.
11) All subjects should agree to provide a tissue biopsy sample at the time of diagnosis and at the time of surgery in patients whose complete remission has not occurred. When disease recurrence occurred, tissue biopsy if possible.

(Mandatory: at screening and at surgery(non-pCR) / Optional: before 2nd cycle and after disease progression)

In addition, a blood sample will be obtained for biomarker analysis. (Mandatory: at screening and at surgery and before 2nd cycle and after disease progression)

12) Patients will be followed for disease status and progression (if progression has not yet occurred), anti-cancer treatments and survival status every 6 months after 3 months after their last treatment completion. Follow up will be maintained 36 months from the last patients enrollment date.

13) After study treatment discontinuation, subject should have the end of treatment follow up visit after 28 days from the last study treatment. (Safety follow up visit)

14) In case of study treatment completion due to disease progression or initiating new anti-cancer treatment, End of treatment visit will be performed either 28 days(+ 7 days) from the last study treatment completion or new anti-cancer treatment imitation which is occurred first. Survival follow up will be conducted to evaluate the status every 12 weeks via chart review or phone contact until subject death, withdrawal consent or study completion which is occurred first.

10.2 List of evaluation

10.2.1 Screening

All subjects must provide written informed consent before any study specific assessments or procedures are performed. A screening examination should be performed between -7 and -1 days before the first study dose (Day 1), except for those procedures that may be conducted up to four weeks prior to Day 1 as specified in the schedule of assessments. Patients who fulfill all the inclusion and none of the exclusion criteria will be accepted into the study.

(1) Demographics and height/weight

Height will be measured at screening and weight will be measured at each cycle to allow calculation of body surface area (BSA) and treatment dose. Weight is measured on Day 1 of each cycle and compared to baseline. If ± 10% variation occurs the chemotherapeutic doses will be recalculated.

(2) Medical history and/or concomitant medication

A complete medical history (including demographics) will be performed at screening.

(3) Physical Examination

(4) Vital sign / ECOG performance status

Vital sign included blood pressure, pulse rate and body temperature. Performance Status (PS) will be measured using the ECOG Performance Status Scale [Appendix 1]

(5) EKG, Echocardiogram and MUGA scan

A baseline ECG is performed for all patients. ECGs are required to allow assessment prior to and after the completion of neoadjuvant chemotherapy. For all patients, ECG to be performed within ≤ 7 days prior cycle1 day1. For patients whose LVEF cannot be assessed by ECHO, LVEF can be assessed by MUGA. The same method should be used throughout the study for each patient and preferably performed and assessed by the same assessor.

(6) Laboratory tests

Hematology and biochemistry will be done as part of regular safety assessments. During the study, a complete blood count and biochemistry analyses will be performed prior to administration of study medication (Day 1 of each cycle).

CBC	WBC, RBC, hemoglobin, hematocrit, platelet, neutrophil, eosinophil, basophil, lymphocyte, monocyte, ANC
Coagulation panel	PT, a PTT, INR
Chemistry profile	albumin, total bilirubin, AST, ALT, ALP, BUN, Creatinine
Electrolyte	Na, K, Cl
Blood endocrine test	HbA1c, Glycoalbumin, Lipid profile(cholesterol, triglyceride, HDL cholesterol, LDL cholesterol), C-Peptide, Insulin
Pregnancy test	Urine or serum or both
Thyroid function test	TSH, T3, Free T4
Urine test	RBC, protein

(7) Tumor measurement and Response evaluation

Baseline total tumor burden must be assessed within a maximum of 4 weeks before first dose of study drug treatment. Post-baseline tumor assessments will be done up to 7 days prior to dosing (Cycle 4) and surgery. If there is suspicion of disease progression based on clinical or laboratory findings before the next scheduled assessment, an unscheduled assessment should be performed.

If a patient inadvertently misses a prescribed tumor assessment or a technical error prevents the evaluation, the patient may continue treatment until the next scheduled assessment, unless signs of clinical progression are present.

(8) Patient-Reported Outcomes (FACT-T)

Patient-reported outcomes are performed at baseline, after 3 and 6 cycles of chemotherapy using FACT-T questionnaire. After curative surgery, Patient-reported outcomes are assessed every 6months.

(9) Biomarker analysis

All subjects should agree to provide a tissue biopsy sample at the time of diagnosis and at the time of surgery in patients whose complete remission has not occurred. In addition, a blood

sample will be obtained for biomarker analysis. When disease recurrence occurred, tissue biopsy can be performed if possible.

There will be a separate manual protocol for sample collection and NGS experiments.

10.2.2 Drug administration (Visit window ±5 days) and End of Treatment visit

Weight is measured on Day 1 of each cycle and compared to baseline. If ± 10% variation occurs the chemotherapeutic doses will be recalculated.

(1) Body weight

The amount to be administered must be recalculated if the patient's body weight has changed by > 10% (increased or decreased) from baseline.

(2) Physical examination

Any clinically significant findings that newly occurred after the IP administration would be recorded as an adverse reaction.

(3) Concomitant drug

(4) Vital sign / ECOG performance status

(5) ECG, ECHO and MUGA scan

The same method should be used throughout the study for each patient. After neo-adjuvant 6th cycle, during adjuvant 6 cycles and end of treatment. At the end of treatment visit, MUGA scan can be replaced by echocardiogram in case considered any safety concerns. If alternative echocardiogram is not available to check, test result in 12 cycles of adjuvant therapy can be used.

(6) Laboratory tests : CBC, Chemistry profile, Thyroid function test, Urine test, Pregnancy test (after neo-adjuvant 6th cycle, every adjuvant 6 cycles.) After immune check point inhibitor treatment, blood endocrine test (HbA1c, Glycoalbumin, Lipid profile (cholesterol, triglyceride, HDL cholesterol, LDL cholesterol) will be monitored every 12 week based on TAHP cycle 1 Day 1.

(7) Investigational Medicinal Product Administration

IMP administration is described at appendix 8 IMP administration guideline. Adjuvant HER2 targeted therapy will maintain as planned in protocol even HER2 result from IHC, ISH is negative after surgery. First adjuvant treatment shall not begin up to 2 weeks after surgery and shall be administered within 45 days after surgery.

(8) Tumor measurement and Response evaluation

Tumor assessment is conducting after the 3 and 6 cycles of neo-adjuvant therapy that is adjusted according to the delayed the cycle or treatment discontinuation. During adjuvant therapy, tumor assessment is conducting every 6 months after the surgery. . Tumor assessment during neo-adjuvant therapy is evaluated by breast MRI (mandate), the other images are also considered to follow up based on investigator's discretion. Tumor assessment during adjuvant therapy is evaluated for disease progression/recurrence by the images (i.e breast MRI, breast ultrasounds, mammography, chest or abdomen/pelvic CT, bone scan, PET/CT etc) based on investigational sites' SOC and investigator's discretion. Evaluation of tumor response will be evaluated for target and non-target lesion per RECIST v1.1. If the subject shows a disease progression but the investigator decides to apply iRECIST, the subject who is clinically stable condition can continue the treatment. In that case, investigator evaluates to confirm disease progression through iRECIST. To allow study treatment despite the initial radiologic progression takes into account the observation that subjects may experience a rapid deterioration of tumor and then show a response of the immunotherapy.

Clinically stable condition is defined as below:

-No signs or symptoms indicating clinically significant progression of the disease

-No deteriorate of ECOG

-No intensive care is required including increased pain control, radiotherapy or other palliative treatment.

(9) Patient-Reported Outcomes (FACT-T)

Patient-reported outcomes are performed at baseline, after 3 and 6 cycles of chemotherapy using FACT-T questionnaire. After curative surgery, Patient-reported outcomes are assessed at study treatment completion visit and then followed up every 6months.

(10) Biomarker analysis

(11) Pathological tumor staging

Primary endpoint (pCR) will be evaluated based on providing template by investigational sites examination.

(12) Adverse Events

Adverse event will be collected after initial dose of Neo-adjuvant treatment and will be followed up until 28 days after last treatment. The event is occurred prior to neo-adjuvant therapy will be

recorded as medical history.

10.2.3 Follow up visit

Blood endocrine test (HbA1c, Glycoalbumin, Lipid profile (cholesterol, triglyceride, HDL cholesterol, LDL cholesterol), Urine Analysis, Blood chemistry, Thyroid function test will be followed up 3 month after treatment completion.

After treatment completion visit, patients will be followed for disease status and progression (if progression has not yet occurred), anti-cancer treatments and survival status every 6 months for at least 36 months from randomization.

11. Dose modification

The amount of docetaxel is calculated according to the patient's body surface area (BSA). Weight and height should be recorded at baseline and the BSA calculated, thereafter at every scheduled visit for all patients should be re-weighed. The amount to be administered must be recalculated if the patient's body weight has changed by > 10% (increased or decreased) from baseline. If there is a possibility of duplicate toxicity, the attribution of an adverse event to a possible drug is based on the investigator's discretion.

11.1 Docetaxel

Dose reductions should be managed. A stepwise dose reduction is permitted. Once a dose reduction has occurred the patient should not be re-escalated to higher doses.

Dose delays will be allowed for myelosuppression, hepatic dysfunction, and other dose limiting toxicities. A two week dose delay will be permitted to allow recovery to baseline per cycle. One further week dose delay will be permitted before the patient must be discontinued from the study. The dose modification can be changed according to the policy of institution

11.2 Atezolizumab

Dose reduction of atezolizumab is not permitted in this study. Atezolizumab infusions may be temporarily suspended in case of an adverse event that requires a dose to be held. If atezolizumab is held because of adverse events for >12 weeks beyond the last dose, then the

patient will be discontinued from atezolizumab treatment and will be followed for safety. If, in the judgment of the investigator, the patient is likely to derive clinical benefit from resuming atezolizumab after a hold of > 12 weeks, study drug may be restarted with the approval of the Coordinating investigator.

11.3 Herceptin sc and pertuzumab

Administration may be delayed to assess or treat adverse events, such as cardiac adverseevents or myelosuppression as below. In the neo-adjuvant setting (Cycles 1-6), a dose delay of up to two weeks will be permitted to allow recovery to baseline. Thereafter, one further dose delay of up to two weeks will be permitted before the patient is discontinued form the study. From cycles 7-17(18) dose delays are permitted as deemed necessary by the investigator. Trastuzumab and pertuzumab will administered every 3 weeks for 17(18) cycles unless intolerable toxicity or investigator-assessed disease progression, whichever is sooner.

Toxicity related to Pertuzumab and Trastuzumab

Toxicity related to study treatment	Action
1. Non-hematological, Grade 1 or 2 (NCI-CTCAE: excluding cardiac*) toxicity	Continue with study treatment
2. Non-hematological, Grade 3 or 4 (NCI-CTCAE: excluding cardiac*) toxicity	Hold study treatment (all medication in the cycle) until recovery to Grade ≤ 2.<br>Toxicity resolved to Grade ≤ 1 within a maximum of 2 weeks calculated from last administration: Resume study treatment.<br>Toxicity did NOT resolve to Grade ≤ 2 within a maximum of 2 weeks calculated from last administration: Discontinue the related study medication (pertuzumab or trastuzumab) permanently. Continue treatment as deemed suitable by local investigator.
3. Recurrence of non-hematological, Grade 3 or 4 (NCI-CTCAE: excluding cardiac*) toxicity upon re-challenge	Discontinue the related study medication (pertuzumab or trastuzumab) permanently. Continue treatment as deemed suitable by local investigator.
4. Cardiac toxicity (asymptomatic drop in LVEF or symptomatic congestive heart failure)	Study treatment (all medication in the cycle) to be held, continued or resumed according to the algorithm depicted<br>Related study medication (pertuzumab or trastuzumab) to be discontinued permanently in case of symptomatic CHF
5. Cardiac toxicity (NCI-CTCAE: other cardiac toxicities not covered by treatment algorithm	Actions must follow rules 1. to 3. for non-hematological toxicities
6. Hematological toxicity – Neutropenia	Hold study treatment (all medication in the cycle) until neutrophils ≥ 1.5 x 10^9/L.

11.4 Trastuzumab Emtansine

Trastuzumab Emtansine may be started based on investigator's discretion in case ANC ≥ 1.0X10^9/L or platelet ≥ 100 x 10^9/L at Adjuvant C1D1. Appendix 6 provided adverse event management guideline after Trastuzumab emtansine treatment.

If trastuzumab emtansine is held for toxicity, then atezolizumab must also be held. If trastuzumab emtansine is discontinued for toxicity, atezolizumab must not be continued as single agent and must either be discontinued also or may be continued in combination with trastuzumab and pertuzumab to complete a total of 1 year of HER2-targeted therapy if the investigator considers the toxicity to be related to trastuzumab emtansine without compromising the continued use of trastuzumab. If treatment with atezolizumab had been discontinued prior to the discontinuation of trastuzumab emtansine or if both treatments are discontinued concurrently, it is at the investigator's discretion to switch treatment to trastuzumab and pertuzumab if the toxicity is not considered related to the trastuzumab component of trastuzumab emtansine.

If study treatment is temporarily interrupted because of toxicity caused by trastuzumab emtansine or atezolizumab, the treatment cycles will be restarted such that the atezolizumab and trastuzumab emtansine infusions remain synchronized. If significant trastuzumab emtansine related toxicities have not recovered to Grade 1 or baseline, the next scheduled dose may be delayed for up to 42 days after the last dose was received.

Dose modification scheme for Trastuzumab Emtansine

Dose Reduction Schedule	Dose Level (mg/kg, Q3W)
Starting dose	3.6
First dose reduction	3.0
Second dose reduction	2.4
Requirement for further dose reduction	Discontinue treatment

Q3W＝every 3 weeks.

Note: The dose of trastuzumab emtansine, once reduced, may not be re-escalated. A maximum of two dose reductions is allowed; patients with any further requirement for dose reduction will discontinue treatment with trastuzumab emtansine and atezolizumab/placebo.

12. Statistical analysis

12.1 Primary and secondary endpoints

12.1.1 Primary endpoint

Pathologic complete response rate (pCR) after neoadjuvant pertuzumab, atezolizumab,

docetaxel and Herceptin sc in HER2-positive early breast cancer patients

12.1.2 Secondary endpoint

To evaluate the EFS of atezolizumab add-on adjuvant treatments with Herceptin SC plus pertuzumab in patients with Breast Cancer of patients with pCR versus non-pCR

To assess the efficacy of atezolizumab add-on adjuvant treatments according to PD-L1 status by IHC (IC0 and IC1/2/3 subgroup analysis)

**Objectives**	**Corresponding Endpoints**
Primary Objective:	
To evaluate the pCR rate of TAHP neoadjuvant chemotherapy with the patients with HER2+ EBC	Pathologic complete response rate (pCR)
Secondary Objective:	
➢ To evaluate the EFS of atezolizumab add-on adjuvant treatments with trastuzumab SC plus pertuzumab in patients with Breast Cancer of patients with pCR versus non-pCR<br>➢ To assess the efficacy of atezolizumab add-on adjuvant treatments according to PD-L1 status by IHC(IC0 and IC1/2/3 subgroup analysis)	➢ 3-year Event free survival (EFS) of the patients with pCR vs. non-pCR to TAHP<br>➢ Overall survival (OS)<br>➢ Disease Free Survival (DFS)<br>➢ Pcr and EFS based on PD-L1 status<br>➢ Quality of Life evaluated by FACT-T questionnaire

Safety Objectives	
To determine the safety and tolerability of atezolizumab add-on adjuvant treatments with trastuzumab SC plus pertuzumab for remaining 1 year in patients with Breast Cancer	➢ Safety and Toxicity using NCI-CTCAE version 5.0
Exploratory Biomarker Objectives:	
1. To evaluate efficacy of Neo-adjuvant TAHP chemotherapy based on HER2 amplification (level: high-low)<br><br>2. To assess the role of PI3K on resistance of HER2 positive breast cancer patients<br><br>3. To evaluate correlation between TMB and clinical outcome or any genetic environmental change to response to therapy.	➢ Multi-omic profiling using F1CDx and WTS platform in house<br>➢ Immune signatures such as PD-L1 expression, tumoral mutation burden (TMB), Intrinsic subtyping, mutation signature, pathway analysis including PIK3CA and HER<br>➢ Single cell sequencing of residual cancer after neoadjuvant PATH<br>➢ Cf DNA profiling

12.2 Definition of Analysis Populations

Intent-to-treat population

All patients receiving any amount of study medication will be included in the intent-to-treat population. Any patient who is assigned a patient number, but does not receive any study medication will not be included. Missing value will be included as this analysis is consistent with the intention to treat(ITT) principle. Full analysis set(FAS) will be conducted if the patient completes the trial prior to conduct pCR.

Safety Population

The safety population includes patients who received at least one cycle of treatment.

12.3 Efficacy and safety analysis

12.3.1 Primary End point: pathologic complete response

Primary endpoint of this study is pathologic complete response rate (pCR rate) after 6cycles of neoadjuvant chemotherapy
The aim of this study was to investigate the pathologic complete response rate (pCR rate) of docetaxel + Herceptin sc + pertuzumab in combination with atezolizumab in HER2-positive breast cancer patients as neoadjuvant chemotherapy compared with docetaxel plus carboplatin plus trastuzumab plus pertuzumab (65% complete remission). Pathologic complete response is defined as ypT0/TisN0Mx. pCR rate will be analyzed after all surgery completion. As this study is single arm design, additional statistical analysis will not be planned.

12.3.2 Event free survival and overall survival

Three year event free survival (EFS) is defined as time since Cycle 1 day 1 in 3 years. Event is defined as any events as follow: disease recurrence regardless of distant and/or local, contralateral breast cancer, other malignancy except breast cancer and any cause of death.

Overall survival (OS) is defined as time from cycle 1 day1 to death from any cause. Kaplan-Meier methodology will be used to estimate OS.

12.3.3 Response evaluation and toxicity evaluation

Response evaluation was based on RECIST version 1.1 and Toxicities were evaluated according to NCI-CTCAE version 5.0 [Appendix 2, 3.] As this study is single arm design, additional statistical analysis will not be required. In case to compare response rate and toxicity evaluation between pCR and non pCR, Fisher’s extract method will be used to compare the difference of both populations.

.

<u>Safety reporting period for Atezolizumab</u>

After initiation of Atezolizumab, all AEs will be reported until 30 days after the last dose of study treatment. Serious adverse events and adverse events of special interest will continue to be reported until 90 days after the last dose of atezolizumab.

Safety reporting period for Pertuzumab,Trastuzumab SC and Trastuzumab Emtansine

AEs are to be monitored continuously during study treatment. All AEs occurring during
the study and until the treatment discontinuation visit 28 days after last study medication are to be recorded; thereafter only drug-related SAEs, cardiac AEs (e.g., heart failure), non–breast-related second primary malignancies, all irrespective of causal relationship, and pregnancies, should continue to be collected.

12.3.4 Quality of Life

Health related quality of life was assessed by FACT-T questionnaire. At screening, and at point of every tumor response evaluation, QoL was assessed. As this study is single arm design, additional statistical analysis will not be required. In case to compare response rate and toxicity evaluation between pCR and non pCR, Fisher's extract method will be used to compare the difference of both populations.

13. Biomarker analysis

13.1 Exploratory biomarker analysis using tissue blocks

Biomarker studies will be performed using tissue blocks collected 1) at the time of diagnosis, 2) at 3weeks after 1st treatment (optional), and 3) at the time of surgery (non-pCR) 4) at progression after surgery (optional).

Genome analysis using the F1CDx panel, whole-exome sequencing and RNASeq will be performed. In addition, HER2 status, PD-1,PD-L1 status, PIK3CA and TMB will be assessed via immunochemical staining.

13.2 Exploratory biomarker analysis using blood

Biomarker studies will be done using blood collected 1) at the time of diagnosis, 2) at 3 weeks after first chemotherapy, 3) at the time of surgery and 4) at progression after surgery

At the time of diagnosis, whole exome sequencing and circulating tumor DNA analysis will be performed using blood. In addition, Circulating Nucleic Acid: Circulating Tumor Cell, CTC;

Exosomeanalysis will be performed after 3 weeks of first chemotherapy, at the time of surgery and after disease progression.

13.3 Exploratory biomarker analysis using surgical tissues

Single cell sequencing of patients with residual tumors who did not have complete pathologic remission at the time of surgery will be performed.

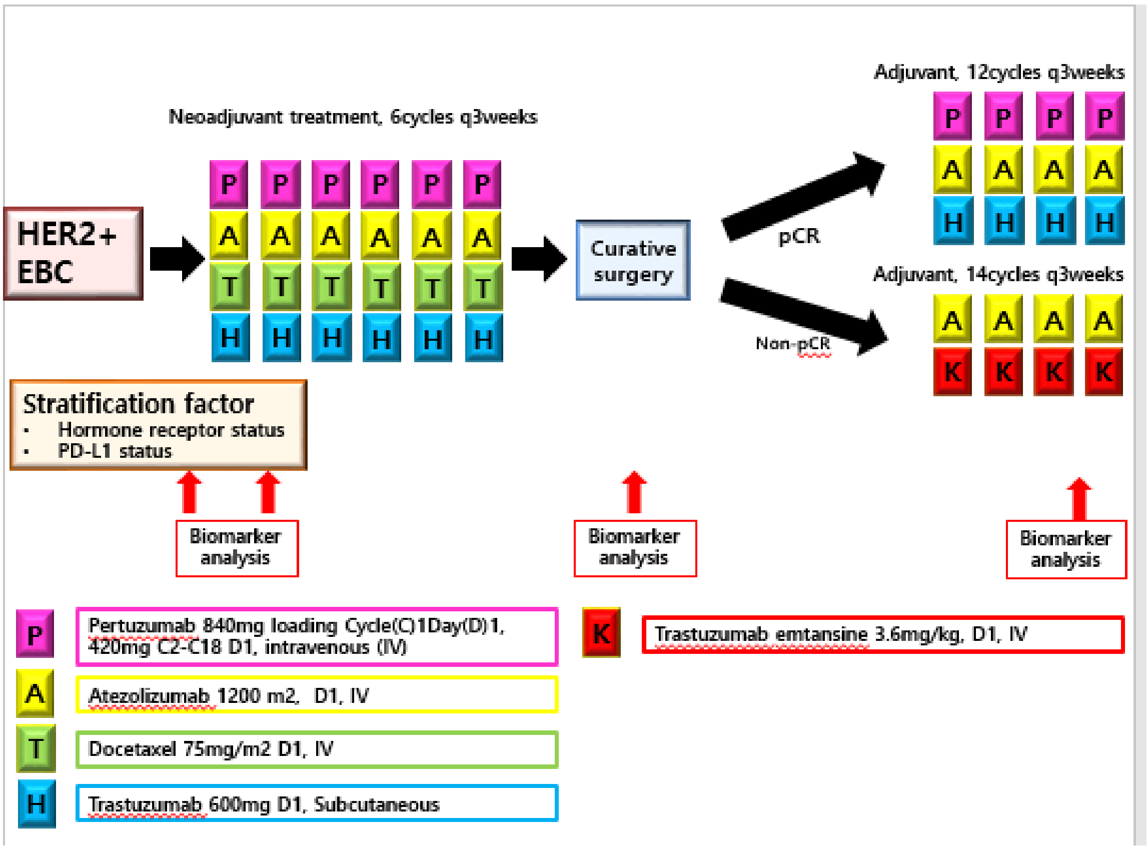

14. Definition and Reporting of Adverse Events

14.1 Definition of adverse events

14.1.1 Adverse events

An AE is any untoward medical occurrence in a patient or clinical investigation patient administered a pharmaceutical product and which does not necessarily have a causal relationship with this treatment. An AE can therefore be any unfavorable and unintended sign [including an abnormal laboratory finding], symptom, or disease temporally associated with the

use of a medicinal [investigational] product, whether or not considered related to the medicinal [investigational] product.

14.1.2 Adverse Drug Reaction

All noxious and unintended responses to a medicinal product related to any dose should be considered adverse drug reactions.

14.2 Adverse Event Report

All adverse events will be reported after initiation of neo-adjuvant therapy. An adverse event occurred prior to study treatment will be recorded as medical history.

After informed consent has been obtained but prior to initiation of neo-adjuvant therapy, only adverse events caused by a protocol-mandated intervention will be reported by investigator's discretion.

AE reports required to collect type of event, duration (onset and end date and time), seriousness, severity, causality and result. All adverse events should be followed up until the event has resolved to baseline grade or better, the event is assessed by stable by the investigator or the patient is lost to follow up.

All AE/SAE will be reported up until safety follow up visit (28 days after last treatment), after then the event will be follow up until the event has resolved to baseline grade or better, the event is assessed as stable by the investigator or the patient is lost to follow up. An adverse event occurred prior to study treatment will be recorded as medical history.

14.3 Serious Adverse Events

It is any Adverse Event that at any dose fulfils at least one of the following criteria:
– is fatal; (results in death; NOTE: death is an outcome, not an event)
– is Life-Threatening (NOTE: the term "Life-Threatening" refers to an event in which the patient was at immediate risk of death at the time of the event; it does not refer to an event which could hypothetically have caused a death had it been more severe).

– required in-patient hospitalization or prolongation of existing hospitalization;

– results in persistent or significant disability/incapacity;

– is a congenital anomaly/birth defect;

– is medically significant or requires intervention to prevent one or other of the outcomes listed above

All definition for the adverse event is originated from Korea Good Clinical Practice.

14.3.1 Serious Adverse Event Reporting

Regardless of causality, all SAEs are required to be reported by the investigator to the IIS Sponsor and supporting companies immediately (no more than 24 hours after learning of the events) via e-amil or fax.

All adverse events (related and unrelated) occurring during the study and up to 28 days after the last dose of study medication must be reported on the eCRF. SAE report will be initiated after starting the first treatment. Regardless of causality, all SAEs will be reported until safety follow up visit (28 days from the last treatment) or until withdrawal from the study, whichever occurs first.

The following is a list of events must report to the IIS Sponsor and supporting companies within 24 hours after learning of the event, regardless of relationship to study treatment;

Serious adverse events

Adverse Events of Special Interest (AESI)

Pregnancies

Investigators must also comply with local requirements for reporting serious adverse events to the local IRB/EC.

REPORTING REQUIREMENTS FROM INVESTIGATOR TO IIS Sponsor and ROCHE

Certain events require reporting to allow the Roche to take appropriate measures to address potential new risks in a clinical study. The investigator must report such events to the Roche. The following is a list of events that the investigator must report to the Roche, regardless of relationship to study drug:

• Serious Adverse Drug Reactions: within 15 calendar days

• Other Serious Adverse Events/Pregnancy: within 30 calendar days

• Adverse events of special interest (related to Roche product) : within 15 calendar days

• Non-serious events: As defined in SDEA

14.3.2 Suspected Unexpected Serious Adverse Reaction Report

IIS sponsor must comply for reporting SUSAR to relevant investigators, IRB and MFDS. MFDS reporting timeline is defined as below;

- Life threatening and/or results in death must be reported within 7 calendar days. Details should be reported within 15 days from the initial report.
- If not life threatening or does not result in death must be submitted to the regulatory authorities within 15 calendar days.

14.3.3 Reporting of study specific AEs and SAEs

Congestive Heart Failure and Symptomatic Left Ventricular Systolic Dysfunction : AESI relevant to trastuzumab

Symptomatic left ventricular systolic dysfunction should be reported as congestive heart failure, and not as individual signs and symptoms thereof. Congestive heart failure must be reported as SAE. In the eCRF, signs and symptoms should be entered in the comments part of the adverse events eform. Congestive heart failure should be graded according to NCI CTCAE v5.0 for "left ventricular systolic dysfunction" and the New York Heart Association (NYHA) classification. Congestive heart failure occurring during the study and up to 24 months after last administration of study medications must be reported irrespective of causal relationship.

Asymptomatic Left Ventricular Systolic Dysfunction : AESI relevant to pertuzumab

Asymptomatic declines in LVEF should not be reported as adverse events since LVEF data are collected separately in the eCRF. Exceptions to this rule are as follows:

• An asymptomatic decline in LVEF ≥ 10%-points from baseline to a LVEF < 50% must be reported as adverse event

• An asymptomatic decline in LVEF requiring treatment or leading to discontinuation of pertuzumab and trastuzumab in the neo-adjuvant part or trastuzumab in the adjuvant part must

be reported in an expedited manner by using the SAE form and classifying the event as Non-Serious Event of Special Interest.

In both cases, it should be reported as left ventricular systolic dysfunction and graded according to NCI CTCAE v5.0.

The following table summarizes the reporting conventions for left ventricular systolic dysfunction:

Observation	How to report	Term to be reported	Grading
Asymptomatic decline in LVEF ≥ 10%-points from baseline to a LVEF < 50%	AE (eCRF AE eform)	Left ventricular systolic dysfunction	NCI CTCAE for "left ventricular systolic dysfunction"
Asymptomatic decline in LVEF requiring treatment or leading to discontinuation of pertuzumab/placebo and trastuzumab	AE (eCRF AE eform) and Non-Serious Event of Special interest (SAE form)	Left ventricular systolic dysfunction	NCI CTCAE for "left ventricular systolic dysfunction"
Symptomatic left ventricular systolic dysfunction	AE (eCRF AE eform) and SAE (SAE form)	Congestive heart failure	NCI CTCAE for "left ventricular systolic dysfunction" and NYHA

AESI relevant to atezolizumab

Autoimmune Hemolytic Anemia

Cytokine-release syndrome

Grade ≥ 2 cardiac disorders (e.g., atrial fibrillation, myocarditis, pericarditis)

Hypersensitivity

Immune-related adrenal insufficience

Immune-related collitis

Immune-related diabetes mellitus

Immune-related Guillain-Barre syndrome

Immune-related hepatitis including AST or ALT > 10 * ULN

Immune-related hyperthyroidism

Immune-related hypophysitis

Immune-related meningoencephalitis

Immune-related Myasthenic syndrome/ Myasthenia Gravis

Immune-related myocarditis,

Immune-related myositis

Immune-related nephritis

Immune-related ocular inflammatory toxicity
Immune-related pancreatitis
Immune-related severe cutaneous reaction
Immune-related vasculitis
Immune –related pneumonitis
influenza-like illness
Infusion-related reactions
Rhabdomyolysis
Systemic immune activation
Systemic inflammatory response syndrome
Systemic lupus erythematosus

14.4 Drug-adverse Event Relationship

The causality relationship of study drug to the adverse event will be assessed by the investigator as either:

-Certain: Event or laboratory test abnormality, with plausible time relationship to drug intake. Cannot be explained by disease or other drugs. Response to withdrawal plausible (clinically). Rechallenge satisfactory, if necessary.

-Probable/likely: Event or laboratory test abnormality, with reasonable time relationship to drug intake. Unlikely to be attributed to disease or other drugs. Response to withdrawal clinically reasonable. Rechallenge not required.

-Possible: Event or laboratory test abnormality, with reasonable time relationship to drug intake. Could also be explained by disease or other drugs. Information on drug withdrawal may be lacking or unclear.

-Unlikely: Event or laboratory test abnormality with a time to drug intake that makes a relationship improable (but not impossible). Disease or other drugs provide plausible explanations.

-Conditional/unclassified: Event or laboratory test abnormality. More data for proper assessment needed, or additional data under examination.

-Unassessable/Unclassifiable: Report suggesting an adverse reaction. Cannot be judged because information is insufficient or contradictory. Data cannot be supplemented or verified.

-Not Related: Clinical events that are not time-related with drugs and are clearly associated with other more likely causes.

15. Withdrawal from the study

15.1 Study Discontinuation

The principal investigator and coordinating investigator have the right to terminate this study for the following reasons:

- The incidence or severity of adverse events in this studies indicates a potential health hazard to patients
- Coordinating investigator decides to withhold or terminate the study.

15.2 Early treatment completion

-Disease progress or relapse confirmation

-In case of uncontrolled toxicities

-Pregnancy

Treatment completion is not meant to withdrawal from the study. Patients need to maintain the study and follow up survival status as per 10.1 schedule of assessment.

15.3 Patient withdrawal from study

Patients have the right to voluntarily withdraw from the study with the following reasons:

-Patient withdrawal of consent at any time

-Any medical condition that the investigator or Sponsor determines may jeopardize the patient's safety if he or she continues in the study

15.3 Subject replacement

Subject replacement is not planned for this study.

16. Emergent process

During the study enrollment and treatment completion, investigator and/or investigational site should be checked all trial related adverse events and abnormal laboratory results are treated appropriately. Investigator and/or investigational site need to inform to the patients in case of any recognized disease if and any medical treatment is required.

17. Ethics and general study administration

17.1 Local regulations/ Declaration of helsinki

The investigator will ensure that this study is conducted in full conformance with the principles of the “Declaration of Helsinki” or with the laws and regulations of the country in which the research is conducted, whichever affords the greater protection to the individual. The study must fully adhere to the principles outlined in “Guideline for Good Clinical Practice” ICH Tripartite Guideline [January 1997] or with local law if it affords greater protection to the patient.

17.2 Informed consent

It is the responsibility of the investigator, or a person designated by the investigator [if acceptable by local regulations], to obtain written informed consent from each patient participating in this study, after adequate explanation of the aims, methods, anticipated benefits, and potential hazards of the study. For patients not qualified or incapable of giving legal consent, written consent must be obtained from the legally acceptable representative. In the case where both the patient and his/her legally acceptable representative are unable to read, an impartial witness should be present during the entire informed consent discussion. After the patient and representative have orally consented to participation in the trial, the witness' signature on the form will attest that the information in the consent form was accurately explained and understood. The investigator or designee must also explain that the patients are completely free to refuse to enter the study or to withdraw from it at any time, for any reason. If new safety information results in significant changes in the risk/benefit assessment, the consent form should be reviewed and updated if necessary. All patients [including those already being treated] should be informed of the new information, given a copy of the revised form and give their consent to continue in the study.

17.3 Confidentiality of trial documents and patient records

The investigator must assure that patients' anonymity will be maintained and that their identities are protected from unauthorized parties. On eCRFs or other documents submitted to the sponsor, patients should not be identified by their names, but by an identification code. The investigator should keep a patient enrollment log showing codes, names and addresses. The investigator should maintain documents not for submission to
sponsor, e.g., patients' written consent forms, in strict confidence.

17.4 Conditions for modifying the protocol

Protocol modifications to ongoing studies must be made only after consultation between an appropriate representative of the sponsor and the investigator [investigator representative[s] in the case of a multicenter trial]. Protocol modifications must be prepared by a representative of the sponsor and initially reviewed and approved by the Clinical Science Leader and Biostatistician.

All protocol modifications must be submitted to the appropriate Independent Ethics Committee or Institutional Review Board for information and approval in accordance with local requirements, and to Regulatory Agencies if required. Approval must be awaited before any changes can be implemented, except for changes necessary to eliminate an immediate hazard to trial patients, or when the change[s] involves only logistical or administrative aspects of the trial [e.g. change in monitor[s], change of telephone number[s].

17.5 Monitoring

It is understood that the responsible monitor [or designee] will contact and visit the investigator regularly and will be allowed, on request, to inspect the various records of the trial [eCRFs and other pertinent data] provided that patient confidentiality is maintained in accord with local requirements.

It will be the monitor's responsibility to inspect the eCRFs at regular intervals throughout the study, to verify the adherence to the protocol and the completeness, consistency and accuracy of the data being entered on them. The monitor should have access to laboratory test reports and other patient records needed to verify the entries on the eCRF. The investigator [or his/her deputy] agrees to cooperate with the monitor to ensure that any problems detected in the course of these monitoring visits are resolved.

17.6 Criteria for Premature Withdrawal

Subjects have the right to withdraw from the study at any time for any reason. The investigator also has the right to withdraw subjects from the study in the event of intercurrent illness, adverse events, and treatment failure after a prescribed procedure, protocol violation, cure, administrative reasons or for other reasons. An excessive rate of withdrawals can render the study uninterpretable; therefore, unnecessary withdrawal of subjects should be avoided. Should a subject decide to withdraw, all efforts will be made to complete and report the observations as thoroughly as possible.

The investigator should contact the subject or a responsible relative by telephone or through a

personal visit to establish as completely as possible the reason for the withdrawal. A complete final evaluation at the time of the subject's withdrawal should be made with an explanation of why the subject is withdrawing from the study. If the reason for removal of a subject from the study is an adverse event the principal specific event will be recorded on the Electronic Case Report Form.
In the case that the subject decides to prematurely discontinue study treatment ["refuses treatment"], he/she should be asked if he/she can still be contacted for further information. The outcome of that discussion should be documented in both the medical records and in the eCRF.

17.7 Investigator's Files / Retention of Documents

The Investigator must maintain adequate and accurate records to enable the conduct of the study to be fully documented and the study data to be subsequently verified. These documents should be classified into two different separate categories. Investigator's Study File, and patient clinical source documents. The Investigator's Study File will contain the protocol/amendments, Independent Ethics Committee/Institutional Review Board and governmental approval with correspondence, sample informed consent, drug records, staff curriculum vitae and authorization forms and other appropriate documents/correspondence etc. In addition at the end of the study the investigator will receive the patient data, which includes an audit trail containing a complete record of all changes to data, query resolution correspondence and reasons for changes, in human readable format on CD which also has to be kept with the Investigator's Study File.

Patient clinical source documents [usually defined by the project in advance to record key efficacy/safety parameters independent of the eCRFs] would include patient hospital/clinic records, physician's and nurse's notes, appointment book, original laboratory reports, ECG, EEG, X-ray, pathology and special assessment reports, signed informed consent forms, consultant letters, and patient screening and enrollment logs. The Investigator must keep these two categories of documents (including the archival CD) on file for at least 15 years after completion or discontinuation of the study. After that period of time the documents may be destroyed, patient to local regulations. Should the Investigator wish to assign the study records to another party or move them to another location, Roche must be notified in advance. If the Investigator cannot guarantee this archiving requirement at the investigational site for any or all of the documents, special arrangements must be made between the Investigator and Roche to store these in a sealed container[s] outside of the site so that they can be returned sealed to the

Investigator in case of a regulatory audit. Where source documents are required for the continued care of the patient, appropriate copies should be made for storing outside of the site.

18. References

1. Cortazar P, Zhang L, Untch M, et al. Pathological complete response and long-term clinical benefit in breast cancer: the CTNeoBC pooled analysis. *Lancet.* 2014;384(9938):164-172.
2. Broglio KR, Quintana M, Foster M, et al. Association of Pathologic Complete Response to Neoadjuvant Therapy in HER2-Positive Breast Cancer With Long-Term Outcomes: A Meta-Analysis. *JAMA Oncol.* 2016;2(6):751-760.
3. Schneeweiss A, Chia S, Hickish T, et al. Pertuzumab plus trastuzumab in combination with standard neoadjuvant anthracycline-containing and anthracycline-free chemotherapy regimens in patients with HER2-positive early breast cancer: a randomized phase II cardiac safety study (TRYPHAENA). *Ann Oncol.* 2013;24(9):2278-2284.
4. Schneeweiss A, Chia S, Hickish T, et al. Long-term efficacy analysis of the randomised, phase II TRYPHAENA cardiac safety study: Evaluating pertuzumab and trastuzumab plus standard neoadjuvant anthracycline-containing and anthracycline-free chemotherapy regimens in patients with HER2-positive early breast cancer. *Eur J Cancer.* 2018;89:27-35.
5. Gianni L, Pienkowski T, Im YH, et al. Efficacy and safety of neoadjuvant pertuzumab and trastuzumab in women with locally advanced, inflammatory, or early HER2-positive breast cancer (NeoSphere): a randomised multicentre, open-label, phase 2 trial. *Lancet Oncol.* 2012;13(1):25-32.
6. Gianni L, Pienkowski T, Im YH, et al. 5-year analysis of neoadjuvant pertuzumab and trastuzumab in patients with locally advanced, inflammatory, or early-stage HER2-positive breast cancer (NeoSphere): a multicentre, open-label, phase 2 randomised trial. *Lancet Oncol.* 2016;17(6):791-800.
7. Swain SM, Ewer MS, Viale G, et al. Pertuzumab, trastuzumab, and standard anthracycline- and taxane-based chemotherapy for the neoadjuvant treatment of patients with HER2-positive localized breast cancer (BERENICE): a phase II, open-label, multicenter, multinational cardiac safety study. *Ann Oncol.* 2018;29(3):646-653.
8. Senkus E, Kyriakides S, Ohno S, et al. Primary breast cancer: ESMO clinical practice guidelines for diagnosis, treatment and follow-up. Ann Oncol 2015;26(Suppl 5):v8-30.
9. [NCCN] National Comprehensive Cancer Network. NCCN guidelines for breast cancer V.3.2017 [resource on the internet] Meeting: 3 and 4 August 2017 [cited 31 May 2018]. Available from: https://www.nccn.org/Common/FileManager.ashx?fileManagerId=e3469a10-d7ad-4f79-a432-fd2d5ecf74a2.

10. Gradishar WJ, Anderson BO, Balassanian R, et al. National Comprehensive Cancer Network. Breast Cancer (Version 1.2017). Available from: https://www.nccn.org/professionals/physician_gls/pdf/breast.pdf.
11. Hammond ME, Hayes DF, Dowsett M, et al. American Society of Clinical Oncology/College of American Pathologists guideline recommendations for immunohistochemical testing of estrogen and progesterone receptors in breast cancer. J Clin Oncol 2010;28:2784-95.
12. Gianni L, Pienkowski T, Im YH, et al. Efficacy and safety of neoadjuvant pertuzumab and trastuzumab in women with locally advanced, inflammatory, or early HER2-positive breast cancer (NeoSphere): a randomised multicentre, open-label, phase 2 trial. Lancet Oncol 2012;13:25-32.
13. Schneeweiss A, Chia S, Hickish T, et al. Pertuzumab plus trastuzumab in combination with standard neoadjuvant anthracycline-containing and anthracycline-free chemotherapy regimens in patients with HER2-positive early breast cancer: a randomized phase II cardiac safety study (TRYPHAENA). Ann Oncol 2013;24:2278-84
14. Lee HJ, Kim YA, Sim CK, et al. Expansion of tumor-infiltrating lymphocytes and their potential for application as adoptive cell transfer therapy in human breast cancer. Oncotarget 2017;8:113345-59.
15. Stagg J, Loi S, Divisekera U, et al. Anti–ErbB-2 mAb therapy requires type I and II interferons and synergizes with anti–PD-1 or anti-CD137 mAb therapy. Proc Natl Acad Sci USA 2011;108:7142-7.
16. Untch M, Jackisch C, Schneeweiss A, et al. Nab-paclitaxel versus solvent-based paclitaxel in neoadjuvant chemotherapy for early breast cancer (GeparSepto-GBG69): a randomised, phase 3 trial. Lancet Oncol 2016;17:345-56.
17. van Ramshorst MS, van Werkhoven E, Mandjes IAM, et al. Trastuzumab in combination with weekly paclitaxel and carboplatin as neo-adjuvant treatment for HER2-positive breast cancer: the TRAIN-study. Eur J Cancer 2017;74:47-54.
18. von Minckwitz G, Procter M, de Azambuja E, et al. Adjuvant pertuzumab and trastuzumab in early HER2-positive breast cancer. N Engl J Med 2017;377:122-31.

19. Appendicies

19.1 Appendix 1. Performance Status Criteria

ECOG (Zubrod)		Karnofsky	
Score	Description	Score	Description
0	Fully active, able to carry on all pre-disease performance without restriction	100	No complains, no evidence of disease
		90	Able to carry on normal activity; minor signs or symptoms of disease
1	Restricted in physically strenuous activity but ambulatory and able to carry out work of a light or sedentary nature, e.g., light house work, office work	80	Some signs or symptoms of disease with effort
		70	Cares for self; unable to carry on normal activity or to do active work
2	Ambulatory and capable of all self-care but unable to carry out any work activities. Up and about more than 50% of waking hours	60	Requires occasional assistance but is able to care for most personal needs
		50	Requires considerable assistance and frequent medical care
3	Capable of only limited self-care, confined to bed or chair more than 50% of waking hours	40	Disabled; requires special care and assistance
		30	Severely disabled; hospitalization indicated although death not imminent
4	Completely disabled. Cannot carry on any self-care. Totally confined to bed or chair	20	Very sick; hospitalization necessary; requires active supportive treatment
		10	Morbid; fatal processes progressing rapidly

19.2 Appendix 2. RECIST 1.1 Criteria for Response

■ **Definition of evaluation of target lesions**

CR	Disappearance; confirmed at 4 wks
PR	At least 30% decrease in the sum of the LD of target lesions, taking as reference the baseline sum LD; confirmed at 4 wks
SD	Neither PR nor PD criteria met
PD	At least 20% increase in the sum of the LD of target lesions, taking as the smallest sum LD recorded since the treatment started or the appearance of one or more new lesions

■ **Definition of evaluation of non-target lesions**

CR	Disappearance and normalization of tumor marker level; confirmed at 4 wks
Incomplete response/SD	Persistence of one or more non-target lesion(s) or/and maintenance of tumor marker level above the normal limits
PD	Appearance of one or more new lesions and/or unequivocal progression of existing non-target lesions

■ **Evaluation of Best Overall Response.**

Target lesions	Non-target lesions	New lesions	Overall response
CR	CR	No	CR
CR	Incomplete response/SD	No	PR
PR	Non-PD	No	PR
SD	Non-PD	No	SD
PD	Any	Yes or No	PD
Any	PD	Yes or No	PD
Any	Any	Yes	PD

19.3 Appendix 3. CTCAE(Common Terminology Criteria for Adverse Events v5.0)

* Only selected criteria are shown in this appendix. Further information can be obtained at https://evs.nci.nih.gov/ftp1/CTCAE/CTCAE_5.0/

19.4 Appendix 4 Risk associated with atezolizumab and guidelines for management of adverse events associated with atezolizumab

Although most immune-related adverse events observed with immunomodulatory agents have been mild and self-limiting, such events should be recognized early and treated promptly to avoid potential major complications. Discontinuation of atezolizumab may not have an immediate therapeutic effect, and in severe cases, immune related toxicities may require acute management with topical corticosteroids, systemic corticosteroids, or other immunosuppressive agents.

The investigator should consider the benefit-risk balance a given patient may be experiencing prior to further administration of atezolizumab. In patients who have met the criteria for permanent discontinuation, resumption of atezolizumab may be considered if the patient is deriving benefit and has fully recovered from the immune related event. Patients can be re-challenged with atezolizumab only after approval has been documented by both the investigator (or an appropriate delegate) and Coordinating investigator.

1) Management Guidelines for Pulmonary Events

Dyspnoea, cough, fatigue, hypoxia, pneumonitis, and pulmonary infiltrates have been associated with the administration of atezolizumab. Patients will be assessed for pulmonary signs and symptoms throughout the study and will also have computed tomography (CT) scans of the chest performed at every tumour assessment.

All pulmonary events should be thoroughly evaluated for other commonly reported aetiologies such as pneumonia or other infection, lymphangitic carcinomatosis, pulmonary embolism, heart failure, chronic obstructive pulmonary disease, or pulmonary hypertension. Management guidelines for pulmonary events are provided in table 1.

Table 1. Management Guidelines for Pulmonary Events, Including Pneumonitis

Event	Management
Pulmonary event, Grade 1	• Continue atezolizumab and monitor closely.<br>• Re-evaluate on serial imaging.<br>• Consider patient referral to pulmonary specialist.
Pulmonary event, Grade 2	• Withhold atezolizumab for up to 12 weeks after event onset. [a]<br>• Refer patient to pulmonary and infectious disease specialists and consider bronchoscopy or BAL.<br>• Initiate treatment with 1–2 mg/kg/day oral prednisone or equivalent.<br>• If event resolves to Grade 1 or better, resume atezolizumab. [b]<br>• If event does not resolve to Grade 1 or better while withholding atezolizumab, permanently discontinue atezolizumab and contact Coordinating investigator. [c]<br>• For recurrent events, treat as a Grade 3 or 4 event.
Pulmonary event, Grade 3	• Permanently discontinue atezolizumab and contact Coordinating investigator. [c]

Event	Management
or 4	• Bronchoscopy or BAL is recommended.<br>• Initiate treatment with 1–2 mg/kg/day oral prednisone or equivalent.<br>• If event does not improve within 48 hours after initiating corticosteroids, consider adding an immunosuppressive agent.<br>• If event resolves to Grade 1 or better, taper corticosteroids over ≥1 month.

BAL = bronchoscopic alveolar lavage.

[a] Atezolizumab may be withheld for a longer period of time (i.e., > 12 weeks after event onset) to allow for corticosteroids (if initiated) to be reduced to ≤ 10 mg/day oral prednisone or equivalent. The acceptable length of the extended period of time must be agreed upon by the investigator and the Coordinating investigator.

[b] If corticosteroids have been initiated, they must be tapered over ≥ 1 month to ≤ 10 mg/day oral prednisone or equivalent before atezolizumab can be resumed.

[c] Resumption of atezolizumab may be considered in patients who are deriving benefit and have fully recovered from the immune-related event. Patients can be re-challenged with atezolizumab only after approval has been documented by both the investigator (or an appropriate delegate) and the Coordinating investigator.

2) Management Guidelines for Hepatic Events

Immune-related hepatitis has been associated with the administration of atezolizumab.—Eligible patients must have adequate liver function, as manifested by measurements of total bilirubin and hepatic transaminases, and liver function will be monitored throughout study treatment. Management guidelines for hepatic events are provided in Table 2.

Patients with right upper-quadrant abdominal pain and/or unexplained nausea or vomiting should have liver function tests (LFTs) performed immediately and reviewed before administration of the next dose of study drug.

For patients with elevated LFTs, concurrent medication, viral hepatitis, and toxic or neoplastic aetiologies should be considered and addressed, as appropriate.

Table 2. Management Guidelines for Hepatic Events

Event	Management
Hepatic event, Grade 1	• Continue atezolizumab.<br>• Monitor LFTs until values resolve to within normal limits.
Hepatic event, Grade 2	**All events:**<br>• Monitor LFTs more frequently until return to baseline values.<br>**Events of > 5 days' duration:**<br>• Withhold atezolizumab for up to 12 weeks after event onset. [a]<br>• Initiate treatment with 1–2 mg/kg/day oral prednisone or equivalent.<br>• If event resolves to Grade 1 or better, resume atezolizumab. [b]<br>• If event does not resolve to Grade 1 or better while withholding atezolizumab, permanently discontinue atezolizumab and contact Coordinating investigator. [c]

Hepatic event, Grade 3 or 4	• Permanently discontinue atezolizumab and contact Coordinating investigator. [c]<br>• Consider patient referral to gastrointestinal specialist for evaluation and liver biopsy to establish aetiology of hepatic injury.<br>• Initiate treatment with 1–2 mg/kg/day oral prednisone or equivalent.<br>• If event does not improve within 48 hours after initiating corticosteroids, consider adding an immunosuppressive agent.<br>If event resolves to Grade 1 or better, taper corticosteroids over ≥ 1 month.

LFT = liver function tests.

[a] Atezolizumab may be withheld for a longer period of time (i.e., > 12 weeks after event onset) to allow for corticosteroids (if initiated) to be reduced to ≤ 10 mg/day oral prednisone or equivalent. The acceptable length of the extended period of time must be agreed upon by the investigator and the Coordinating investigator.

[b] If corticosteroids have been initiated, they must be tapered over ≥ 1 month to ≤ 10 mg/day oral prednisone or equivalent before atezolizumab can be resumed.

[c] Resumption of atezolizumab may be considered in patients who are deriving benefit and have fully recovered from the immune-related event. Patients can be re-challenged with atezolizumab only after approval has been documented by both the investigator (or an appropriate delegate) and the Coordinating investigator.

3) Management Guidelines for Gastrointestinal Events

Immune-related colitis has been associated with the administration of atezolizumab. Management guidelines for diarrhoea or colitis are provided in Table 3.

All events of diarrhoea or colitis should be thoroughly evaluated for other more common aetiologies. For events of significant duration or magnitude or associated with signs of systemic inflammation or acute-phase reactants (e.g., increased C-reactive protein, platelet count, or bandemia): Perform sigmoidoscopy (or colonoscopy, if appropriate) with colonic biopsy, with three to five specimens for standard paraffin block to check for inflammation and lymphocytic infiltrates to confirm colitis diagnosis.

Table 3. Management Guidelines for Gastrointestinal Events (Diarrhoea or Colitis)

Event	Management
Diarrhoea or colitis, Grade 1	• Continue atezolizumab.<br>• Initiate symptomatic treatment.<br>• Endoscopy is recommended if symptoms persist for > 7 days.<br>• Monitor closely.
Diarrhoea or colitis, Grade 2	• Withhold atezolizumab for up to 12 weeks after event onset. [a]<br>• Initiate symptomatic treatment.<br>• Patient referral to GI specialist is recommended.<br>• For recurrent events or events that persist > 5 days, initiate treatment with 1–2 mg/kg/day oral prednisone or equivalent.<br>• If event resolves to Grade 1 or better, resume atezolizumab. [b]

	• If event does not resolve to Grade 1 or better while withholding atezolizumab, permanently discontinue atezolizumab and contact Coordinating investigator. [c]
Diarrhoea or colitis, Grade 3	• Withhold atezolizumab for up to 12 weeks after event onset. [a]<br>• Refer patient to GI specialist for evaluation and confirmatory biopsy.<br>• Initiate treatment with 1–2 mg/kg/day IV methylprednisolone or equivalent and convert to 1–2 mg/kg/day oral prednisone or equivalent upon improvement.<br>• If event resolves to Grade 1 or better, resume atezolizumab. [b]<br>• If event does not resolve to Grade 1 or better while withholding atezolizumab, permanently discontinue atezolizumab and contact Coordinating investigator. [c]
Diarrhoea or colitis, Grade 4	• Permanently discontinue atezolizumab and contact Coordinating investigator. [c]<br>• Refer patient to GI specialist for evaluation and confirmation biopsy.<br>• Initiate treatment with 1–2 mg/kg/day IV methylprednisolone or equivalent and convert to 1–2 mg/kg/day oral prednisone or equivalent upon improvement.<br>• If event does not improve within 48 hours after initiating corticosteroids, consider adding an immunosuppressive agent.<br>• If event resolves to Grade 1 or better, taper corticosteroids over ≥1 month.

GI = gastrointestinal.

[a] Atezolizumab may be withheld for a longer period of time (i.e., > 12 weeks after event onset) to allow for corticosteroids (if initiated) to be reduced to ≤ 10 mg/day oral prednisone or equivalent. The acceptable length of the extended period of time must be agreed upon by the investigator and the Coordinating investigator.

[b] If corticosteroids have been initiated, they must be tapered over ≥ 1 month to ≤ 10 mg/day oral prednisone or equivalent before atezolizumab can be resumed.

[c] Resumption of atezolizumab may be considered in patients who are deriving benefit and have fully recovered from the immune-related event. Patients can be re-challenged with atezolizumab only after approval has been documented by both the investigator (or an appropriate delegate) and the Coordinating investigator.

4) Management Guidelines for Endocrine Events

Thyroid disorders, adrenal insufficiency, diabetes mellitus, and pituitary disorders have been associated with the administration of atezolizumab. Management guidelines for endocrine events are provided in Table 4.

Patients with unexplained symptoms such as headache, fatigue, myalgias, impotence, constipation, or mental status changes should be investigated for the presence of thyroid, pituitary, or adrenal endocrinopathies. The patient should be referred to an endocrinologist if an endocrinopathy is suspected. Thyroid-stimulating hormone (TSH) and free triiodothyronine and thyroxine levels should be measured to determine whether thyroid abnormalities are present. Pituitary hormone levels and function tests (e.g., TSH, growth hormone, luteinizing hormone, follicle-stimulating hormone, testosterone, prolactin, adrenocorticotropic hormone [ACTH] levels,

and ACTH stimulation test) and magnetic resonance imaging (MRI) of the brain (with detailed pituitary sections) may help to differentiate primary pituitary insufficiency from primary adrenal insufficiency.

Table 4. Management Guidelines for Endocrine Events

Event	Management
Asymptomatic hypothyroidism	• Continue atezolizumab.<br>• Initiate treatment with thyroid replacement hormone.<br>• Monitor TSH weekly.
Symptomatic hypothyroidism	• Withhold atezolizumab.<br>• Initiate treatment with thyroid replacement hormone.<br>• Monitor TSH weekly.<br>• Consider patient referral to endocrinologist.<br>• Resume atezolizumab when symptoms are controlled and thyroid function is improving.
Asymptomatic hyperthyroidism	**TSH ≥ 0.1 mU/L and < 0.5 mU/L:**<br>• Continue atezolizumab.<br>• Monitor TSH every 4 weeks.<br>**TSH < 0.1 mU/L:**<br>• Follow guidelines for symptomatic hyperthyroidism.
Symptomatic hyperthyroidism	• Withhold atezolizumab.<br>• Initiate treatment with anti-thyroid drug such as methimazole or carbimazole as needed.<br>• Consider patient referral to endocrinologist.<br>• Resume atezolizumab when symptoms are controlled and thyroid function is improving.<br>• Permanently discontinue atezolizumab and contact Coordinating investigator for life-threatening immune-*mediated* hyperthyroidism. [c]
Symptomatic adrenal insufficiency, Grade 2–4	• Withhold atezolizumab for up to 12 weeks after event onset. [a]<br>• Refer patient to endocrinologist.<br>• Perform appropriate imaging.<br>• Initiate treatment with 1–2 mg/kg/day IV methylprednisolone or equivalent and convert to 1–2 mg/kg/day oral prednisone or equivalent upon improvement.<br>• If event resolves to Grade 1 or better and patient is stable on replacement therapy, resume atezolizumab. [b]<br>• If event does not resolve to Grade 1 or better or patient is not stable on replacement therapy while withholding atezolizumab, permanently discontinue atezolizumab and contact Coordinating investigator. [c]

Event	Management
Hyperglycemia, Grade 1 or 2	• Continue atezolizumab.<br>• Investigate for diabetes. If patient has Type 1 diabetes, treat as a Grade 3 event. If patient does not have Type 1 diabetes, treat as per institutional guidelines.<br>• Monitor for glucose control.
Hyperglycemia, Grade 3 or 4	• Withhold atezolizumab.<br>• Initiate treatment with insulin.<br>• Monitor for glucose control.<br>• Resume atezolizumab when symptoms resolve and glucose levels are stable.
Hypophysitis (pan-hypopituitarism), Grade 2 or 3	• Withhold atezolizumab for up to 12 weeks after event onset. [a]<br>• Refer patient to endocrinologist.<br>• Perform brain MRI (pituitary protocol).<br>• Initiate treatment with 1–2 mg/kg/day IV methylprednisolone or equivalent and convert to 1–2 mg/kg/day oral prednisone or equivalent upon improvement.<br>• Initiate hormone replacement if clinically indicated.<br>• If event resolves to Grade 1 or better, resume atezolizumab. [b]<br>• If event does not resolve to Grade 1 or better while withholding atezolizumab, permanently discontinue atezolizumab and contact Coordinating investigator. [c]<br>• For recurrent hypophysitis, treat as a Grade 4 event.
Hypophysitis (pan-hypopituitarism), Grade 4	• Permanently discontinue atezolizumab and contact Coordinating investigator. [c]<br>• Refer patient to endocrinologist.<br>• Perform brain MRI (pituitary protocol).<br>• Initiate treatment with 1–2 mg/kg/day IV methylprednisolone or equivalent and convert to 1–2 mg/kg/day oral prednisone or equivalent upon improvement.<br>• Initiate hormone replacement if clinically indicated.

MRI = magnetic resonance imaging; TSH = thyroid-stimulating hormone.

[a] Atezolizumab may be withheld for a longer period of time (i.e., > 12 weeks after event onset) to allow for corticosteroids (if initiated) to be reduced to ≤ 10 mg/day oral prednisone or equivalent. The acceptable length of the extended period of time must be agreed upon by the investigator and the Coordinating investigator.

[b] If corticosteroids have been initiated, they must be tapered over ≥ 1 month to ≤ 10 mg/day oral prednisone or equivalent before atezolizumab can be resumed.

[c] Resumption of atezolizumab may be considered in patients who are deriving benefit and have fully recovered from the immune-related event. Patients can be re-challenged with atezolizumab only after approval has been documented by both the investigator (or an appropriate delegate) and the Coordinating investigator..

5) Management Guidelines for Ocular Events

An ophthalmologist should evaluate visual complaints (e.g., uveitis, retinal events). Management guidelines for ocular events are provided in table 5

Table 5. Management Guidelines for Ocular Events

Event	Management
Ocular event, Grade 1	• Continue atezolizumab.<br>• Patient referral to ophthalmologist is strongly recommended.<br>• Initiate treatment with topical corticosteroid eye drops and topical immunosuppressive therapy.<br>• If symptoms persist, treat as a Grade 2 event.
Ocular event, Grade 2	• Withhold atezolizumab for up to 12 weeks after event onset. [a]<br>• Patient referral to ophthalmologist is strongly recommended.<br>• Initiate treatment with topical corticosteroid eye drops and topical immunosuppressive therapy.<br>• If event resolves to Grade 1 or better, resume atezolizumab. [b]<br>• If event does not resolve to Grade 1 or better while withholding atezolizumab, permanently discontinue atezolizumab and contact Coordinating investigator. [c]
Ocular event, Grade 3 or 4	• Permanently discontinue atezolizumab and contact Coordinating investigator. [c]<br>• Refer patient to ophthalmologist.<br>• Initiate treatment with 1–2 mg/kg/day oral prednisone or equivalent.<br>• If event resolves to Grade 1 or better, taper corticosteroids over ≥1 month.

6) Management Guidelines for Immune-Related Myocarditis

Immune-related myocarditis has been associated with the administration of atezolizumab. Immune-related myocarditis should be suspected in any patient presenting with signs or symptoms suggestive of myocarditis, including, but not limited to, dyspnoea, chest pain, palpitations, fatigue, decreased exercise tolerance, or syncope. Immune-related myocarditis needs to be distinguished from myocarditis resulting from infection (commonly viral, e.g., in a patient who reports a recent history of gastrointestinal illness), ischemic events, underlying arrhythmias, exacerbation of preexisting cardiac conditions, or progression of malignancy.

All patients with possible myocarditis should be urgently evaluated by performing cardiac enzyme assessment, an ECG, a chest X-ray, an echocardiogram, and a cardiac MRI as appropriate per institutional guidelines. A cardiologist should be consulted. An endomyocardial biopsy may be considered to enable a definitive diagnosis and appropriate treatment, if clinically indicated. Patients with signs and symptoms of myocarditis, in the absence of an identified alternate aetiology, should be treated according to the guidelines in table 6.

Table 6. Management Guidelines for Immune-Related Myocarditis

Event	Management
Immune-related myocarditis, Grade 1	• Refer patient to cardiologist.<br>• Initiate treatment as per institutional guidelines.
Immune-related myocarditis, Grade 2	• Withhold atezolizumab for up to 12 weeks after event onset [a] and contact Coordinating investigator.<br>• Refer patient to cardiologist.<br>• Initiate treatment as per institutional guidelines and consider antiarrhythmic drugs, temporary pacemaker, ECMO, or VAD as appropriate.<br>• Consider treatment with 1–2 mg/kg/day IV methylprednisolone or equivalent and convert to 1–2 mg/kg/day oral prednisone or equivalent upon improvement.[a]<br>• If event resolves to Grade 1 or better, resume atezolizumab. [b]<br>• If event does not resolve to Grade 1 or better while withholding atezolizumab, permanently discontinue atezolizumab and contact Coordinating investigator. [c]
Immune-related myocarditis, Grade 3-4	• Permanently discontinue atezolizumab and contact Coordinating investigator. [c]<br>• Refer patient to cardiologist.<br>• Initiate treatment as per institutional guidelines and consider antiarrhythmic drugs, temporary pacemaker, ECMO, or VAD as appropriate.<br>• Initiate treatment with 1–2 mg/kg/day IV methylprednisolone or equivalent and convert to 1–2 mg/kg/day oral prednisone or equivalent upon improvement. [a,b]<br>• If event does not improve within 48 hours after initiating corticosteroids, consider adding an immunosuppressive agent.<br>• If event resolves to Grade 1 or better, taper corticosteroids over ≥ 1 month.

ECMO = extracorporeal membrane oxygenation; VAD = ventricular assist device.

[a] Atezolizumab may be withheld for a longer period of time (i.e., > 12 weeks after event onset) to allow for corticosteroids (if initiated) to be reduced to ≤ 10 mg/day oral prednisone or equivalent. The acceptable length of the extended period of time must be agreed upon by the investigator and the Coordinating investigator.

[b] If corticosteroids have been initiated, they must be tapered over ≥ 1 month to ≤ 10 mg/day oral prednisone or equivalent before atezolizumab can be resumed.

[c] Resumption of atezolizumab may be considered in patients who are deriving benefit and have fully recovered from the immune-related event. Patients can be re-challenged with atezolizumab only after approval has been documented by both the investigator (or an appropriate delegate) and the Coordinating investigator.

7) Management Guidelines for Infusion-Related Reactions

No premedication is indicated for the administration of Cycle 1 of atezolizumab. However, patients who experience an infusion-related reaction (IRR) with Cycle 1 of atezolizumab may receive premedication with antihistamines or antipyretics/analgesics (e.g., acetaminophen) for subsequent infusions. Metamizole (dipyrone) is prohibited in treating atezolizumab-associated

IRRs because of its potential for causing agranulocytosis.

IRRs are known to occur with the administration of monoclonal antibodies and have been reported with atezolizumab. These reactions, which are thought to be due to release of cytokines and/or other chemical mediators, occur within 24 hours of atezolizumab administration and are generally mild to moderate in severity.

CRS is defined as a supraphysiologic response following administration of any immune therapy that results in activation or engagement of endogenous or infused T cells and/or other immune effector cells. Symptoms can be progressive, always include fever at the onset, and may include hypotension, capillary leak (hypoxia), and end-organ dysfunction (LeeRiegler et al. 2019). CRS has been well documented with chimeric antigen receptor T-cell therapies and bispecific T-cell engager antibody therapies but has also been reported with immunotherapies that target PD-1 or PD-L1 (Rotz et al. 2017; Adashek and Feldman 2019), including atezolizumab.

T here may be significant overlap in signs and symptoms of IRRs and CRS, and in recognition of the challenges in clinically distinguishing between the two, consolidated guidelines for medical management of IRRs and CRS during Cycle 1 are provided in Table 7.

Table 7. Management Guidelines for Infusion-Related Reactions

Event	Management
Grade 1[a]<br>*Fever [b] with or without constitutional symptoms*	• *Immediately interrupt infusion.*<br>• *Upon symptom resolution, wait for 30 minutes and then restart infusion at* half the rate being given at the time of event onset.<br>• If the infusion is tolerated at the reduced rate for 30 minutes, the infusion rate may be increased to the original rate.<br>• *If symptoms recur, discontinue infusion of this dose.*<br>• *Administer symptomatic treatment, [c] including maintenance of IV fluids for hydration.*<br>• *In case of rapid decline or prolonged CRS (>2 days) or in patients with significant symptoms and/or comorbidities, consider managing as per Grade 2.*<br>*For subsequent infusions, consider administration of oral premedication with antihistamines, anti-pyretics, and/or analgesics, and monitor closely for IRRs and/or CRS.*

Event	Management
Grade 2[a] *Fever* [b] *with hypotension not requiring vasopressors* ***and/or*** *Hypoxia requiring low-flow oxygen* [d] *by nasal cannula or blow-by*	• *Immediately i*nterrupt infusion.<br>• Upon symptom resolution, wait for 30 minutes and then restart infusion at half the rate being given at the time of event onset.<br>• *If symptoms recur, discontinue infusion of this dose.*<br>• *Administer symptomatic treatment.* [c]<br>• *For hypotension, administer IV fluid bolus as needed.*<br>• *Monitor cardiopulmonary and other organ function closely (in the ICU, if appropriate). Administer IV fluids as clinically indicated, and manage constitutional symptoms and organ toxicities as per institutional practice.*<br>• *Rule out other inflammatory conditions that can mimic CRS (e.g., sepsis). If no improvement within 24 hours, initiate workup and assess for signs and symptoms of HLH or MAS as described in this appendix.*<br>• *Consider IV corticosteroids (e.g., methylprednisolone 2 mg/kg/day or dexamethasone 10 mg every 6 hours).*<br>• *Consider anti-cytokine therapy.* [e]<br>• *Consider hospitalization until complete resolution of symptoms. If no improvement within 24 hours, manage as per Grade 3, that is, hospitalize patient (monitoring in the ICU is recommended), permanently discontinue atezolizumab, and contact Coordinating investigator.*<br>• *If symptoms resolve to Grade 1 or better for 3 consecutive days, the next dose of atezolizumab may be administered.*<br>• *For subsequent infusions, consider administration of oral premedication with antihistamines, anti-pyretics, and/or analgesics and monitor closely* for IRRs *and/or CRS*.<br>*If symptoms do not resolve to Grade 1 or better for 3 consecutive days, contact Coordinating investigator.*

Event	Management
Grade 3[a]<br>*Fever [b] with hypotension requiring a vasopressor (with or without vasopressin)*<br>***<u>and/or</u>***<br>*Hypoxia requiring high-flow oxygen [d] by nasal cannula, face mask, non-rebreather mask, or Venturi mask*	• Permanently discontinue atezolizumab and contact Coordinating investigator[f].<br>• *Administer symptomatic treatment[c].*<br>• *For hypotension, administer IV fluid bolus and vasopressor as needed.*<br>• *Monitor cardiopulmonary and other organ function closely; monitoring in the ICU is recommended. Administer IV fluids as clinically indicated, and manage constitutional symptoms and organ toxicities as per institutional practice.*<br>• *Rule out other inflammatory conditions that can mimic CRS (e.g., sepsis). If no improvement within 24 hours, initiate workup and assess for signs and symptoms of HLH or MAS as described in this appendix.*<br>• *Administer IV corticosteroids (e.g., methylprednisolone 2 mg/kg/day or dexamethasone 10 mg every 6 hours).*<br>• *Consider anti-cytokine therapy [e].*<br>*Hospitalize patient until complete resolution of symptoms. If no improvement within 24 hours, manage as per Grade 4, that is, admit patient to ICU and initiate hemodynamic monitoring, mechanical ventilation, and/or IV fluids and vasopressors as needed; for patients who are refractory to anti-cytokine therapy, experimental treatments may be considered at the discretion of the investigator and in consultation with the Coordinating investigator.*
<u>Grade 4</u>[a]<br>*Fever [b] with hypotension requiring multiple vasopressors (excluding vasopressin)*<br>***<u>and/or</u>***<br>*Hypoxia requiring oxygen by positive pressure (e.g., CPAP, BiPAP, intubation and mechanical ventilation)*	• *Permanently discontinue atezolizumab and contact Coordinating investigator [f].*<br>• *Administer symptomatic treatment[c].*<br>• *Admit patient to ICU and initiate hemodynamic monitoring, mechanical ventilation, and/or IV fluids and vasopressors as needed. Monitor other organ function closely. Manage constitutional symptoms and organ toxicities as per institutional practice.*<br>• *Rule out other inflammatory conditions that can mimic CRS (e.g., sepsis). If no improvement within 24 hours, initiate workup and assess for signs and symptoms of HLH or MAS as described in this appendix.*<br>• *Administer IV corticosteroids (e.g., methylprednisolone 2 mg/kg/day or dexamethasone 10 mg every 6 hours).*<br>• *Consider anti-cytokine therapy [e]. For patients who are refractory to anti-cytokine therapy, experimental treatments [g] may be considered at the discretion of the investigator and in consultation with the Coordinating investigator.*<br>*Hospitalize patient until complete resolution of symptoms.*

Event	Management

ASTCT = American Society for Transplantation and Cellular Therapy; BiPAP = bi-level positive airway pressure; CAR = chimeric antigen receptor; CPAP = continuous positive airway pressure; CRS = cytokine-release syndrome; CTCAE = Common Terminology Criteria for Adverse Events; eCRF = electronic Case Report Form; HLH=hemophagocytic lymphohistiocytosis; ICU = intensive care unit; IRR = infusion-related reaction *; MAS=macrophage activation syndrome; NCCN = National Cancer Comprehensive Network; NCI = National Cancer Institute.*

[a] *Grading system for management guidelines is based on ASTCT consensus grading for CRS. NCI CTCAE v5.0 should be used when reporting severity of IRRs, CRS, or organ toxicities associated with CRS on the Adverse Event eCRF. Organ toxicities associated with CRS should not influence overall CRS grading.*

[b] *Fever is defined as temperature ≥38°C not attributable to any other cause. In patients who develop CRS and then receive anti-pyretic, anti-cytokine, or corticosteroid therapy, fever is no longer required when subsequently determining event severity (grade). In this case, the grade is driven by the presence of hypotension and/or hypoxia.*

[c] *Symptomatic treatment may include oral or IV antihistamines, anti-pyretics, analgesics, bronchodilators, and/or oxygen. For bronchospasm, urticaria, or dyspnea, additional treatment may be administered as per institutional practice.*

[d] *Low flow is defined as oxygen delivered at ≤ 6 L/min, and high flow is defined as oxygen delivered at > 6 L/min.*

[e] *There are case reports where anti-cytokine therapy has been used for treatment of CRS with immune checkpoint inhibitors (Rotz et al. 2017; Adashek and Feldman 2019), but data are limited, and the role of such treatment in the setting of antibody-associated CRS has not been established.*

[f] Resumption of atezolizumab may be considered in patients who are deriving benefit and have fully recovered from the immune-*mediated* event. Patients can be re-challenged with atezolizumab only after approval has been documented by both the investigator (or an appropriate delegate) and the Coordinating investigator. *For subsequent infusions, administer oral premedication with antihistamines, anti-pyretics, and/or analgesics, and monitor closely for IRRs and/or CRS. Premedication with corticosteroids and extending the infusion time may also be considered after consulting the Coordinating investigator and considering the benefit–risk ratio.*

[g] *Refer to Riegler et al. (2019) for information on experimental treatments for CRS.*

8) Management Guidelines for Pancreatic Events, Including Pancreatitis

Symptoms of abdominal pain associated with elevations of amylase and lipase, suggestive of pancreatitis, have been associated with the administration of atezolizumab. The differential diagnosis of acute abdominal pain should include pancreatitis. Appropriate work-up should include an evaluation for ductal obstruction, as well as serum amylase and lipase tests. Management guidelines for pancreatic events, including pancreatitis, are provided in table 8.

Table 8. Management guidelines for Pancreatic events, including Pancreatitis

Event	Management
Amylase and/or lipase elevation, Grade 2	• Continue atezolizumab.<br>• Monitor amylase and lipase weekly.<br>• For prolonged elevation (e.g., > 3 weeks), consider treatment with 10 mg/day oral prednisone or equivalent.
Amylase and/or lipase elevation, Grade 3 or 4	• Withhold atezolizumab for up to 12 weeks after event onset. [a]<br>• Refer patient to GI specialist.<br>• Monitor amylase and lipase every other day.<br>• If no improvement, consider treatment with 1–2 mg/kg/day oral prednisone or equivalent.<br>• If event resolves to Grade 1 or better, resume atezolizumab. [b]<br>• If event does not resolve to Grade 1 or better while withholding atezolizumab, permanently discontinue atezolizumab and contact Coordinating investigator. [c]<br>• For recurrent events, permanently discontinue atezolizumab and contact Coordinating investigator. [c]
Immune-related pancreatitis, Grade 2 or 3	• Withhold atezolizumab for up to 12 weeks after event onset. [a]<br>• Refer patient to GI specialist.<br>• Initiate treatment with 1–2 mg/kg/day IV methylprednisolone or equivalent and convert to 1–2 mg/kg/day oral prednisone or equivalent upon improvement.<br>• If event resolves to Grade 1 or better, resume atezolizumab. [b]<br>• If event does not resolve to Grade 1 or better while withholding atezolizumab, permanently discontinue atezolizumab and contact Coordinating investigator. [c]<br>• For recurrent events, permanently discontinue atezolizumab and contact Coordinating investigator. [c]
Immune-related pancreatitis, Grade 4	• Permanently discontinue atezolizumab and contact Coordinating investigator. [c]<br>• Refer patient to GI specialist.<br>• Initiate treatment with 1–2 mg/kg/day IV methylprednisolone or equivalent and convert to 1–2 mg/kg/day oral prednisone or equivalent upon improvement.<br>• If event does not improve within 48 hours after initiating corticosteroids, consider adding an immunosuppressive agent.<br>• If event resolves to Grade 1 or better, taper corticosteroids over ≥ 1 month.

GI = gastrointestinal.

[a] Atezolizumab may be withheld for a longer period of time (i.e., > 12 weeks after event onset) to allow for corticosteroids (if initiated) to be reduced to ≤ 10 mg/day oral prednisone or equivalent. The acceptable length of the extended period of time must be agreed upon by the investigator and the Coordinating investigator.

[b] If corticosteroids have been initiated, they must be tapered over ≥ 1 month to ≤ 10 mg/day oral

Event	Management

prednisone or equivalent before atezolizumab can be resumed.

[c] Resumption of atezolizumab may be considered in patients who are deriving benefit and have fully recovered from the immune-related event. Patients can be re-challenged with atezolizumab only after approval has been documented by both the investigator (or an appropriate delegate) and the Coordinating investigator.

9)Management Guidelines for Dermatologic Events

Treatment-emergent rash has been associated with atezolizumab. The majority of cases of rash were mild in severity and self-limited, with or without pruritus. A dermatologist should evaluate persistent and/or severe rash or pruritus. A biopsy should be considered unless contraindicated. Management guidelines for dermatologic events are provided in table 9

Table 9. Management guidelines for Dermatologic Events

Event	Management
Dermatologic event, Grade 1	• Continue atezolizumab.<br>• Consider treatment with topical corticosteroids and/or other symptomatic therapy (e.g., antihistamines).
Dermatologic event, Grade 2	• Continue atezolizumab.<br>• Consider patient referral to dermatologist.<br>• Initiate treatment with topical corticosteroids.<br>• Consider treatment with higher-potency topical corticosteroids if event does not improve.
Dermatologic event, Grade 3	• Withhold atezolizumab for up to 12 weeks after event onset. [a]<br>• Refer patient to dermatologist.<br>• Initiate treatment with 10 mg/day oral prednisone or equivalent, increasing dose to 1–2 mg/kg/day if event does not improve within 48–72 hours.<br>• If event resolves to Grade 1 or better, resume atezolizumab. [b]<br>• If event does not resolve to Grade 1 or better while withholding atezolizumab, permanently discontinue atezolizumab and contact Coordinating investigator. [c]
Dermatologic event, Grade 4	• Permanently discontinue atezolizumab and contact Coordinating investigator. [c]

[a] Atezolizumab may be withheld for a longer period of time (i.e., > 12 weeks after event onset) to allow for corticosteroids (if initiated) to be reduced to ≤ 10 mg/day oral prednisone or equivalent. The acceptable length of the extended period of time must be agreed upon by the investigator and the Coordinating investigator.

[b] If corticosteroids have been initiated, they must be tapered over ≥ 1 month to ≤ 10 mg/day oral prednisone or equivalent before atezolizumab can be resumed.

[c] Resumption of atezolizumab may be considered in patients who are deriving benefit and have fully recovered from the immune-related event. Patients can be re-challenged with atezolizumab only after approval has been documented by both the investigator (or an appropriate delegate) and the Coordinating investigator.

10) Management Guidelines for Neurologic Disorders

Myasthenia gravis and Guillain-Barré syndrome have been observed with single agent atezolizumab. Patients may present with signs and symptoms of sensory and/or motor neuropathy. Diagnostic work-up is essential for an accurate characterization to differentiate between alternative aetiologies. Management guidelines for neurologic disorders are provided in table 10.

Table 10. Management Guidelines for Neurologic Disorders

Event	Management
Immune-related neuropathy, Grade 1	• Continue atezolizumab.<br>• Investigate aetiology.
Immune-related neuropathy, Grade 2	• Withhold atezolizumab for up to 12 weeks after event onset. [a]<br>• Investigate aetiology.<br>• Initiate treatment as per institutional guidelines.<br>• If event resolves to Grade 1 or better, resume atezolizumab. [b]<br>• If event does not resolve to Grade 1 or better while withholding atezolizumab, permanently discontinue atezolizumab and contact Coordinating investigator. [c]
Immune-related neuropathy, Grade 3 or 4	• Permanently discontinue atezolizumab and contact Coordinating investigator. [c]<br>• Initiate treatment as per institutional guidelines.
Myasthenia gravis and Guillain-Barré syndrome (any grade)	• Permanently discontinue atezolizumab and contact Coordinating investigator. [c]<br>• Refer patient to neurologist.<br>• Initiate treatment as per institutional guidelines.<br>• Consider initiation of 1–2 mg/kg/day oral or IV prednisone or equivalent.

[a] Atezolizumab may be withheld for a longer period of time (i.e., > 12 weeks after event onset) to allow for corticosteroids (if initiated) to be reduced to ≤ 10 mg/day oral prednisone or equivalent. The acceptable length of the extended period of time must be agreed upon by the investigator and the Coordinating investigator.

[b] If corticosteroids have been initiated, they must be tapered over ≥ 1 month to ≤ 10 mg/day oral prednisone or equivalent before atezolizumab can be resumed.

[c] Resumption of atezolizumab may be considered in patients who are deriving benefit and have fully recovered from the immune-related event. Patients can be re-challenged with atezolizumab only after approval has been documented by both the investigator (or an appropriate delegate) and the Coordinating investigator.

11) Management Guidelines for Immune-Related Meningoencephalitis

Immune-related meningoencephalitis is an identified risk associated with the administration of atezolizumab. Immune-related meningoencephalitis should be suspected in any patient presenting with signs or symptoms suggestive of meningitis or encephalitis, including, but not

limited to, headache, neck pain, confusion, seizure, motor or sensory dysfunction, and altered or depressed level of consciousness. Encephalopathy from metabolic or electrolyte imbalances needs to be distinguished from potential meningoencephalitis resulting from infection (bacterial, viral, or fungal) or progression of malignancy, or secondary to a paraneoplastic process.

All patients being considered for meningoencephalitis should be urgently evaluated with a CT scan and/or MRI scan of the brain to evaluate for metastasis, inflammation, or oedema. If deemed safe by the treating physician, a lumbar puncture should be performed, and a neurologist should be consulted.

Patients with signs and symptoms of meningoencephalitis, in the absence of an identified alternate aetiology, should be treated according to the guidelines in table 11.

Table 11. Management Guidelines for Immune-Related Meningoencephalitis

Event	Management
Immune-related meningoencephalitis, all grades	• Permanently discontinue atezolizumab and contact Coordinating investigator. [a]<br>• Refer patient to neurologist.<br>• Initiate treatment with 1–2 mg/kg/day IV methylprednisolone or equivalent and convert to 1–2 mg/kg/day oral prednisone or equivalent upon improvement.<br>• If event does not improve within 48 hours after initiating corticosteroids, consider adding an immunosuppressive agent.<br>• If event resolves to Grade 1 or better, taper corticosteroids over ≥1 month.

[a] Resumption of atezolizumab may be considered in patients who are deriving benefit and have fully recovered from the immune-related event. Patients can be re-challenged with atezolizumab only after approval has been documented by both the investigator (or an appropriate delegate) and the Coordinating investigator.

12)Management Guidelines for Renal Events

Immune-related nephritis has been associated with the administration of atezolizumab. Eligible patients must have adequate renal function, and renal function, including serum creatinine, should be monitored throughout study treatment. Patients with abnormal renal function should be evaluated and treated for other more common aetiologies (including prerenal and postrenal causes, and concomitant medications such as non-steroidal anti-inflammatory drugs). Refer the patient to a renal specialist if clinically indicated. A renal biopsy may be required to enable a definitive diagnosis and appropriate treatment. If no alternative cause of acute kidney injury is identified, patients with signs and symptoms of acute kidney injury, in the absence of an identified alternate aetiology, should be treated according to the management guidelines for immune-related renal events in Table 12 below.

Table 12. Management Guidelines for Renal Events

Event	Management
Renal event, Grade 1	• Continue atezolizumab.<br>• Monitor kidney function, including creatinine, closely until values resolve to within normal limits or to baseline values.
Renal event, Grade 2	• Withhold atezolizumab for up to 12 weeks after event onset. [a]<br>• Refer patient to renal specialist.<br>• Initiate treatment with corticosteroids equivalent to 1–2 mg/kg/day oral prednisone.<br>• If event resolves to Grade 1 or better, resume atezolizumab. [b]<br>• If event does not resolve to Grade 1 or better while withholding atezolizumab, permanently discontinue atezolizumab and contact Coordinating investigator. [c]
Renal event, Grade 3 or 4	• Permanently discontinue atezolizumab and contact Coordinating investigator.<br>• Refer patient to renal specialist and consider renal biopsy.<br>• Initiate treatment with corticosteroids equivalent to 1–2 mg/kg/day oral prednisone.<br>• If event does not improve within 48 hours after initiating corticosteroids, consider adding an immunosuppressive agent.<br>• If event resolves to Grade 1 or better, taper corticosteroids over ≥ 1 month.

Note: Management guidelines are presented by adverse event severity based on NCI CTCAE and are applicable to both CTCAE Version 4.0 and CTCAE Version 5.0.

[a] Atezolizumab may be withheld for a longer period of time (i.e., > 12 weeks after event onset) to allow for corticosteroids (if initiated) to be reduced to the equivalent of ≤ 10 mg/day oral prednisone. The acceptable length of the extended period of time must be agreed upon by the investigator and the Coordinating investigator.

[b] If corticosteroids have been initiated, they must be tapered over ≥ 1 month to the equivalent of ≤ 10 mg/day oral prednisone before atezolizumab can be resumed.

[c] Resumption of atezolizumab may be considered in patients who are deriving benefit and have fully recovered from the immune-related event. Patients can be re-challenged with atezolizumab only after approval has been documented by both the investigator (or an appropriate delegate) and the Coordinating investigator.

13) Management Guidelines for Immune-Related Myositis

Immune-related myositis has been associated with the administration of atezolizumab. Myositis or inflammatory myopathies are a group of disorders sharing the common feature of inflammatory muscle injury; dermatomyositis and polymyositis are among the most common disorders. Initial diagnosis is based on clinical (muscle weakness, muscle pain, skin rash in dermatomyositis), biochemical (serum creatine kinase increase), and imaging (electromyography/MRI) features, and is confirmed with a muscle biopsy.

Patients with signs and symptoms of myositis, in the absence of an identified alternate aetiology, should be treated according to the guidelines in Table 13 below.

Table 13. Management Guidelines for Immune-Related Myositis

Event	Management
Immune-related myositi s, Grade 1	• Continue atezolizumab.<br>• Refer patient to rheumatologist or neurologist.<br>• Initiate treatment as per institutional guidelines.
Immune-related myositi s, Grade 2	• Withhold atezolizumab for up to 12 weeks after event onset [a] and contact Coordinating investigator.<br>• Refer patient to rheumatologist or neurologist.<br>• Initiate treatment as per institutional guidelines.<br>• Consider treatment with corticosteroids equivalent to 1–2 mg/kg/day IV methylprednisolone and convert to 1–2 mg/kg/day oral prednisone or equivalent upon improvement.<br>• If corticosteroids are initiated and event does not improve within 48 hours after initiating corticosteroids, consider adding an immunosuppressive agent.<br>• If event resolves to Grade 1 or better, resume atezolizumab. [b]<br>• If event does not resolve to Grade 1 or better while withholding atezolizumab, permanently discontinue atezolizumab and contact Coordinating investigator. [c]
Immune-related myositis, Grade 3	• Withhold atezolizumab for up to 12 weeks after event onset [a] and contact Coordinating investigator.<br>• Refer patient to rheumatologist or neurologist.<br>• Initiate treatment as per institutional guidelines. Respiratory support may be required in more severe cases.<br>• Initiate treatment with corticosteroids equivalent to 1-2 mg/kg/day IV methylprednisolone, or higher-dose bolus if patient is severely compromised (e.g., cardiac or respiratory symptoms, dysphagia, or weakness that severely limits mobility); convert to 1-2 mg/kg/day oral prednisone or equivalent upon improvement.<br>• If event does not improve within 48 hours after initiating corticosteroids, consider adding an immunosuppressive agent.<br>• If event resolves to Grade 1 or better, resume atezolizumab. [b]<br>• If event does not resolve to Grade 1 or better while withholding atezolizumab, permanently discontinue atezolizumab and contact Coordinating investigator. [c]<br>• For recurrent events, treat as a Grade 4 event.
Immune-related myositis, Grade 4	• Permanently discontinue atezolizumab and contact Coordinating investigator. [c]<br>• Refer patient to rheumatologist or neurologist.<br>• Initiate treatment as per institutional guidelines. Respiratory support may be required in more severe cases.<br>• Initiate treatment with corticosteroids equivalent to 1-2 mg/kg/day IV methylprednisolone, or higher-dose bolus if patient is severely compromised (e.g., cardiac or respiratory symptoms, dysphagia, or weakness that severely limits

	mobility); convert to 1-2 mg/kg/day oral prednisone or equivalent upon improvement.<br>• If event does not improve within 48 hours after initiating corticosteroids, consider adding an immunosuppressive agent.<br>• If event resolves to Grade 1 or better, taper corticosteroids over ≥ 1 month.

[a] Atezolizumab may be withheld for a longer period of time (i.e., > 12 weeks after event onset) to allow for corticosteroids (if initiated) to be reduced to the equivalent of ≤ 10 mg/day oral pr ednisone. The acceptable length of the extended period of time must be agreed upon by the investigator and the Coordinating investigator.

[b] If corticosteroids have been initiated, they must be tapered over ≥ 1 month to the equivalent of ≤ 10 mg/day oral prednisone before atezolizumab can be resumed.

[c] Resumption of atezolizumab may be considered in patients who are deriving benefit and have fully recovered from the immune-related event. Patients can be re-challenged with atezolizu mab only after approval has been documented by both the investigator (or an appropriate del egate) and the Coordinating investigator.

14) HEMOPHAGOCYTIC LYMPHOHISTIOCYTOSIS AND MACROPHAGE ACTIVATION SYNDROME

Immune-mediated reactions may involve any organ system and may lead to hemophagocytic lymphohistiocytosis (HLH) and macrophage activation syndrome (MAS), which are considered to be potential risks for atezolizumab. Patients with suspected HLH should be diagnosed according to published criteria by McClain and Eckstein (2014). A patient should be classified as having HLH if five of the following eight criteria are met:

- *Fever ≥ 38.5°C*
- *Splenomegaly*
- *Peripheral blood cytopenia consisting of at least two of the following:*
  - *Hemoglobin < 90 g/L (9 g/dL)*
  - *Platelet count < 100 × 10^9/L (100,000/μL)*
  - *ANC < 1.0 × 10^9/L (1000/μL)*
- *Fasting triglycerides > 2.992 mmol/L (265 mg/dL) and/or fibrinogen < 1.5 g/L (150 mg/dL)*
- *Hemophagocytosis in bone marrow, spleen, lymph node, or liver*
- *Low or absent natural killer cell activity*
- *Ferritin > 500 mg/L (500 ng/mL)*
- *Soluble interleukin 2 (IL-2) receptor (soluble CD25) elevated ≥ 2 standard deviations above age-adjusted laboratory-specific norms*

Patients with suspected MAS should be diagnosed according to published criteria for systemic juvenile idiopathic arthritis by Ravelli et al. (2016). A febrile patient should be classified as having MAS if the following criteria are met:

- *Ferritin > 684 mg/L (684 ng/mL)*
- *At least two of the following:*
  - *Platelet count ≤ 181 × 10^9/L (181,000/μL)*
  - *AST ≥ 48 U/L*
  - *Triglycerides > 1.761 mmol/L (156 mg/dL)*
  - *Fibrinogen ≤ 3.6 g/L (360 mg/dL)*

Patients with suspected HLH or MAS should be treated according to the guidelines in Table 14.

**Table 14.** *Management Guidelines for Suspected Hemophagocytic Lymphohistiocytosis or Macrophage Activation Syndrome* ***Event***	***Management***
Suspected HLH or MAS	• Permanently discontinue atezolizumab and contact Medical Monitor.<br>• Consider patient referral to hematologist.<br>• Initiate supportive care, including intensive care monitoring if indicated per institutional guidelines.<br>• Consider initiation of IV corticosteroids and/or an immunosuppressive agent.<br>• If event does not improve within 48 hours after initiating corticosteroids, consider adding an immunosuppressive agent.<br>• If event resolves to Grade 1 or better, taper corticosteroids over ≥ 1 month.

HLH = hemophagocytic lymphohistiocytosis; MAS = macrophage activation syndrome.

REFERENCES

McClain KL, Eckstein O. Clinical features and diagnosis of hemophagocytic lymphohistiocytosis. Up to Date [resource on the Internet]. 2014 [updated 29 October 2018; cited: 17 May 2019]. Available from: https://www.uptodate.com/contents/clinical-features-and-diagnosis-of-hemophagocytic-lymphohistiocytosis.

Ravelli A, Minoia F, Davi S, et al. 2016 classification criteria for macrophage activation syndrome complicating systemic juvenile idiopathic arthritis: a European League Against Rheumatism/American College of Rheumatology/Paediatric Rheumatology International Trials Organisation Collaborative Initiative. Ann Rheum Dis 2016;75:481–9.

19.5 Appendix 5. Pertuzumab and Trastuzumab related toxicity management

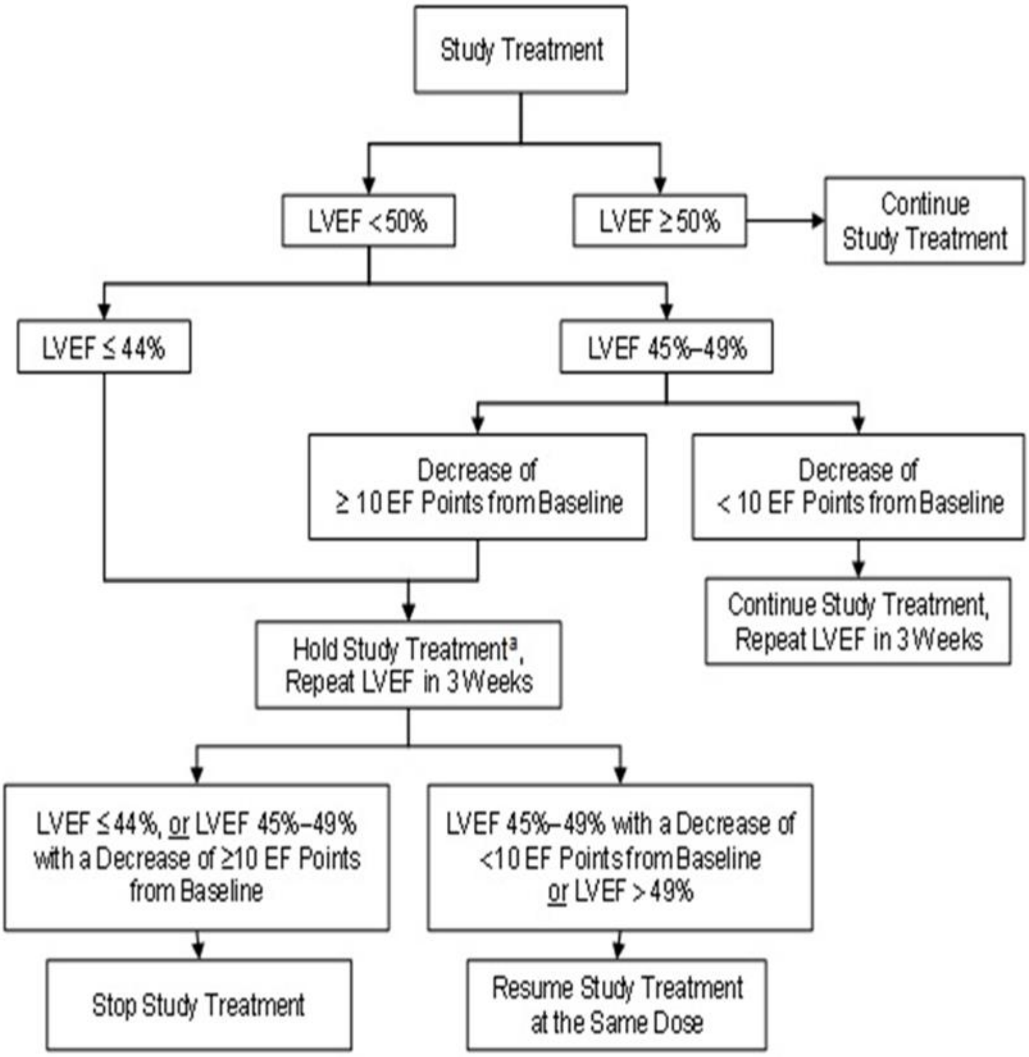

19.6 Table 15. Guidelines for Management of Adverse Events Associated with Trastuzumab Emtansine

Event	Action to Be Taken
Hematologic toxicity (cont.)	
Grade 2 or 3 thrombocytopenia on day of scheduled treatment	Assess platelet counts weekly or as medically indicated until recovery. Hold trastuzumab emtansine treatment until Grade ≤ 1. Resume treatment without dose reduction. If a patient requires 2 delays due to thrombocytopenia, consider reducing dose by one level.
Grade 4 thrombocytopenia at any time	Assess platelet counts weekly or as medically indicated until recovery. Hold trastuzumab emtansine until Grade ≤ 1, then resume with one dose level reduction (i.e., from 3.6 mg/kg to 3 mg/kg or from 3 mg/kg to 2.4 mg/kg) in subsequent cycles. If event occurs with 2.4 mg/kg dose, discontinue study treatment.
Hepatotoxicity	
ALT	For a Grade 2–3 ALT increase that occurs on the laboratory evaluation for cycle Day 1 or the planned day of dosing, hold trastuzumab emtansine until ALT recovers to Grade ≤1. Resume with dose reduction by one level for Grade 2 or 3 elevations. Grade 2–3 ALT elevations that are noted between cycles do not require dose delay or reduction unless ALT remains elevated (Grade ≥2) at the time of planned dosing.<br><br>For Grade 4 ALT increase, discontinue trastuzumab emtansine. Repeat laboratory evaluation (within 24 hours) may be performed to exclude laboratory error prior to discontinuing study treatment.
AST	For Grade 2 AST increase on the laboratory evaluation for cycle Day 1 or the planned day of dosing, hold trastuzumab emtansine until AST recovers to Grade ≤1. Resume without dose reduction when recovered.<br><br>For Grade 3 AST increase on the laboratory evaluation for cycle Day 1 or the planned day of dosing, hold trastuzumab emtansine until AST recovers to Grade ≤1. Resume with dose reduction by one level when recovered.<br><br>For Grade 4 AST increase, discontinue trastuzumab emtansine. Repeat laboratory evaluation (within 24 hours) may be performed to exclude laboratory error prior to discontinuing study treatment.

Event	Action to Be Taken
Hepatotoxicity (cont.)	
TBILI	For TBILI > 1.0 × ULN to ≤ 2.0 × ULN that occurs on the laboratory evaluation for cycle Day 1 or the day of planned dosing, hold trastuzumab emtansine until TBILI recovers to ≤ 1.0 × ULN (or direct bilirubin recovers to ≤ 1.0 × ULN for patients with Gilbert's syndrome). For TBILI elevations > 1.0 × ULN to ≤ 2.0 × ULN, resume when recovered with a one level dose reduction.<br><br>For TBILI > 2 × ULN at any time (or direct bilirubin > 2 × ULN for Gilbert's syndrome), discontinue trastuzumab emtansine and report the event as an SAE (if applicable) or non-serious expedited AE (if applicable).<br><br>Assess AST, ALT, and TBILI weekly or as medically indicated until recovery. Allow a maximum dose delay of 42 days from the last administered dose to recovery as described above or otherwise discontinue study treatment.
Nodular Regenerative Hyperplasia	For any clinical signs of liver dysfunction, discontinue trastuzumab emtansine and have the patient evaluated by a hepatologist.<br>If there are signs of portal hypertension (e.g., ascites and/or varices) and a cirrhosis-like pattern is seen on CT scan of the liver, the possibility of NRH should be considered. Trastuzumab emtansine should be discontinued in the event of a diagnosis of NRH.
Neurotoxicity	
Grade ≥ 3 peripheral neuropathy	Discontinue trastuzumab emtansine if event does not resolve to Grade ≤ 2 or baseline value within 42 days after the last administered dose.
Cardiotoxicity	
LVSD	Refer to **오류! 참조 원본을 찾을 수 없습니다.** for the algorithm for continuation and discontinuation of study treatment on the basis of asymptomatic LVEF assessment.
Grade 3–4 LVSD or Grade 3–4 heart failure	Discontinue study treatment.
Grade 2 heart failure accompanied by LVEF < 45%	Discontinue study treatment.

Event	Action to Be Taken
Interstitial lung disease	
Grade 3–4 pneumonitis	Discontinue study treatment regardless of attribution.
Grade 1–2 pneumonitis	Discontinue study treatment if not radiotherapy-related. For symptomatic (Grade 2) radiotherapy-related pneumonitis, discontinue if not resolving with standard treatment (e.g., steroids). Relationship to radiotherapy should be determined on the basis of

Event	Action to Be Taken
	timing and location of radiographic abnormalities relative to the radiation treatment.<br>Upon diagnosis of drug-related ILD/pneumonitis, trastuzumab emtansine treatment has to be permanently discontinued.
Radiotherapy-related skin toxicity	
Grade 3–4	Do not administer study treatment until recovery to Grade ≤ 1.

AE = adverse event; CBC = complete blood count; CT = computed tomography; ILD = interstitial lung disease; LVEF = left ventricular ejection fraction; LVSD = left ventricular systolic dysfunction; NRH = nodular regenerative hyperplasia; SAE = serious adverse event; ULN = upper limit of normal.

19.7 Guidelines for Management of Adverse Events that are Potential Overlapping Toxicities Associated with Trastuzumab Emtansine in Combination with Atezolizumab

PULMONARY EVENTS

Dyspnea, cough, fatigue, hypoxia, pneumonitis, and pulmonary infiltrates have been associated with the administration of atezolizumab and have primarily been observed in patients with underlying non–small cell lung cancer.

Mild to moderate events of pneumonitis have been reported with atezolizumab. All pulmonary events should be thoroughly evaluated for other commonly reported etiologies such as pneumonia/infection, lymphangitic carcinomatosis, pulmonary embolism, heart failure, chronic obstructive pulmonary disease, or pulmonary hypertension:

- Measurement of oxygen saturation (i.e., arterial blood gas)
- High-resolution CT scan of the chest
- Bronchoscopy with bronchoalveolar lavage and biopsy
- Pulmonary function tests (diffusion capacity of the lung for carbon monoxide)
- Pulmonary function testing with a pulmonary embolism protocol

Patients will be assessed for pulmonary signs and symptoms throughout the study. See Table 16 for management guidelines for pulmonary events and pneumonitis.

Table 16. Management Guidelines for Interstitial Lung Disease and Pneumonitis

Severity	Atezolizumab	Trastuzumab Emtansine
Grade 1–2	Grade 2: withhold treatment and initiate corticosteroids.	Discontinue study treatment if not radiotherapy-related. For symptomatic (Grade 2) radiotherapy-related pneumonitis, discontinue if not resolving with standard treatment (e.g., steroids).
Grade 3–4	Discontinue atezolizumab/placebo treatment.	Discontinue trastuzumab emtansine treatment.

Hepatic Events

Immune-mediated hepatitis has been associated with the administration of atezolizumab. Eligible patients must have adequate liver function, as manifested by measurements of total bilirubin and hepatic transaminases. Liver function will be monitored throughout study treatment.

While on this study, patients who present with right upper-quadrant abdominal pain and/or unexplained nausea or vomiting should have liver function tests (LFTs) performed immediately and reviewed before administration of the next dose of study drug.

If outcome of LFTs is worsening, concurrent medications, viral hepatitis, and toxic or neoplastic etiologies should be considered and addressed, as appropriate. Imaging of the liver, gall bladder, and biliary tree should be performed to rule out neoplastic or other causes for worsening outcome of LFTs. Anti-nuclear antibody, perinuclear anti-neutrophil cytoplasmic antibody, anti-liver kidney microsomal antibodies, and anti-smooth muscle antibody tests should be performed if an autoimmune etiology is considered. See Table 17 for management guidelines for increased transaminases (AST/ALT) and hepatic events. No dose modification for atezolizumab is indicated on the basis of hyperbilirubinemia alone.

Table 17 Management Guidelines for Increased Transaminases (AST/ALT) and Hepatic Events

Severity	Atezolizumab	Trastuzumab Emtansine
ALT or AST increase that meets Hy's Law criteria: ALT or AST $> 3 \times$ ULN in combination with TBILI $> 2 \times$ ULN or clinical jaundice	Discontinue atezolizumab treatment.	Discontinue trastuzumab emtansine treatment.

Severity	Atezolizumab	Trastuzumab Emtansine
ALT/AST Grade 2 (> 3.0–5.0 × ULN)	Withhold atezolizumab dose.<br><br>If persists > 5–7 days: Consider starting 1–2 mg/kg/day prednisone or equivalent per day; when recover to Grade ≤ 1, taper steroids over ≥ 1 month.<br><br>Resume therapy when systemic steroid dose is ≤ 10mg oral prednisone equivalent per day and when recovered to Grade ≤ 1 within 12 weeks.<br><br>Permanently discontinue atezolizumab and contact the Coordinating investigator if event does not resolve to Grade ≤ 1 within 12 weeks.	Withhold trastuzumab emtansine dose until recovery to Grade ≤ 1. Resume with dose reduction by one level when recovered from Grade 2 ALT elevation. No dose reduction needed after recovery from Grade 2 AST elevation.
ALT/AST Grade 3 (> 5.0–20.0 × ULN)	Discontinue atezolizumab treatment.<br><br>Consider GI consult and liver biopsy to establish etiology of hepatic injury if necessary.<br><br>Start 60 mg prednisone or equivalent per day.<br><br>If LFT results do not decrease within 48 hours after initiation of systemic steroids, addition of an alternative immunosuppressive agent (e.g., mycophenolate or	Withhold trastuzumab emtansine dose until recovery to Grade ≤ 1. Resume with dose reduction by one level when recovered.

Severity	Atezolizumab	Trastuzumab Emtansine
	TNF-α antagonist) may be considered.<br><br>Taper steroids over ≥ 1 month, when symptoms improve to Grade 0 or Grade 1.	
ALT/AST Grade 4 (> 20.0 × ULN)	Discontinue atezolizumab treatment.<br><br>Consider GI consult and liver biopsy to establish etiology of hepatic injury if necessary.<br><br>Start 60 mg prednisone or equivalent per day.<br><br>If LFT results do not decrease within 48 hours after initiation of systemic steroids, addition of an alternative immunosuppressive agent (e.g., mycophenolate or TNF-α antagonist) may be considered.<br><br>Taper steroids over ≥ 1 month, when symptoms improve to Grade 0 or Grade 1.	Discontinue trastuzumab emtansine treatment. Laboratory tests may be repeated (within 24 hours) to exclude laboratory error prior to discontinuing trastuzumab emtansine.

Severity	Atezolizumab	Trastuzumab Emtansine
NRH<br><br>If there are signs of portal hypertension (e.g., ascites and/or varices) and/or a cirrhosis-like pattern is seen on a CT scan of the liver, the possibility of NRH should be considered.	Discontinue atezolizumab treatment.	For any clinical signs of liver dysfunction, discontinue trastuzumab emtansine and have the patient evaluated by a hepatologist. If there are signs of portal hypertension (e.g., ascites and/or varices) and a cirrhosis-like pattern is seen on CT scan of the liver, the possibility of NRH should be considered. Trastuzumab emtansine should be discontinued in the event of a diagnosis of NRH.

CT = computed tomography; GI = gastrointestinal; LFT = liver function test; NRH = Nodular Regenerative Hyperplasia; TNF = tumor necrosis factor; ULN = upper limit of normal.

ULN = upper limit of normal.

19.8. IMP administration guideline

To use filter line	Atezolizumab, Trastuzumab emtansine
IMP administration and process	[Neo-adjuvant therapy]<br>D1 Pertuzumab 840mg loading(only C1)+ NS 250mL MIV over 60mins (only C1D1)<br>420mg NS 250mL MIV over 30-60min C2-C6 D1 IV<br>D1 Atezolizumab 1200mg IV + NS 250 ml over 60 (±15)mins<br>D1 Docetaxel 75mg/m2 IV + D5W 200ml miv over 1hr<br>D1 Trastuzumab 600mg SC<br><br>Q3weeks<br>Premedication *<br>D1 Avil(=Chlorpheniramine) 1A + D5W 50mL MIV 30mins before Pertuzumab<br>* Dexamethasone 8mg PO (total 6 doses) related Docetaxel<br>D0 Night (X1),<br>D1 immediately upon waking in the morning<br>1 hour before Docetaxel infusion, Night (X3)<br>D2 Morning, Evening<br><br>-Administration sequence<br>Atezolizumab → Pertuzumab → Docetaxel → Trastuzumab<br>C1 : Atezolizumab(1hr) →premedi(30min) → Pertuzumab(1hr) -> 60 min obs → Docetaxel(1hr) → Trastuzumab(SC)<br>No infusion related event at C1<br>C2~: Atezolizumab(30min) → premedi(30min) → Pertuzumab(30min) -> 30 min obs → Docetaxel(1hr) → Trastuzumab(SC)<br><br>[Adjuvant therapy]<br>If PCR PAH q 3weeks Adjuvant C1~C12<br>D1 Pertuzumab 840mg loading(only C1)+ NS 250mL MIV over 60mins (only C1D1)<br>420mg NS 250mL MIV over 30-60min C2-C12 D1 IV<br>D1 Atezolizumab 1200mg IV + NS 250 ml over 60 (±15)mins<br>D1 Trastuzumab 600mg SC<br>Q

	3weeks<br><br>-Administration sequence<br>Atezolizumab(30min)→ premedi(30min) → Pertuzumab(30min) -> 30min obs → Trastuzumab(SC)<br><br>If non-PCR AK q 3weeks Adjuvant C1~C14<br>D1 Atezolizumab 1200mg IV + NS 250 ml over 60 (±15)mins<br>- infusion with 0.2 micron in-line filter<br>D1 Trastuzumab emtansine 3.6 mg/kg + NS 250 mL IV over 30-90 mins<br>- infusion with 0.2 micron in-line filter<br><br>Q3weeks<br>- -Administration sequence<br>Atezolizumab → Trastuzumab emtansine<br>1st: Atezolizumab(1hr) → Trastuzumab emtansine(90min) -> obs 90min<br>No infusion related event at C1<br>2nd~: Atezolizumab(30min)→ Trastuzumab emtansine(30min) -> obs 30min

* IMP administration guidelines may follow the site's standard procedure if they differ from the site's standard procedures.
