# Supplementary material for: Response Rate and Safety of a Neoadjuvant Pertuzumab, Atezolizumab, Docetaxel, and Trastuzumab Regimen for Patients With ERBB2-Positive Stage II/III Breast Cancer: The Neo-PATH Phase 2 Nonrandomized Clinical Trial
Source: JAMA Oncol. 2022 Jul 7;8(9):1271–7. doi: 10.1001/jamaoncol.2022.2310 (PMC10881214; doi:10.1001/jamaoncol.2022.2310)
Supplement: Supplement 2. — eAppendix eTable. Residual tumor burden in patients with non-pathological complete response [file jamaoncol-e222310-s002.pdf]

Supplemental Online Content

Ahn HK, Sim SH, Suh KJ, et al. Response rate and safety of a neoadjuvant pertuzumab, atezolizumab, docetaxel, and trastuzumab regimen for patients with *ERBB2*-positive stage II/III breast cancer: the Neo-PATH phase 2 nonrandomized clinical trial. *JAMA Oncol*. Published online July 7, 2022. doi:10.1001/jamaoncol.2022.2310

eAppendix

eTable. Residual tumor burden in patients with non-pathological complete response

This supplemental material has been provided by the authors to give readers additional information about their work.

© 2022 Ahn HK et al. *JAMA Oncol.*

eAppendix

Patients Characteristics

Among 16 patients with clinical stage IIA in our study, the number of patients with T2N0M0 was 13, and 11 among 13 patients had clinical T≤3cm and N0.

We have analyzed clinical characterictics of PD-L1+ patients. Age was significantly higher in patients with PD-L1 expression than in patients without PD-L1 expression (mean 58.7 years vs 50.9 years, $p<0.004$). PD-L1 expression were more prevalent among ER-negative tumors (27.0% among ER-negative tumor vs 9.7% among ER-positive tumors). All 13 PD-L1+ tumors had negative PR expression. PD-L1 expression was prevalent among earlier stage, 23%[11/48] of stage II were PD-L1+ disease, compared to only 11.8%[2/18] of stage III were PD-L1-positive disease.

© 2022 Ahn HK et al. *JAMA Oncol.*

eTable. Residual tumor burden in patients with non-pathological complete response		
Characteristics	Number N=24	%
Residual tumor size		
ypT1	17	70.8%
ypT2	6	25.0%
ypT3	1	4.2%
Residual LN stage		
ypN0	15	62.5%
ypN1	8	33.3%
ypN2	1	4.2%
yp-stage		
I	11	45.8%
II	12	50.0%
III	1	4.2%
Residual cancer burden		
Class I	8	33.3%
Class II	13	54.2%
Class III	3	12.5%

© 2022 Ahn HK et al. *JAMA Oncol.*
