# Supplementary material for: Response Rate and Safety of a Neoadjuvant Pertuzumab, Atezolizumab, Docetaxel, and Trastuzumab Regimen for Patients With ERBB2-Positive Stage II/III Breast Cancer: The Neo-PATH Phase 2 Nonrandomized Clinical Trial
Source: JAMA Oncol. 2022 Jul 7;8(9):1271–7. doi: 10.1001/jamaoncol.2022.2310 (PMC10881214; doi:10.1001/jamaoncol.2022.2310)
Supplement: Supplement 3. — Data Sharing Statement [file jamaoncol-e222310-s003.pdf]

Data Sharing Statement

Ahn. Response Rate and Safety of a Neoadjuvant Pertuzumab, Atezolizumab, Docetaxel, and Trastuzumab Regimen for Patients With ERBB2-Positive Stage II/III Breast Cancer. *JAMA Oncol*. Published July 07, 2022. doi:10.1001/jamaoncol.2022.2310

Data
Data available: No
